# A computational method for the identification of candidate drugs for non-small cell lung cancer

**DOI:** 10.1371/journal.pone.0183411

**Published:** 2017-08-18

**Authors:** Lei Chen, Jing Lu, Tao Huang, Yu-Dong Cai

**Affiliations:** 1 College of Life Science, Shanghai University, Shanghai, People’s Republic of China; 2 College of Information Engineering, Shanghai Maritime University, Shanghai, People’s Republic of China; 3 School of Pharmacy, Key Laboratory of Molecular Pharmacology and Drug Evaluation (Yantai University), Ministry of Education, Collaborative Innovation Center of Advanced Drug Delivery System and Biotech Drugs in Universities of Shandong, Yantai University, Yantai, People’s Republic of China; 4 Institute of Health Sciences, Shanghai Institutes for Biological Sciences, Chinese Academy of Sciences, Shanghai, People’s Republic of China; University of South Alabama Mitchell Cancer Institute, UNITED STATES

## Abstract

Lung cancer causes a large number of deaths per year. Until now, a cure for this disease has not been found or developed. Finding an effective drug through traditional experimental methods invariably costs millions of dollars and takes several years. It is imperative that computational methods be developed to integrate several types of existing information to identify candidate drugs for further study, which could reduce the cost and time of development. In this study, we tried to advance this effort by proposing a computational method to identify candidate drugs for non-small cell lung cancer (NSCLC), a major type of lung cancer. The method used three steps: (1) preliminary screening, (2) screening compounds by an association test and a permutation test, (3) screening compounds using an EM clustering algorithm. In the first step, based on the chemical-chemical interaction information reported in STITCH, a well-known database that reports interactions between chemicals and proteins, and approved NSCLC drugs, compounds that can interact with at least one approved NSCLC drug were picked. In the second step, the association test selected compounds that can interact with at least one NSCLC-related chemical and at least one NSCLC-related gene, and subsequently, the permutation test was used to discard nonspecific compounds from the remaining compounds. In the final step, core compounds were selected using a powerful clustering algorithm, the EM algorithm. Six putative compounds, protoporphyrin IX, hematoporphyrin, canertinib, lapatinib, pelitinib, and dacomitinib, were identified by this method. Previously published data show that all of the selected compounds have been reported to possess anti-NSCLC activity, indicating high probabilities of these compounds being novel candidate drugs for NSCLC.

## 1. Introduction

Lung cancer is a major cause of cancer-related deaths worldwide [1], and the number of deaths has shown an increasing trend over the past fifteen years [2] despite improvements in research and development (R&D) and increased investments in R&D. Therefore, drug discovery for treating lung cancers important. Lung cancers comprise two major types, non-small cell lung cancer (NSCLC) and small cell lung cancer (SCLC). NSCLC accounts for more than 85% of lung cancer cases [3], and most approved drugs, such as gefitinib, cisplatin and paclitaxel, are used to treat NSCLC.

Experimental testing during drug R&D costs millions of dollars and takes several years, and only a few drugs meet the activity and safety requirements for regulatory approval. In silico methods for early assessment are attractive for improving the success rates and reducing the costs of R&D. Many previous studies based on in silico predictions have been carried out to analyze the structure-activity relationships (SARs) of anti-NSCLC chemicals and identify promising chemicals that can act as substitutes for approved NSCLC drugs. Lang *et al*. reported that the cytotoxic activities of cucurbitacins against NSCLC A549 cells were associated with their propensity for electrophilic attack, molecular size and shape in a QSAR study [4]. Goyal *et al*. developed a 3D-QSAR model using 38 thiazolyl-pyrazoline compounds against EGFR, which is a target associated with NSCLC, and obtained two novel inhibitors by screening ZINC libraries [5]. Xiang *et al*. proposed a novel “hybrid strategy” by using a chemically reactive feature and a pharmacophore feature and identified 38 irreversible EGFR-T790M inhibitors [6]. The above methods primarily used the structures of chemicals to discover compounds that have anti-NSCLC activity. Recently, Lu *et al*. developed a novel computational model by using chemical-/protein-chemical interaction information and identified promising chemicals with potential anti-NSCLC activity that were structurally dissimilar to drugs approved for NSCLC [7]. However, the effectiveness of the method was not very high. Of the nineteen compounds identified, only six were found to have anti-NSCLC activity. Although this method needs to be improved, the concept of identifying drug candidates by integrating chemical-/protein-chemical interactions is a suitable approach. Since there are various mutations in different genes, a drug will only be effective if it can target the appropriate disease genes. From the perspective of precision medicine, different patients should receive different treatment regimens. If protein-chemical interactions are characterized during drug screening, drugs, once validated and approved, can be prescribed to patients of a certain subtype for a more precise treatment. Therefore, we tried to extend this method by using additional related information and more powerful computational tools.

In this study, we proposed an improved computational method for the identification of novel candidate drugs of NSCLC. For execution and analysis, sixteen approved NSCLC drugs, NSCLC-related chemicals, NSCLC-related genes and chemical-/protein-chemical interactions were retrieved from public websites and databases. The method consisted of three steps. In the first step, namely, preliminary screening, possible compounds were extracted by checking the chemical-chemical interactions involving approved NSCLC drugs. In the second step, these compounds were filtered by an association test and a permutation test, where the association test helps us select compounds that have associations with both NSCLC-related chemicals and NSCLC-related genes, while the permutation test can exclude nonspecific compounds that are not associated with NSCLC. Finally, the remaining compounds were analyzed using a cluster algorithm, the EM clustering algorithm, to further select core compounds. As a result, six compounds, protoporphyrin IX, hematoporphyrin, canertinib, lapatinib, pelitinib, and dacomitinib, were identified. Data from previously published reports indicate that all of these compounds have anti-NSCLC activity, implying that there is a high probability that they may be candidate drugs for NSCLC. Moreover, canertinib, lapatinib, pelitinib, and dacomitinib were confirmed to be effective for NSCLC associated with mutations in *EGFR*, which can help formulate guidelines for the precise medical treatment of NSCLC involving these mutations.

## 2. Materials and methods

### 2.1 Approved NSCLC drugs and chemicals as well as genes related to NSCLC

#### 2.1.1 Approved NSCLC drugs

We accessed sixteen approved NSCLC drugs from the following two websites: (1) http://www.cancer.gov/cancertopics/druginfo/lungcancer (accessed in January 2016); (2) http://www.medindia.net/drugs/medical-condition/lungcancer.htm (accessed on May 11, 2014). Detailed information about these sixteen drugs, including their mechanism [8–23], is provided in **Table 1**.

**Table 1:** Sixteen approved NSCLC drugs.

PubChem ID	Name	Mechanism	NSCLC-related Genes
CID4033	Mechlorethamine	Agents directly acting on DNA	*CASP9*, *ERBB2*, *ERCC1*, *TP53*
CID36314	Paclitaxel	Antimitotic agent	*ABCB1*, *ABCB11*, *AKT1*, *BIRC5*, *CSF3*, *EGFR*, *ERBB2*, *KIF5B*, *TP53*, *VEGFA*
CID38904	Carboplatin	Agents directly acting on DNA	*ABCB1*, *AKT1*, *ALK*, *AREG*, *AREGB*, *BIRC5*, *CASP8*, *CASP9*, *CCND1*, *CDKN2A*, *CSF3*, *EGFR*, *EML4*, *ERBB2*, *ERCC1*, *GSTP1*, *HRAS*, *KRAS*, *MDM2*, *MET*, *NQO1*, *NRAS*, *PIK3CA*, *PIK3CB*, *RASSF1*, *RRM1*, *STAT3*, *STK4*, *TGFA*, *TP53*, *VEGFA*, *VEGFC*
CID57166	Porfimer Sodium	Photosensitizing agent	*PRDX1*
CID60750	Gemcitabine	Agents interfering with DNA synthesis	*-*
CID60780	Vinorelbine	Antimitotic agent	*-*
CID60843	Pemetrexed	Agents interfering with DNA synthesis	*AKT1*, *ALK*, *EGFR*, *EML4*, *ERBB2*, *ERCC1*, *HRAS*, *KRAS*, *NRAS*, *RRM1*, *TP53*, *VEGFA*
CID123631	Gefitinib	EGFR inhibitor	*ABCB1*, *ABCB11*, *AKT1*, *AKT2*, *ALK*, *AREG*, *AREGB*, *AXL*, *BIRC5*, *CASP8*, *CCND1*, *CDK4*, *CDKN2A*, *E2F1*, *EGFR*, *EML4*, *ERBB2*, *ERCC1*, *FASLG*, *FGFR1*, *FOXM1*, *FOXO3*, *GRB2*, *HBEGF*, *HRAS*, *KRAS*, *MAPK1*, *MAPK10*, *MAPK13*, *MAPK14*, *MAPK3*, *MDM2*, *MET*, *MMP9*, *NRAS*, *PIK3CA*, *PIK3CB*, *PTHLH*, *RASSF1*, *RB1*, *STAT3*, *STK4*, *TERT*, *TGFA*, *TP53*, *VEGFA*, *VEGFC*
CID126941	Methotrexate	Agents interfering with DNA synthesis	*ABCB1*, *ABCB11*, *AKT1*, *ALK*, *CASP8*, *CCND1*, *CD93*, *CDKN2A*, *CSF3*, *DNMT3L*, *EGFR*, *ENO1*, *ERBB2*, *FASLG*, *GRB2*, *HRAS*, *IL10*, *IL6R*, *LILRB1*, *MAPK14*, *MDM2*, *NCOA3*, *PROS1*, *RB1*, *TP53*, *TRAF1*, *VEGFA*
CID148124	Docetaxel	Antimitotic agent	*-*
CID176870	Erlotinib	EGFR inhibitor	*ABCB1*, *AKT1*, *ALK*, *AREG*, *AREGB*, *AXL*, *BIRC5*, *CCND1*, *CDKN2A*, *EGF*, *EGFR*, *EML4*, *ERBB2*, *ERCC1*, *FASLG*, *GRB2*, *HBEGF*, *HRAS*, *KRAS*, *MAPK1*, *MAPK3*, *MET*, *NRAS*, *PIK3CA*, *PIK3CB*, *STAT3*, *STK4*, *TGFA*, *TP53*, *VEGFA*, *VEGFC*
CID441203	Cisplatin	Agents directly acting on DNA	*-*
CID5360373	Bleomycin	Agents directly acting on DNA	*-*
CID10184653	Afatinib	EGFR/HER2/HER4 inhibitor	*ALK*, *EGF*, *EGFR*, *EML4*, *ERBB2*, *HRAS*, *IL6R*, *KRAS*, *MET*, *NRAS*, *PIK3CA*
CID11626560	Crizotinib	ALK/HGFR inhibitor	*AKT1*, *ALK*, *AXL*, *BIRC5*, *EGF*, *EGFR*, *EML4*, *ERBB2*, *HBEGF*, *HRAS*, *KIF5B*, *KRAS*, *MAPK1*, *MAPK3*, *MET*, *NRAS*, *PIK3CA*, *ROS1*, *STAT3*, *STK4*, *TGFA*
CID57379345	Ceritinib	ALK antagonist	*-*

#### 2.1.2 NSCLC-related chemicals

A total of 3,793 NSCLC-related chemicals were downloaded from the Comparative Toxicogenomics Database (CTD) (http://ctdbase.org/detail.go?type=disease&acc=MESH:D002289&view=chem, accessed in March 2015) [24], for which the disease and chemical relationships were manually extracted from the literature. After mapping these chemicals to their PubChem IDs, 3,085 chemicals were retained; these chemicals comprised the dataset *S*_*c*_ and are listed in S1 Table.

#### 2.1.3 NSCLC-related genes

We identified NSCLC-related genes using the following two public databases: (1) Kyoto Encyclopedia of Genes and Genomes (KEGG, http://www.genome.jp/kegg/) [25, 26]; (2) CTD [24]. More specifically, from KEGG, 54 genes associated with NSCLC-related pathways were retrieved (accessed in February 2014), and from CTD, we identified 104 NSCLC-related genes for which there was direct evidence of association with NSCLC (accessed in March 2015). After combining these two sets of NSCLC-related genes, 148 genes were obtained; these genes comprised the dataset *S*_*g*_ and are listed in S2 Table.

### 2.2 Chemical-/protein-chemical interaction

The basis of our method for identifying candidate drugs for NSCLC is to discover compounds that have similar functions as approved NSCLC drugs and close relationships with NSCLC-related chemicals and genes. To implement the method, we mined databases for chemical-chemical interactions and protein-chemical interactions. This section provides a brief description of our approach.

#### 2.2.1 Chemical-chemical interaction

This information was retrieved from the Search Tool for Interactions of Chemicals (STITCH, http://stitch.embl.de/) [27], a well-known public database that catalogs large numbers of interactions between chemicals and proteins. Chemicals are linked to other chemicals according to the evidence derived from experiments, databases and the literature. This type of chemical-chemical interaction information is widely used to investigate several biological problems [7, 28–36]. We downloaded a file, named “chemical_chemical.links.detailed.v4.0.tsv.gz”, from STITCH (Version 4.0), which lists large numbers of chemical-chemical interactions. For each interaction, there are two PubChem IDs and five scores labeled “Similarity”, “Experimental”, “Database”, “Textmining” and “Combined_score”, respectively. The “Similarity”, “Experimental”, “Database”, and “Textmining” scores are obtained by examining the structures, activities, reactions and co-occurrence in the literature of chemicals, respectively. Finally, the “Combined_score” was determined by integrating all of the aforementioned scores. To formulate this mathematically, let us denote the above five scores for chemicals *c*_1_ and *c*_2_ using $Q_{S}^{cc}(c_{1},c_{2}),Q_{E}^{cc}(c_{1},c_{2}),Q_{D}^{cc}(c_{1},c_{2}),Q_{T}^{cc}(c_{1},c_{2})$ and $Q_{C}^{cc}(c_{1},c_{2})$. Because the “Combined_score” can widely indicate associations between chemicals, it was used here to indicate the interactiveness of two chemicals, *i*.*e*., two chemicals were deemed to interact with each other if and only if the “Combined_score” between them was greater than zero.

#### 2.2.2 Protein-chemical interaction

In addition to chemical-chemical interactions, STITCH also contains information on protein-chemical interactions. This information has also been applied to investigate many biological problems [7, 36–41]. We downloaded the file “protein_chemical.links.detailed.v4.0.tsv.gz” from this database (Version 4.0), in which the interactions between chemicals and proteins from 1,133 organisms were collected. From the obtained file, we extracted the interactions involving human proteins by selecting lines containing “9606” that is the code of Homo sapiens in STITCH. For each extracted interaction, there is one chemical, represented by a PubChem ID; one protein, represented by an Ensembl ID; and five scores, labeled “Experimental”, “Prediction”, “Database”, “Textmining” and “Combined_score”, respectively. To formulate this mathematically, we used $Q_{E}^{pc}(p,c),Q_{P}^{pc}(p,c),Q_{D}^{pc}(p,c),Q_{T}^{pc}(p,c)$ and $Q_{C}^{pc}(p,c)$ to denote the scores between protein *p* and chemical *c*. As above, the “Combined_score” was used to define the interactiveness of chemicals and proteins, *i*.*e*., a chemical and a protein were deemed to interact with each other if and only if their “Combined_score” was greater than zero.

### 2.3 Method for identification of novel candidate drugs for NSCLC

**Fig 1:**
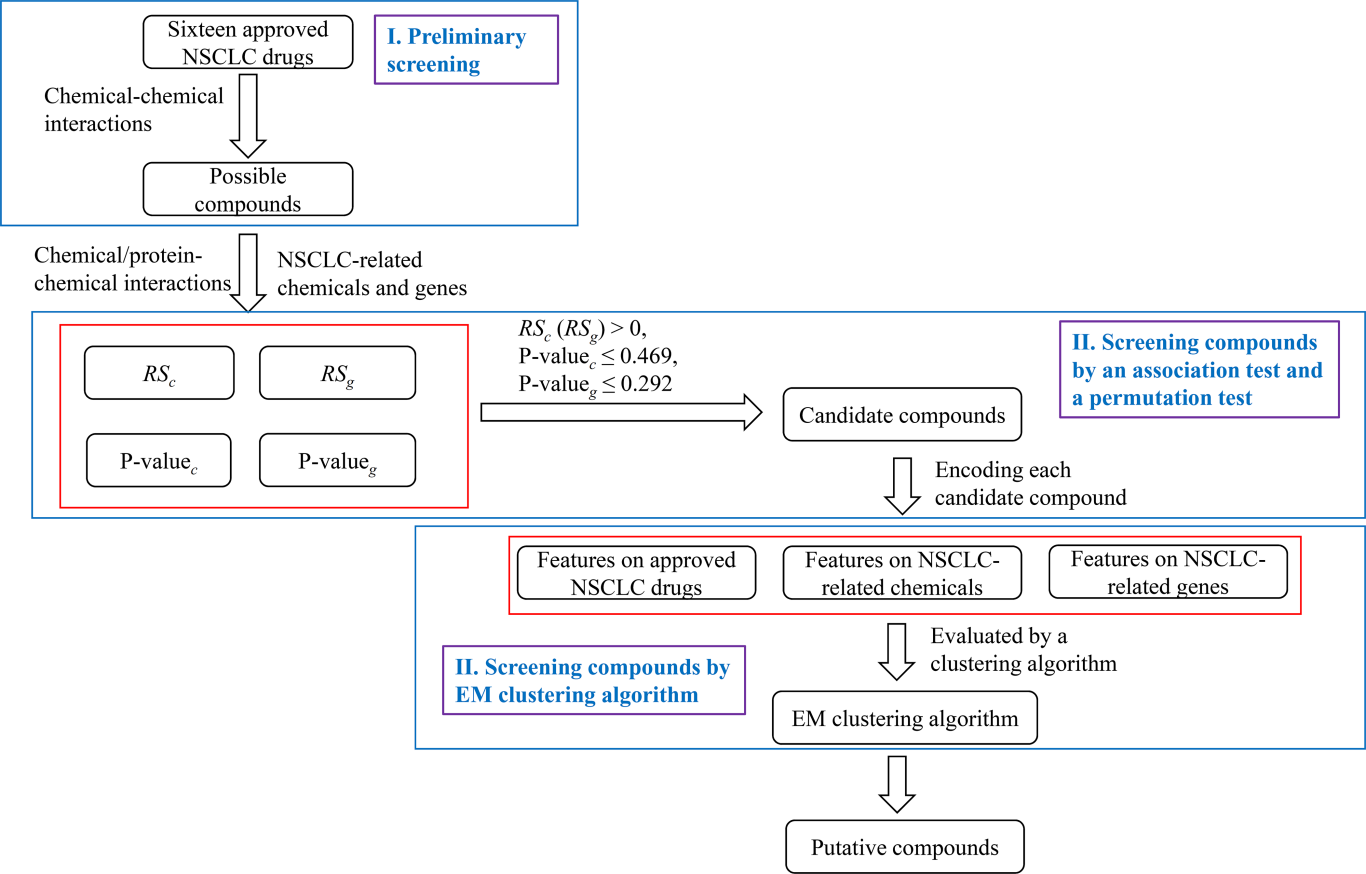
The flow chart of the computational method.

This section provides a detailed description of the computational method for the identification of candidate drugs for NSCLC. This method consisted of three steps: (1) preliminary screening, (2) screening compounds by an association test and a permutation test, and (3) screening compounds by an EM clustering algorithm. A flow chart of the method is illustrated in **Fig 1** and the pseudo codes are provided in **Table 2**.

**Table 2:** The pseudo codes of the method for identification of novel candidate drugs for NSCLC.

**Input:** Approved NSCLC drugs, NSCLC-related chemicals *S*_*c*_, NSCLC-related genes *S*_*g*_, chemical/protein-chemical interactions retrieved from STITCH
**Output:** A list of putative compounds
1. Preliminary screening
1.1 Using the approved NSCLC drugs and chemical-chemical interactions, compounds that can interact with at least one approved NSCLC drug were obtained and comprised the set *P*_*c*_.
2. Screening compounds by an association test and a permutation test
2.1 For each compound *c* in *P*_*c*_, if it can interact with at least one NSCLC-related chemical and one NSCLC-related gene (i.e., *RS*_*c*_(*c*)>0 and *RS*_*g*_(*c*)>0), it was selected.
2.2 For each compound *c* obtained in step 2.1, do the permutation test and calculate the P-value for NSCLC-related chemicals P − value_*c*_(*c*) (cf. Eq 1) as well as P-value for NSCLC-related genes P – value_*g*_(*c*) (cf. Eq 2)
2.3 If a compound *c* was assigned P − value_*c*_(*c*) and P – value_*g*_(*c*) no more than the corresponding threshold, it was selected.
3. Screening compounds by the EM clustering algorithm
3.1 For each compound obtained in step 2, it was represented by fifteen features based on the interaction scores of protein/chemical-chemical interactions.
3.2 For each approved drug, it was also represented by fifteen features used in step 3.1.
3.3 The remaining compounds and approved drugs were fed into the EM clustering algorithm.
3.4 According to the clustering results, compounds in the same category as approved drugs were picked and outputted.

#### 2.3.1 Preliminary screening

Many studies have reported that compounds that can interact with each other invariably share similar functions [7, 28–35]. As mentioned in Section 2.1.1, sixteen approved NSCLC drugs were retrieved from public websites. The compounds that can interact with these drugs are more likely to be potential drugs for NSCLC than those that cannot interact with any of them. Therefore, we obtained lists of compounds that can interact with at least one approved NSCLC drug by mining the chemical-chemical interactions database as described in Section 2.2.1. This list of compounds comprised a compound set denoted by *P*_*c*_ for convenience.

#### 2.3.2 Screening compounds by an association test and a permutation test

After the preliminary screening, several possible compounds were obtained. It was almost impossible to test them individually through traditional experiments, and therefore, further screening was required. It was easy to determine if a candidate drug was closely related to the biological processes associated with NSCLC, including its related genes and chemicals. Here, we built an association test and a permutation test to screen for relevant compounds in *P*_*c*_.

In the association test, compounds that had associations with NSCLC-related chemicals/genes were selected. Each compound *c* in *P*_*c*_ was linked to all NSCLC-related chemicals in *S*_*c*_ using chemical-chemical interactions as described in Section 2.2.1. If *c* can interact with at least one NSCLC-related chemical, then it was selected because it is highly related to at least one NSLC-related chemical. On the other hand, we also screened compounds in *P*_*c*_ using NSCLC-related genes in *S*_*g*_. More specifically, each compound *c* in *P*_*c*_ was linked to all of the NSCLC-related genes in *S*_*g*_ using protein-chemical interactions as described in Section 2.2.2. The compounds that could interact with at least one gene in *S*_*g*_ were selected. By considering both the NSCLC-related chemicals and genes, we selected compounds in *P*_*c*_ that could interact with at least one NSCLC-related chemical and gene. Additionally, for a later evaluation of the importance of the selected compound *c*, we extracted all of the chemical-chemical interactions between *c* and NSCLC-related chemicals. The mean value of the “Combined_score” of these interactions was calculated, which was called the rating score of *c* for NSCLC-related chemicals and denoted by *RS*_*c*_(*c*). Similarly, we also extracted all of the protein-chemical interactions between *c* and NSCLC-related genes. The mean value of their “Combined_score” was calculated. This value was called the rating score of *c* for NSCLC-related genes and denoted by *RS*_*g*_(*c*). In a formalization way, we selected compounds in *P*_*c*_ with *RS*_*c*_>0 and *RS*_*g*_>0 in the association test.

The association test helps us select compounds that have associations with NSCLC-related chemicals/genes. However, some compounds may have unusual properties and may interact nonspecifically with several compounds and genes. However, they may not be linked to NSCLC. Therefore, we built a permutation test to evaluate each compound that passed the association test and excluded these types of compounds. Let *c* be a compound that passed the association test. For the NSCLC-related chemical set *S*_*c*_, we randomly produced 1,000 sets with the same size as *S*_*c*_, which were denoted as $S_{c}^{1},S_{c}^{2},\ldots ,S_{c}^{1000}$. For each set $S_{c}^{i}$ (*i* = 1,2,…,1000), we calculated the rating score of *c* using the procedures mentioned above. Thus, *c* was assigned one rating score based on its associations in *S*_*c*_ and an additional 1,000 rating scores based on the sets $S_{c}^{1},S_{c}^{2},\ldots ,S_{c}^{1000}$. A parameter, namely, the P-value for NSCLC-related chemicals, was calculated for *c* by using the formula

$$P-{\mathrm{value}}_{c}(c)=\frac{W_{c}}{1000}$$ (1)

where *W*_*c*_ is the number of sets for which the rating scores were larger than *RS*_*c*_(*c*). Furthermore, we also evaluated the importance of compounds that passed the association test using the NSCLC-related gene set *S*_*g*_. One thousand sets, $S_{g}^{1},S_{g}^{2},\ldots ,S_{g}^{1000}$, were randomly constructed, and each of them had the same size as *S*_*g*_. As above, we also calculated the rating score of compound *c* for each set and computed the P-value of *c* for NSCLC-related genes, denoted by P – value_*g*_(*c*), as

$$P-{\mathrm{value}}_{g}(c)=\frac{W_{g}}{1000}$$ (2)

where *W*_*g*_ represents the number of sets for which the rating scores were larger than *RS*_*g*_(*c*). Generally, we should select compounds with low P-values for NSCLC-related chemicals/genes. However, it is quite difficult to determine the thresholds of these two P-values. On the other hand, the P-values of approved NSCLC drugs were important indicators, which helped us select proper thresholds. Therefore, we computed the P-values of approved NSCLC drugs for NSCLC-related chemicals and genes. To retain as many candidate compounds as possible and avoid missing potential compounds, we selected the maximum values from the P-values of approved NSCLC drugs as the thresholds of the two P-values.

#### 2.3.3 Screening compounds by the EM clustering algorithm

Some candidate compounds were able to pass the association test and permutation test. These compounds have many or few associations with NSCLC. A procedure was built to screen core candidate compounds that have extensive associations with approved NSCLC drugs, NSCLC-related chemicals and NSCLC-related genes from this set.

**2.3.3.1** **Feature extraction**: As described in Section 2.2, five scores for chemical-chemical interactions and five scores for protein-chemical interactions were introduced. However, the first two procedures used only the last score. Here, all scores were used to extract useful features, which can accurately measure the associations between candidate compounds and approved NSCLC drugs, NSCLC-related chemicals or NSCLC-related genes. For each candidate compound *c*, fifteen features were extracted, of which five features were from the five scores for chemical-chemical interactions between *c* and approved NSCLC drugs, five features were from the five scores for chemical-chemical interactions between *c* and NSCLC-related chemicals and the last five features were from the five scores for protein-chemical interactions between *c* and NSCLC-related genes. Of the five features derived from the five scores of chemical-chemical interactions between *c* and approved NSCLC drugs, we only described how to extract a feature from the “Similarity” score; the others can be obtained in a similar way. Let *d*_1_,*d*_2_,…,*d*_*l*_ be approved NSCLC drugs such that $Q_{S}^{cc}(c,d_{i})>0$ (*i* = 1,2,…,*l*). The mean value of these scores was taken as a feature. Particularly, if *l* = 0, then this feature was set to zero. For the five features derived from the five scores of chemical-chemical interactions between *c* and NSCLC-related chemicals, each of them can be obtained in a similar fashion as the feature mentioned above. Finally, of the five features derived from protein-chemical interactions between *c* and NSCLC-related genes, we only provide a description of features derived from the “Experimental” score; others can be constructed in a similar way. Let *g*_1_,*g*_2_,…,*g*_k_ be NSCLC-related genes with $Q_{E}^{pc}(g_{i},c)>0$ (*i* = 1,2,…,*k*). Then, the mean value of these scores was counted as a feature. Additionally, it was set to zero if *k* = 0. Furthermore, each approved NSCLC drug was also encoded by these fifteen features described above. All of the candidate compounds and approved NSCLC drugs were subsequently fed into a clustering algorithm.

**2.3.3.2** **EM clustering algorithm****:** The EM algorithm, proposed by Dempster *et al*. [42], is an iterative method to find the maximum likelihood of parameters in statistical models. The iteration procedure alternates between executing an expectation (E) step and a maximization (M) step. Its steps are listed in **Table 3**. If the dataset obeys a distribution that can be approximated by a mixture of Gaussian distributions, the EM algorithm can be extended to clustering. The unobserved data set *Z* represents which Gaussian the datum in observed data set *Y* comes from. By utilizing the EM algorithm, the parameters of each Gaussian can be estimated, which helps to assign each datum to a particular one.

**Table 3:** The procedures of the EM algorithm.

EM algorithm
**Input:** an observed dataset *Y*, an unobserved latent data set *Z*, the joint distribution *P*(*Y*,*Z*|*θ*) and the conditional distribution *P*(*Z*|*Y*,*θ*)
**Output:** parameter θ*
(1) Set an initial parameter *θ*^(0)^ and *i* = *0*
(2) E-step: for the current estimated value *θ*^(*i*)^ of parameter *θ*, calculate the objective function: $Q(\theta ,\theta^{\left(i\right)})=E_{Z|Y,\theta^{\left(i\right)}}[\log P(Y,Z|\theta )]=\underset{Z}{\sum }\log P(Y,Z|\theta )P(Z|Y,\theta^{\left(i\right)})$
(3) M-step: the new estimated value of parameter *θ*, say *θ*^(*i*+1)^, by maximizing *Q*(*θ*,*θ*^(*i*)^), i.e., $\theta^{\left(i+1\right)}=\arg\;\underset{\theta }{\max}Q(\theta ,\theta^{\left(i\right)})$
(4) Check the condition ‖*θ*^*i*+1^−*θ*^*i*^‖_2_ < *ε*. If the condition is not satisfied, *i* = *i* + 1, go to (2); else set θ* = *θ*^(*i*+1)^ and output θ*.

Weka [43] is a suite of software collecting several popular state-of-the-art machine learning algorithms and data preprocessing tools. The “EM” tool implements the EM clustering algorithm described above. For convenience, it was directly employed in this study to cluster the candidate compounds and approved NSCLC drugs. The default parameters were used to execute “EM” in which the class number can be automatically determined. Based on the cluster results, candidate compounds in the same category as approved NSCLC drugs were picked, and these were called putative compounds for convenience.

## 3. Results and discussion

### 3.1 Results of the preliminary screening

Sixteen approved NSCLC drugs were used in this study. In the preliminary screening procedure, we extracted all compounds that can interact with at least one approved NSCLC drug, obtaining 3,261 possible compounds. These compounds are listed in S3 Table.

### 3.2 Results of the association test and permutation test

Several possible compounds were identified in the preliminary screening procedure. Clearly, not all of them have anti-NSCLC activity. In the association test, they were linked to NSCLC-related chemicals and NSCLC-related genes. Those that can interact with at least one NSCLC-related chemical and one NSCLC-related gene were kept, resulting in 1,281 compounds. In addition, we calculated the rating scores for NSCLC-related chemicals (cf. *RS*_*c*_) and NSCLC-related genes (cf. *RS*_*g*_) for each of the 1,281 compounds. These scores are available in S3 Table. It is necessary to note that the sixteen approved NSCLC drugs were also examined in the association test. The results show that ten of them can interact with at least one NSCLC-related chemical and one NSCLC-related gene. They are listed in **Table 4**. Additionally, the two rating scores were also calculated and are listed in **Table 4**. These ten drugs helped us to further screen important compounds.

**Table 4:** The measurements of ten approved NSCLC drugs yielded by the computational method.

PubChem ID	Name	Rating score on NSCLC-related chemicals	Rating score on NSCLC-related genes	P-value on NSCLC-related chemicals	P-value on NSCLC-related genes
CID4033	Mechlorethamine	296.299	358.250	0.248	0.115
CID36314	Paclitaxel	306.485	462.889	0.346	0.221
CID38904	Carboplatin	307.897	332.267	0.196	0.238
CID57166	Porfimer Sodium	307.231	218.000	0.312	0.032
CID60843	Pemetrexed	278.798	233.750	0.337	0.124
CID123631	Gefitinib	303.047	493.275	0.289	0.292
CID126941	Methotrexate	330.201	362.250	0.469	0.150
CID176870	Erlotinib	320.090	579.929	0.273	0.175
CID10184653	Afatinib	365.038	451.091	0.128	0.063
CID11626560	Crizotinib	293.407	579.500	0.263	0.187

For the permutation test, we calculated the P-values for NSCLC-related chemicals (cf. **Eq 1**) and NSCLC-related genes (cf. **Eq 2**) for each of the 1,281 compounds that passed the association test; these are provided in S3 Table. Furthermore, these two P-values were also computed for the ten approved NSCLC drugs and are listed in **Table 4**. The maximum P-value of the ten approved NSCLC drugs for NSCLC-related chemicals was 0.469, and the maximum P-value of the ten approved NSCLC drugs for NSCLC-related genes was 0.292. Accordingly, 0.469 and 0.292 were set as the thresholds for the P-values for NSCLC-related chemicals and NSCLC-related genes, respectively, *i*.*e*., we selected the compounds with P-values less than or equal to 0.469 for NSCLC-related chemicals and P-values less than or equal to 0.292 for NSCLC-related genes. Based on these thresholds, 1,007 compounds were retained, which are listed in S4 Table.

### 3.3 Results of the EM clustering algorithm

To further select core candidate compounds from the 1,007 compounds obtained after the permutation test, they were represented by fifteen features, as described in Section 2.3.3. In addition, the ten approved NSCLC drugs listed in **Table 4** were also encoded in the same way. Next, the EM clustering algorithm was used to cluster these 1,017 compounds (1007 candidate compounds and ten approved NSCLC drugs). The results are provided in S5 Table. Four categories were built by the EM clustering algorithm. Notably, the ten approved NSCLC drugs were clustered in the same category (cluster3). Clearly, candidate compounds in this category are more likely to be novel drugs for NSCLC than other candidate compounds. Therefore, they were extracted, resulting in 98 candidate compounds, which are listed in S6 Table.

However, 98 candidate compounds are still too many to screen for potential drugs for NSCLC. Therefore, these compounds and the ten approved NSCLC drugs were again input into the EM clustering algorithm. The clustering results are available in S6 Table and show that five categories were used to cluster these compounds. Interestingly, the ten approved NSCLC drugs were still clustered in the same category (cluster3). Another six candidate compounds were also in this category and are listed in **Table 5**. These six putative compounds were deemed to be significant for further investigation.

**Table 5:** Detailed information of six putative compounds.

PubChem ID	Name	Rating score on NSCLC-related chemicals	P-value on NSCLC-related chemicals	Rating score on NSCLC-related genes	P-value on NSCLC-related genes	NSCLC-related Genes
CID4971	Protoporphyrin IX	347.889	0.326	437.000	0.115	*ABCB1*, *ABCB10*, *CASP8*, *CAT*, *SOD2*, *TP53*
CID11103	Hematoporphyrin	309.920	0.225	224.333	0.029	*MAP2K1*, *MAPK1*, *MAPK3*
CID156413	Canertinib	331.293	0.155	781.000	0.054	*AKT1*, *AREG*, *AREGB*, *EGF*, *EGFR*, *ERBB2*, *MAPK10*, *MMP9*, *VEGFA*
CID208908	Lapatinib	307.412	0.303	504.750	0.119	*ABCB1*, *ABCB11*, *AKT1*, *AREG*, *AREGB*, *BIRC5*, *CCND1*, *CLPTM1L*, *EGFR*, *ERBB2*, *FOXM1*, *HRAS*, *KRAS*, *MAPK1*, *MAPK3*, *MET*, *NRAS*, *PIK3CA*, *STAT3*, *TGFA*, *TP53*, *VEGFA*
CID6445562	Pelitinib	322.081	0.153	605.833	0.112	*AKT1*, *BIRC5*, *EGF*, *EGFR*, *ERBB2*, *STK4*
CID11511120	Dacomitinib	302.176	0.131	576.000	0.025	*EGFR*, *EML4*, *ERBB2*, *ERCC1*, *NRAS*

### 3.4 Analysis of significant candidate drugs

**Fig 2:**
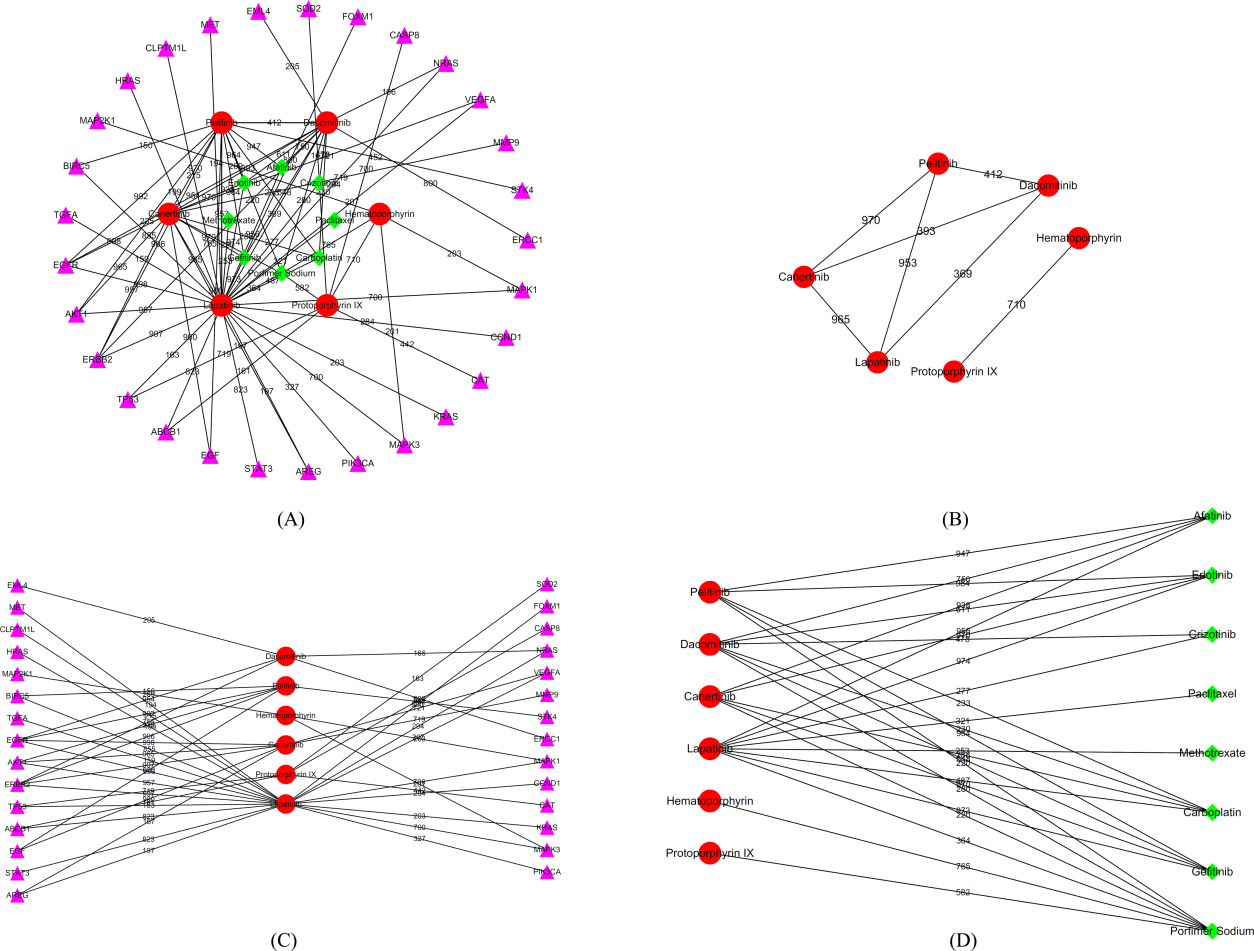
The interaction sub-network of putative compounds, NSCLC-related genes and approved drugs. Red circles represent putative compounds, pink triangles represent NSCLC-related genes, green diamonds represent approved drugs. Weights on edges are “Combined_score” of corresponding chemical-chemical interactions or protein-chemical interactions. (A) the whole sub-network; (B) the sub-network of putative compounds; (C) the sub-network of putative compounds and NSCLC-related genes; (D) the sub-network of putative compounds and approved drugs.

In this study, six putative compounds for NSCLC were identified by our method, which are listed in **Table 5**. To give their associations with approved drugs and NSCLC-related genes, a network consisting of the interactions among putative compounds, approved drugs and NSCLC-related genes was plotted in **Fig 2**. It can be observed that each putative compound is closely related to at least one approved drugs (see **Fig 2(D)**) and one NSCLC-related genes (see **Fig 2(C)**), suggesting that these putative compounds can be novel candidate drugs for NSCLC. In addition, the interactions between four putative compounds: Pelitinib, Dacomitinib, Canertinib and Lapatinib comprise a clique (see **Fig 2(B)**), a graph such that each pair of nodes is connected by an edge, implying they are highly related with each other. If one can be validated to be a novel drug for NSCLC, the rest putative compounds can be novel drugs with high probabilities. For other two putative compounds: Hematoporphyrin and Protoporphyrin IX, they can interact with each other, inducing the same results mentioned above. To give a more convincing explanation, a summary of the extensive data in the literature that support the anti-NSCLC activity of these compounds is presented below.

#### 3.4.1 Protoporphyrin IX (CID4971)

Photodynamic therapy (PDT) can be used for the treatment of different tumors [44]. 5-Aminolevulinic acid (ALA) is a pro-drug of the photosensitizer protoporphyrin IX (PPIX). ALA-mediated PDT showed photo-cytotoxicities towards H460 cell lines by activating p38 MAPK and JNK signal pathways [45]. Postiglione *et al*. reported that gefitinib combined with 5-ALA/PDT improved the response of NSCLC cell lines H1299 (p53-/-) and A549 (p53+/+) without EGFR mutations [46]. Zn PPIX by inhibiting heme oxygenase 1 reduced tumor growth of LL/2 mouse lung cancer cells [47]. Moreover, Zn PPIX increased the radiosensitivity of human NSCLC A549 cells and the cell apoptotic index when combined with irradiation [48].

#### 3.4.2 Hematoporphyrin (CID11103)

Hematoporphyrin and its derivatives can lead to induct DNA damage [49, 50]. Hematoporphyrin derivatives (HPD) are used for photodynamic therapy by selectively destroying malignant tumors, such as cancers of lung, digestive tract, and genitourinary tract [51]. LoCicero *et al*. reported that HPD decreased some symptoms of NSCLC patients, especially coughing [52]. Moreover, Edell *et al*. reported that 93% patients with early superficial squamous cell carcinoma achieved a complete response to HPD phototherapy and indicated that it may be an efficient alternative to surgical resection [53].

#### 3.4.3 Canertinib (CID156413)

Canertinib (Cl-1033) is a selective tyrosine kinase inhibitor (TKI) that blocks signal transduction through EGFRs [54]. Slichenmyer *et al*. reported that canertinib significantly suppressed the tumor growth of H125 NSCLC carcinoma [55]. Jänne *et al*. reported that canertinib had modest activity in advanced-stage NSCLC patients [56]. Moreover, canertinib was confirmed to be more effective than erlotinib and gefitinib against NSCLC cell lines with the EGFR L858R mutation and the EGFR L858R/T790M double mutations [57].

#### 3.4.4 Lapatinib (CID208908)

Lapatinib is a dual TKI of EGFR and the human epidermal receptor type 2 (ErbB2) receptor for treating advanced or metastatic breast cancer with the overexpression of ErbB2 protein [58]. Diaz *et al*. reported that lapatinib significantly reduced cell proliferation, DNA synthesis and colony formation in NSCLC A549 cells and inhibited the angiogenesis of tumors in mice [59]. Moreover, Kim *et al*. reported that the combination of lapatinib and cetuximab had enhanced cytotoxicity against gefitinib-resistant NSCLC cells [60]. Lapatinib also showed an inhibitory effect against the NSCLC cell line H3255 with the EGFR L858R mutation [57].

#### 3.4.5 Pelitinib (CID6445562)

Pelitinib (EKB-569) is a selective and irreversible inhibitor of EGFR. It showed clinical activity in two NSCLC patients with EGFR mutations and gefitinib resistance [61] and stabilized the disease in another NSCLC patient for 33 weeks [62]. Yoshimura *et al*. reported that pelitinib decreased multiple pulmonary metastases in two advanced NSCLC patients with EGFR mutations [61].

#### 3.4.6 Dacomitinib (CID11511120)

Dacomitinib (PF-00299804) is an irreversible pan-HER TKI that targets EGFRs. Ramalingam *et al*. reported that dacomitinib significantly improved progression-free survival compared with erlotinib in some clinical and molecular subsets, such as *KRAS* wild-type/*EGFR* wild-type and *EGFR* mutants [63]. However, the side effects of dacomitinib occurred more frequently and with greater intensity compared with erlotinib or gefitinib [64].

Of the above six putative compounds, canertinib, lapatinib, pelitinib, and dacomitinib may be promising for the treatment of NSCLC with *EGFR* mutations. Notably, only canertinib was identified in a previous study [7]. Therefore, the other newly identified compounds could be useful in future studies. Additionally, all of the compounds identified by the proposed method have been shown to possess anti-NSCLC activity. In a previous study, only 31.58% (6/19) of the identified compounds had anti-NSCLC activity. Therefore, it can be concluded that our method is effective at identifying candidate drugs for NSCLC.

## 4. Conclusions

This study used a computational method for identifying novel putative compounds of NSCLC, which were deemed to have anti-NSCLC activity. Several related materials, including chemical-chemical interactions, protein-chemical interactions, and the EM clustering algorithm were used for its implementation. Six compounds were identified, and further the analysis of the results indicated that all of them have anti-NSCLC activity. We hope that these newly identified compounds will be further validated by experimental data, which could lead to new therapies for treating NSCLC.

## Supporting information

S1 Table 3085 chemicals related to NSCLC. (DOCX)

S2 Table 148 genes related to NSCLC. (DOCX)

S3 Table 3261 possible compounds after preliminary screening. (PDF)

S4 Table 1007 candidate compounds filtered by the association test and permutation test. (PDF)

S5 Table Clustering results by the EM algorithm on 1007 candidate compounds and ten approved NSCLC drugs. (DOCX)

S6 Table Clustering results by the EM algorithm on 98 candidate compounds and ten approved NSCLC drugs. (DOCX)
