# Supplementary material for: A computational method for the identification of candidate drugs for non-small cell lung cancer
Source: PLoS One. 2017 Aug 18;12(8):e0183411. doi: 10.1371/journal.pone.0183411 (PMC5562320; doi:10.1371/journal.pone.0183411)
Supplement: S1 Table — (DOCX) [file pone.0183411.s001.docx]

**S1 Table.** 3085 chemicals related to NSCLC

| CID6 | CID5712 | CID19427 | CID108143 | CID5284371 |
| --- | --- | --- | --- | --- |
| CID11 | CID5717 | CID19592 | CID108156 | CID5284461 |
| CID13 | CID5722 | CID20279 | CID108162 | CID5284469 |
| CID33 | CID5732 | CID20393 | CID108188 | CID5284474 |
| CID51 | CID5733 | CID20770 | CID108213 | CID5284513 |
| CID72 | CID5741 | CID20796 | CID114709 | CID5284596 |
| CID96 | CID5743 | CID20966 | CID114730 | CID5284627 |
| CID137 | CID5746 | CID21013 | CID114764 | CID5284645 |
| CID176 | CID5753 | CID21100 | CID114777 | CID5284648 |
| CID177 | CID5754 | CID21157 | CID114778 | CID5287477 |
| CID180 | CID5755 | CID21202 | CID114810 | CID5287620 |
| CID187 | CID5756 | CID21387 | CID114831 | CID5287844 |
| CID190 | CID5757 | CID21450 | CID114850 | CID5287969 |
| CID227 | CID5770 | CID21694 | CID114909 | CID5288209 |
| CID234 | CID5773 | CID21700 | CID115005 | CID5288382 |
| CID237 | CID5780 | CID21800 | CID115110 | CID5288600 |
| CID241 | CID5781 | CID21803 | CID115127 | CID5288670 |
| CID243 | CID5789 | CID21804 | CID115196 | CID5288674 |
| CID244 | CID5790 | CID21913 | CID115204 | CID5288826 |
| CID247 | CID5794 | CID22206 | CID115221 | CID5289418 |
| CID263 | CID5801 | CID22311 | CID115237 | CID5289501 |
| CID264 | CID5809 | CID22318 | CID115239 | CID5310939 |
| CID281 | CID5816 | CID22469 | CID115344 | CID5311035 |
| CID284 | CID5819 | CID22750 | CID115355 | CID5311044 |
| CID284 | CID5826 | CID22948 | CID115361 | CID5311053 |
| CID289 | CID5831 | CID23205 | CID116545 | CID5311068 |
| CID297 | CID5833 | CID23306 | CID119031 | CID5311128 |
| CID297 | CID5839 | CID23337 | CID119032 | CID5311211 |
| CID297 | CID5852 | CID23393 | CID119041 | CID5311211 |
| CID299 | CID5853 | CID23403 | CID119104 | CID5311263 |
| CID305 | CID5862 | CID23448 | CID119170 | CID5311325 |
| CID311 | CID5865 | CID23648 | CID119182 | CID5311333 |
| CID313 | CID5870 | CID23925 | CID119196 | CID5311345 |
| CID323 | CID5877 | CID23930 | CID119218 | CID5311365 |
| CID335 | CID5881 | CID23930 | CID119245 | CID5311498 |
| CID338 | CID5886 | CID23931 | CID119311 | CID5312120 |
| CID359 | CID5889 | CID23939 | CID119368 | CID5312122 |
| CID366 | CID5891 | CID23939 | CID119373 | CID5312137 |
| CID370 | CID5892 | CID23950 | CID119440 | CID5315892 |
| CID398 | CID5894 | CID23954 | CID119461 | CID5317750 |
| CID421 | CID5896 | CID23954 | CID119543 | CID5318517 |
| CID428 | CID5897 | CID23963 | CID119598 | CID5318997 |
| CID441 | CID5904 | CID23973 | CID120290 | CID5319022 |
| CID444 | CID5910 | CID23976 | CID121907 | CID5319493 |
| CID460 | CID5917 | CID23976 | CID121922 | CID5319502 |
| CID541 | CID5920 | CID23978 | CID122272 | CID5320315 |
| CID544 | CID5921 | CID23982 | CID122281 | CID5320692 |
| CID547 | CID5931 | CID23985 | CID122597 | CID5320946 |
| CID568 | CID5943 | CID23989 | CID122623 | CID5326739 |
| CID586 | CID5944 | CID23990 | CID122724 | CID5327147 |
| CID588 | CID5952 | CID23994 | CID122821 | CID5328127 |
| CID612 | CID5954 | CID24007 | CID122980 | CID5328135 |
| CID650 | CID5955 | CID24012 | CID123195 | CID5328779 |
| CID660 | CID5957 | CID24014 | CID123351 | CID5328940 |
| CID679 | CID5959 | CID24083 | CID123591 | CID5329098 |
| CID681 | CID5971 | CID24085 | CID123591 | CID5329099 |
| CID698 | CID5978 | CID24121 | CID123631 | CID5329102 |
| CID702 | CID5984 | CID24139 | CID123632 | CID5330548 |
| CID702 | CID5988 | CID24154 | CID123683 | CID5351132 |
| CID712 | CID5991 | CID24261 | CID123725 | CID5351165 |
| CID727 | CID5993 | CID24261 | CID123798 | CID5352062 |
| CID727 | CID5994 | CID24268 | CID123808 | CID5352425 |
| CID727 | CID5995 | CID24288 | CID123823 | CID5353269 |
| CID727 | CID5997 | CID24316 | CID123865 | CID5353272 |
| CID753 | CID6000 | CID24341 | CID123917 | CID5353275 |
| CID756 | CID6001 | CID24360 | CID123935 | CID5353431 |
| CID774 | CID6006 | CID24380 | CID123938 | CID5353432 |
| CID778 | CID6013 | CID24385 | CID123964 | CID5353439 |
| CID784 | CID6020 | CID24393 | CID123976 | CID5353447 |
| CID785 | CID6021 | CID24404 | CID124069 | CID5353456 |
| CID790 | CID6022 | CID24424 | CID124072 | CID5353484 |
| CID795 | CID6024 | CID24434 | CID124087 | CID5353562 |
| CID798 | CID6029 | CID24458 | CID124219 | CID5353574 |
| CID802 | CID6031 | CID24459 | CID124331 | CID5353758 |
| CID863 | CID6036 | CID24460 | CID124820 | CID5353800 |
| CID864 | CID6037 | CID24462 | CID124886 | CID5353827 |
| CID870 | CID6041 | CID24470 | CID124998 | CID5353853 |
| CID880 | CID6046 | CID24488 | CID125160 | CID5353864 |
| CID887 | CID6047 | CID24502 | CID126451 | CID5353895 |
| CID890 | CID6049 | CID24526 | CID126517 | CID5353940 |
| CID892 | CID6049 | CID24567 | CID126941 | CID5354495 |
| CID896 | CID6053 | CID24570 | CID127676 | CID5354618 |
| CID903 | CID6054 | CID24584 | CID128172 | CID5355130 |
| CID931 | CID6058 | CID24586 | CID128682 | CID5356793 |
| CID932 | CID6072 | CID24589 | CID128828 | CID5358116 |
| CID935 | CID6076 | CID24597 | CID129080 | CID5359268 |
| CID936 | CID6099 | CID24598 | CID129236 | CID5359273 |
| CID938 | CID6103 | CID24667 | CID129869 | CID5359405 |
| CID943 | CID6104 | CID24763 | CID130209 | CID5359464 |
| CID948 | CID6113 | CID24766 | CID130779 | CID5359476 |
| CID961 | CID6114 | CID24808 | CID130956 | CID5359485 |
| CID964 | CID6115 | CID24812 | CID131149 | CID5359596 |
| CID977 | CID6124 | CID24823 | CID131211 | CID5359597 |
| CID980 | CID6128 | CID24856 | CID131351 | CID5359974 |
| CID985 | CID6129 | CID24857 | CID132519 | CID5360373 |
| CID991 | CID6133 | CID24863 | CID133111 | CID5360545 |
| CID992 | CID6137 | CID24871 | CID133728 | CID5360696 |
| CID995 | CID6138 | CID24924 | CID134601 | CID5360741 |
| CID996 | CID6163 | CID24934 | CID134780 | CID5360835 |
| CID998 | CID6166 | CID24937 | CID134799 | CID5362032 |
| CID1014 | CID6167 | CID24947 | CID135411 | CID5362119 |
| CID1018 | CID6184 | CID24962 | CID135728 | CID5362124 |
| CID1032 | CID6197 | CID24965 | CID135926 | CID5362372 |
| CID1048 | CID6199 | CID25408 | CID137994 | CID5362422 |
| CID1049 | CID6207 | CID25418 | CID139298 | CID5368086 |
| CID1054 | CID6212 | CID25423 | CID141745 | CID5368397 |
| CID1057 | CID6213 | CID25477 | CID145068 | CID5368831 |
| CID1061 | CID6223 | CID25504 | CID145937 | CID5369129 |
| CID1066 | CID6226 | CID25517 | CID146329 | CID5371318 |
| CID1080 | CID6228 | CID25670 | CID146798 | CID5371562 |
| CID1102 | CID6230 | CID25674 | CID147439 | CID5372312 |
| CID1103 | CID6243 | CID25736 | CID148177 | CID5372405 |
| CID1119 | CID6249 | CID25892 | CID148185 | CID5375667 |
| CID1123 | CID6251 | CID25948 | CID148195 | CID5376979 |
| CID1135 | CID6253 | CID26041 | CID148201 | CID5380905 |
| CID1140 | CID6254 | CID26042 | CID150459 | CID5381226 |
| CID1145 | CID6256 | CID26077 | CID150847 | CID5382656 |
| CID1174 | CID6264 | CID26089 | CID151202 | CID5387599 |
| CID1175 | CID6278 | CID26105 | CID151289 | CID5388781 |
| CID1176 | CID6279 | CID26172 | CID151453 | CID5393152 |
| CID1179 | CID6279 | CID26177 | CID151932 | CID5447130 |
| CID1183 | CID6282 | CID26757 | CID153976 | CID5458428 |
| CID1188 | CID6293 | CID26879 | CID154084 | CID5458510 |
| CID1195 | CID6294 | CID26934 | CID154224 | CID5459840 |
| CID1220 | CID6303 | CID26945 | CID154256 | CID5460048 |
| CID1229 | CID6319 | CID27492 | CID155169 | CID5460341 |
| CID1234 | CID6325 | CID27880 | CID155712 | CID5460467 |
| CID1318 | CID6326 | CID27924 | CID156391 | CID5462222 |
| CID1322 | CID6327 | CID28015 | CID156414 | CID5462224 |
| CID1352 | CID6336 | CID28094 | CID156422 | CID5462328 |
| CID1367 | CID6338 | CID28179 | CID157991 | CID5464467 |
| CID1388 | CID6339 | CID28281 | CID159324 | CID5469318 |
| CID1401 | CID6342 | CID28417 | CID159325 | CID5469424 |
| CID1405 | CID6344 | CID28780 | CID159573 | CID5470187 |
| CID1464 | CID6348 | CID29080 | CID159588 | CID5471349 |
| CID1474 | CID6354 | CID29131 | CID159594 | CID5472495 |
| CID1480 | CID6359 | CID29327 | CID159603 | CID5472867 |
| CID1486 | CID6365 | CID29339 | CID159797 | CID5473480 |
| CID1530 | CID6366 | CID29539 | CID159832 | CID5478003 |
| CID1539 | CID6371 | CID29574 | CID159854 | CID5479529 |
| CID1574 | CID6410 | CID30137 | CID159881 | CID5479530 |
| CID1598 | CID6423 | CID30322 | CID160337 | CID5479537 |
| CID1606 | CID6436 | CID30323 | CID160355 | CID5484006 |
| CID1606 | CID6468 | CID30332 | CID160511 | CID5484725 |
| CID1613 | CID6549 | CID30623 | CID160512 | CID5484727 |
| CID1614 | CID6557 | CID31099 | CID160611 | CID5484743 |
| CID1615 | CID6559 | CID31101 | CID160749 | CID5486195 |
| CID1639 | CID6564 | CID31200 | CID160824 | CID5487161 |
| CID1645 | CID6569 | CID31217 | CID160954 | CID5487525 |
| CID1673 | CID6575 | CID31222 | CID161126 | CID5487654 |
| CID1674 | CID6579 | CID31254 | CID161272 | CID5488822 |
| CID1678 | CID6597 | CID31275 | CID161491 | CID5490139 |
| CID1686 | CID6613 | CID31304 | CID161597 | CID5491458 |
| CID1688 | CID6618 | CID31307 | CID161753 | CID5491930 |
| CID1694 | CID6623 | CID31356 | CID162083 | CID5494414 |
| CID1701 | CID6626 | CID31368 | CID162558 | CID5494449 |
| CID1719 | CID6629 | CID31369 | CID162771 | CID5497136 |
| CID1732 | CID6636 | CID31373 | CID162859 | CID5702105 |
| CID1752 | CID6658 | CID31378 | CID163001 | CID5702160 |
| CID1752 | CID6675 | CID31401 | CID163098 | CID5702238 |
| CID1756 | CID6683 | CID31402 | CID163659 | CID5702613 |
| CID1775 | CID6720 | CID31404 | CID163659 | CID5712057 |
| CID1780 | CID6734 | CID31423 | CID164448 | CID5717148 |
| CID1795 | CID6736 | CID31553 | CID164619 | CID5717801 |
| CID1825 | CID6741 | CID31553 | CID164825 | CID5742673 |
| CID1853 | CID6754 | CID31703 | CID168971 | CID5803652 |
| CID1880 | CID6758 | CID31721 | CID169132 | CID5809667 |
| CID1893 | CID6763 | CID31829 | CID170262 | CID5813717 |
| CID1923 | CID6781 | CID32014 | CID170300 | CID5858445 |
| CID1935 | CID6795 | CID32064 | CID171092 | CID5904780 |
| CID1967 | CID6796 | CID32343 | CID171249 | CID5924208 |
| CID1969 | CID6811 | CID32490 | CID171548 | CID5941539 |
| CID1978 | CID6817 | CID32744 | CID174174 | CID6051497 |
| CID1982 | CID6849 | CID32756 | CID176155 | CID6091659 |
| CID1983 | CID6860 | CID32798 | CID176158 | CID6101642 |
| CID1986 | CID6869 | CID33184 | CID176408 | CID6321351 |
| CID1988 | CID6881 | CID33294 | CID176870 | CID6326970 |
| CID1990 | CID6896 | CID33344 | CID177880 | CID6326970 |
| CID2044 | CID6901 | CID33557 | CID179337 | CID6326983 |
| CID2051 | CID6905 | CID33572 | CID183679 | CID6327182 |
| CID2078 | CID6914 | CID33613 | CID185617 | CID6328106 |
| CID2082 | CID6917 | CID33641 | CID185792 | CID6328182 |
| CID2083 | CID6917 | CID33743 | CID186907 | CID6328211 |
| CID2088 | CID6944 | CID33776 | CID191552 | CID6328537 |
| CID2090 | CID6947 | CID34007 | CID194391 | CID6335486 |
| CID2094 | CID6953 | CID34210 | CID202225 | CID6338572 |
| CID2100 | CID6989 | CID34230 | CID206044 | CID6364615 |
| CID2118 | CID7000 | CID34378 | CID208898 | CID6398761 |
| CID2119 | CID7005 | CID34466 | CID208901 | CID6398764 |
| CID2125 | CID7017 | CID34507 | CID208907 | CID6432906 |
| CID2126 | CID7019 | CID34698 | CID208908 | CID6433099 |
| CID2140 | CID7020 | CID34755 | CID213031 | CID6433110 |
| CID2141 | CID7047 | CID34756 | CID216239 | CID6433159 |
| CID2146 | CID7048 | CID35370 | CID216416 | CID6433207 |
| CID2153 | CID7050 | CID35703 | CID216468 | CID6433272 |
| CID2157 | CID7050 | CID36159 | CID220861 | CID6433873 |
| CID2160 | CID7054 | CID36187 | CID221493 | CID6434251 |
| CID2162 | CID7057 | CID36218 | CID222154 | CID6434253 |
| CID2165 | CID7090 | CID36242 | CID222528 | CID6435286 |
| CID2179 | CID7102 | CID36314 | CID222757 | CID6435402 |
| CID2194 | CID7111 | CID36314 | CID222786 | CID6435415 |
| CID2214 | CID7127 | CID36400 | CID223368 | CID6435810 |
| CID2218 | CID7146 | CID36462 | CID224011 | CID6436082 |
| CID2230 | CID7153 | CID36679 | CID227037 | CID6436133 |
| CID2236 | CID7158 | CID36709 | CID228987 | CID6436239 |
| CID2244 | CID7166 | CID36811 | CID232446 | CID6436247 |
| CID2247 | CID7187 | CID37034 | CID241903 | CID6436291 |
| CID2249 | CID7222 | CID37035 | CID246598 | CID6436393 |
| CID2256 | CID7237 | CID37036 | CID253602 | CID6437055 |
| CID2265 | CID7237 | CID37037 | CID253613 | CID6437063 |
| CID2286 | CID7239 | CID37123 | CID259776 | CID6437082 |
| CID2314 | CID7243 | CID37182 | CID261000 | CID6437357 |
| CID2331 | CID7247 | CID37183 | CID265237 | CID6437371 |
| CID2332 | CID7257 | CID37207 | CID275196 | CID6437387 |
| CID2333 | CID7261 | CID37248 | CID292101 | CID6437388 |
| CID2336 | CID7270 | CID37269 | CID300471 | CID6437836 |
| CID2336 | CID7271 | CID37463 | CID312145 | CID6437875 |
| CID2337 | CID7276 | CID37542 | CID321710 | CID6438135 |
| CID2341 | CID7280 | CID37720 | CID322968 | CID6438163 |
| CID2346 | CID7285 | CID37768 | CID327044 | CID6438330 |
| CID2347 | CID7298 | CID37807 | CID331775 | CID6438333 |
| CID2351 | CID7305 | CID38012 | CID337359 | CID6438354 |
| CID2353 | CID7340 | CID38017 | CID363978 | CID6438357 |
| CID2361 | CID7367 | CID38024 | CID374536 | CID6438672 |
| CID2365 | CID7406 | CID38258 | CID375084 | CID6438859 |
| CID2374 | CID7434 | CID38347 | CID379077 | CID6438982 |
| CID2375 | CID7440 | CID38439 | CID381091 | CID6439414 |
| CID2396 | CID7452 | CID38779 | CID383414 | CID6439420 |
| CID2399 | CID7473 | CID38853 | CID387042 | CID6439461 |
| CID2405 | CID7474 | CID39042 | CID387447 | CID6439528 |
| CID2437 | CID7476 | CID39147 | CID390798 | CID6439546 |
| CID2477 | CID7482 | CID39184 | CID390986 | CID6439929 |
| CID2478 | CID7489 | CID39185 | CID392622 | CID6440581 |
| CID2513 | CID7490 | CID39186 | CID394347 | CID6440755 |
| CID2519 | CID7498 | CID39214 | CID400769 | CID6440856 |
| CID2520 | CID7499 | CID39327 | CID400769 | CID6441461 |
| CID2533 | CID7500 | CID39385 | CID409301 | CID6444190 |
| CID2541 | CID7501 | CID39484 | CID409805 | CID6445533 |
| CID2554 | CID7501 | CID39562 | CID416228 | CID6445537 |
| CID2566 | CID7502 | CID39676 | CID428373 | CID6445562 |
| CID2578 | CID7516 | CID39763 | CID433294 | CID6445781 |
| CID2585 | CID7526 | CID39929 | CID439177 | CID6449854 |
| CID2662 | CID7550 | CID39985 | CID439224 | CID6450197 |
| CID2687 | CID7570 | CID40024 | CID439230 | CID6450460 |
| CID2703 | CID7577 | CID40463 | CID439260 | CID6450842 |
| CID2707 | CID7804 | CID40470 | CID439302 | CID6451060 |
| CID2708 | CID7809 | CID40486 | CID439497 | CID6451153 |
| CID2710 | CID7812 | CID40585 | CID439501 | CID6480642 |
| CID2719 | CID7814 | CID40634 | CID439533 | CID6483648 |
| CID2719 | CID7833 | CID40692 | CID440317 | CID6518171 |
| CID2725 | CID7839 | CID40828 | CID440473 | CID6536806 |
| CID2726 | CID7840 | CID40839 | CID440646 | CID6536864 |
| CID2730 | CID7845 | CID40846 | CID440667 | CID6539118 |
| CID2733 | CID7847 | CID40854 | CID440936 | CID6603901 |
| CID2734 | CID7850 | CID40973 | CID441074 | CID6603931 |
| CID2746 | CID7853 | CID41322 | CID441140 | CID6604200 |
| CID2749 | CID7855 | CID41428 | CID441207 | CID6610292 |
| CID2750 | CID7858 | CID41867 | CID441233 | CID6858240 |
| CID2754 | CID7859 | CID42016 | CID441300 | CID6912226 |
| CID2756 | CID7860 | CID42128 | CID441336 | CID6914273 |
| CID2763 | CID7862 | CID42616 | CID441477 | CID6915744 |
| CID2764 | CID7871 | CID43226 | CID441923 | CID6915835 |
| CID2771 | CID7888 | CID43234 | CID442009 | CID6918114 |
| CID2783 | CID7896 | CID43244 | CID442021 | CID6918115 |
| CID2787 | CID7903 | CID43264 | CID442195 | CID6918155 |
| CID2788 | CID7923 | CID43815 | CID442424 | CID6918281 |
| CID2794 | CID7929 | CID44259 | CID442428 | CID6918296 |
| CID2796 | CID7954 | CID47263 | CID442530 | CID6918403 |
| CID2797 | CID7961 | CID47289 | CID442583 | CID6918412 |
| CID2801 | CID7964 | CID47318 | CID442793 | CID6918454 |
| CID2802 | CID7966 | CID47576 | CID443295 | CID6918505 |
| CID2803 | CID7979 | CID47811 | CID443313 | CID6918531 |
| CID2812 | CID7993 | CID49384 | CID443495 | CID6918532 |
| CID2816 | CID8002 | CID50894 | CID443872 | CID6918562 |
| CID2818 | CID8009 | CID51040 | CID444008 | CID6918638 |
| CID2832 | CID8019 | CID51130 | CID444036 | CID6918689 |
| CID2879 | CID8029 | CID51605 | CID444254 | CID6918788 |
| CID2907 | CID8035 | CID53230 | CID444493 | CID6918837 |
| CID2912 | CID8051 | CID53232 | CID444539 | CID9543540 |
| CID2915 | CID8058 | CID53462 | CID444732 | CID9547215 |
| CID2950 | CID8058 | CID53462 | CID444795 | CID9548591 |
| CID2955 | CID8063 | CID53665 | CID444899 | CID9548633 |
| CID2969 | CID8076 | CID54360 | CID445049 | CID9548657 |
| CID2973 | CID8082 | CID54454 | CID445070 | CID9549235 |
| CID2993 | CID8113 | CID54456 | CID445154 | CID9549297 |
| CID2995 | CID8118 | CID54562 | CID445354 | CID9562060 |
| CID2998 | CID8133 | CID54579 | CID445420 | CID9576787 |
| CID3007 | CID8137 | CID54586 | CID445434 | CID9661141 |
| CID3016 | CID8141 | CID54891 | CID445639 | CID9794823 |
| CID3017 | CID8147 | CID55182 | CID445643 | CID9796304 |
| CID3019 | CID8149 | CID55245 | CID445713 | CID9797817 |
| CID3026 | CID8181 | CID55283 | CID445858 | CID9803963 |
| CID3030 | CID8182 | CID55329 | CID446155 | CID9804302 |
| CID3033 | CID8223 | CID55466 | CID446220 | CID9806921 |
| CID3035 | CID8259 | CID55473 | CID446284 | CID9815648 |
| CID3036 | CID8264 | CID57166 | CID446313 | CID9816401 |
| CID3039 | CID8266 | CID57469 | CID446378 | CID9818893 |
| CID3039 | CID8268 | CID60164 | CID446512 | CID9818919 |
| CID3051 | CID8343 | CID60198 | CID446541 | CID9821754 |
| CID3053 | CID8350 | CID60490 | CID446925 | CID9822753 |
| CID3071 | CID8362 | CID60613 | CID447043 | CID9823846 |
| CID3077 | CID8369 | CID60648 | CID447277 | CID9825149 |
| CID3080 | CID8370 | CID60700 | CID447466 | CID9826528 |
| CID3081 | CID8376 | CID60750 | CID447587 | CID9827273 |
| CID3082 | CID8378 | CID60755 | CID447715 | CID9829523 |
| CID3100 | CID8417 | CID60780 | CID447905 | CID9829523 |
| CID3101 | CID8418 | CID60787 | CID447912 | CID9831050 |
| CID3108 | CID8433 | CID60823 | CID448014 | CID9831137 |
| CID3117 | CID8449 | CID60825 | CID448042 | CID9832136 |
| CID3118 | CID8452 | CID60838 | CID448537 | CID9837935 |
| CID3120 | CID8461 | CID60843 | CID449069 | CID9839500 |
| CID3121 | CID8469 | CID60846 | CID449171 | CID9839519 |
| CID3136 | CID8478 | CID60923 | CID449241 | CID9843744 |
| CID3151 | CID8490 | CID60944 | CID449459 | CID9846230 |
| CID3177 | CID8515 | CID60953 | CID449459 | CID9848818 |
| CID3191 | CID8530 | CID60961 | CID451415 | CID9860972 |
| CID3194 | CID8549 | CID60963 | CID451489 | CID9864378 |
| CID3197 | CID8560 | CID61021 | CID451668 | CID9865808 |
| CID3198 | CID8575 | CID61247 | CID452543 | CID9869053 |
| CID3213 | CID8576 | CID61253 | CID455658 | CID9869142 |
| CID3220 | CID8606 | CID61300 | CID456214 | CID9870520 |
| CID3224 | CID8663 | CID61436 | CID456410 | CID9870584 |
| CID3229 | CID8722 | CID61440 | CID457193 | CID9871074 |
| CID3259 | CID8742 | CID61486 | CID460612 | CID9874758 |
| CID3261 | CID8785 | CID61560 | CID464205 | CID9874913 |
| CID3276 | CID8814 | CID61635 | CID472335 | CID9877306 |
| CID3278 | CID8815 | CID61671 | CID480775 | CID9881652 |
| CID3291 | CID8948 | CID61948 | CID490002 | CID9883002 |
| CID3293 | CID8955 | CID62155 | CID493570 | CID9884685 |
| CID3301 | CID8956 | CID62275 | CID498142 | CID9885817 |
| CID3308 | CID8969 | CID62276 | CID503737 | CID9887803 |
| CID3314 | CID8987 | CID62298 | CID516859 | CID9890888 |
| CID3330 | CID9001 | CID62314 | CID517055 | CID9893571 |
| CID3331 | CID9033 | CID62344 | CID517165 | CID9898279 |
| CID3334 | CID9034 | CID62395 | CID517277 | CID9900574 |
| CID3339 | CID9051 | CID62652 | CID518740 | CID9903786 |
| CID3343 | CID9062 | CID62893 | CID522325 | CID9904203 |
| CID3347 | CID9064 | CID62923 | CID542762 | CID9913268 |
| CID3352 | CID9119 | CID62957 | CID636374 | CID9915743 |
| CID3366 | CID9126 | CID62959 | CID637511 | CID9924775 |
| CID3371 | CID9128 | CID62965 | CID637563 | CID9926054 |
| CID3372 | CID9131 | CID62978 | CID637566 | CID9929138 |
| CID3380 | CID9135 | CID63002 | CID637568 | CID9929901 |
| CID3385 | CID9136 | CID63041 | CID637759 | CID9936230 |
| CID3386 | CID9137 | CID63090 | CID637760 | CID9950868 |
| CID3394 | CID9140 | CID64139 | CID637776 | CID9956119 |
| CID3397 | CID9142 | CID64143 | CID637796 | CID9958995 |
| CID3406 | CID9153 | CID64928 | CID637858 | CID9974694 |
| CID3410 | CID9154 | CID64944 | CID638011 | CID10017512 |
| CID3430 | CID9158 | CID64945 | CID638015 | CID10026462 |
| CID3433 | CID9161 | CID64961 | CID638024 | CID10032582 |
| CID3440 | CID9164 | CID64962 | CID638034 | CID10044441 |
| CID3446 | CID9171 | CID64965 | CID638072 | CID10069117 |
| CID3454 | CID9191 | CID64971 | CID638276 | CID10090750 |
| CID3458 | CID9195 | CID64981 | CID638877 | CID10093303 |
| CID3463 | CID9201 | CID64982 | CID639665 | CID10095180 |
| CID3467 | CID9223 | CID64989 | CID640429 | CID10125426 |
| CID3469 | CID9260 | CID65015 | CID642376 | CID10158562 |
| CID3475 | CID9317 | CID65036 | CID643460 | CID10184653 |
| CID3485 | CID9385 | CID65056 | CID643975 | CID10196499 |
| CID3499 | CID9395 | CID65064 | CID644019 | CID10202471 |
| CID3501 | CID9403 | CID65064 | CID644213 | CID10322450 |
| CID3542 | CID9415 | CID65079 | CID644241 | CID10328286 |
| CID3549 | CID9415 | CID65094 | CID644280 | CID10362209 |
| CID3552 | CID9416 | CID65110 | CID644345 | CID10370280 |
| CID3559 | CID9444 | CID65126 | CID656406 | CID10382483 |
| CID3562 | CID9500 | CID65137 | CID656539 | CID10425706 |
| CID3589 | CID9502 | CID65149 | CID656583 | CID10426436 |
| CID3598 | CID9528 | CID65154 | CID656604 | CID10429470 |
| CID3599 | CID9554 | CID65237 | CID656668 | CID10442150 |
| CID3604 | CID9555 | CID65243 | CID656725 | CID10461815 |
| CID3606 | CID9642 | CID65307 | CID657237 | CID10523409 |
| CID3610 | CID9677 | CID65351 | CID657298 | CID10951331 |
| CID3616 | CID9679 | CID65359 | CID657309 | CID11105289 |
| CID3637 | CID9687 | CID65387 | CID657356 | CID11210478 |
| CID3639 | CID9756 | CID65400 | CID667476 | CID11220242 |
| CID3652 | CID9782 | CID65552 | CID667477 | CID11226684 |
| CID3657 | CID9824 | CID65569 | CID667490 | CID11228183 |
| CID3658 | CID9864 | CID65576 | CID667594 | CID11253859 |
| CID3662 | CID9903 | CID65632 | CID667639 | CID11258242 |
| CID3672 | CID9955 | CID65684 | CID680502 | CID11260998 |
| CID3676 | CID9958 | CID65884 | CID688586 | CID11267663 |
| CID3690 | CID10039 | CID65910 | CID689043 | CID11282283 |
| CID3696 | CID10112 | CID65947 | CID697993 | CID11349170 |
| CID3712 | CID10114 | CID65999 | CID699414 | CID11375554 |
| CID3715 | CID10133 | CID66011 | CID736366 | CID11375554 |
| CID3727 | CID10141 | CID66166 | CID753704 | CID11377634 |
| CID3728 | CID10168 | CID66282 | CID853433 | CID11389353 |
| CID3731 | CID10170 | CID66414 | CID854389 | CID11393311 |
| CID3758 | CID10180 | CID66420 | CID941361 | CID11396600 |
| CID3763 | CID10205 | CID66461 | CID969516 | CID11422035 |
| CID3767 | CID10206 | CID66542 | CID969516 | CID11476171 |
| CID3776 | CID10207 | CID66589 | CID1349907 | CID11487078 |
| CID3779 | CID10212 | CID67431 | CID1548887 | CID11494412 |
| CID3795 | CID10214 | CID67473 | CID1548943 | CID11524144 |
| CID3806 | CID10219 | CID67499 | CID1549008 | CID11545682 |
| CID3820 | CID10222 | CID67545 | CID1550607 | CID11556711 |
| CID3821 | CID10228 | CID67821 | CID1550884 | CID11571200 |
| CID3822 | CID10237 | CID68036 | CID1712094 | CID11578515 |
| CID3825 | CID10251 | CID68066 | CID1715135 | CID11626560 |
| CID3840 | CID10255 | CID68079 | CID1794427 | CID11644425 |
| CID3844 | CID10256 | CID68081 | CID2723631 | CID11663931 |
| CID3869 | CID10258 | CID68238 | CID2723650 | CID11683005 |
| CID3878 | CID10281 | CID68245 | CID2723949 | CID11703255 |
| CID3879 | CID10364 | CID68247 | CID2724039 | CID11706629 |
| CID3883 | CID10438 | CID68289 | CID2724126 | CID11707110 |
| CID3885 | CID10455 | CID68296 | CID2724354 | CID11720392 |
| CID3893 | CID10461 | CID68602 | CID2727594 | CID11725801 |
| CID3902 | CID10494 | CID68613 | CID2733484 | CID11749858 |
| CID3922 | CID10504 | CID68616 | CID2733526 | CID11776156 |
| CID3950 | CID10509 | CID68617 | CID2735431 | CID11843278 |
| CID3955 | CID10621 | CID68740 | CID2762596 | CID11957724 |
| CID3957 | CID10631 | CID68827 | CID2777391 | CID11960529 |
| CID3961 | CID10635 | CID68851 | CID2779853 | CID11963622 |
| CID3973 | CID10658 | CID68861 | CID2794188 | CID11966249 |
| CID4004 | CID10658 | CID68870 | CID2807869 | CID11969475 |
| CID4008 | CID10667 | CID68876 | CID2825690 | CID11969542 |
| CID4021 | CID10736 | CID68995 | CID3001055 | CID11977753 |
| CID4030 | CID10747 | CID69147 | CID3001858 | CID12047567 |
| CID4032 | CID10783 | CID69325 | CID3005532 | CID12118082 |
| CID4033 | CID10787 | CID69457 | CID3006531 | CID12134997 |
| CID4034 | CID10788 | CID69509 | CID3008868 | CID12310288 |
| CID4037 | CID10819 | CID69730 | CID3016734 | CID12310947 |
| CID4041 | CID10836 | CID69785 | CID3025899 | CID12314120 |
| CID4044 | CID10898 | CID69872 | CID3025961 | CID12855925 |
| CID4055 | CID10900 | CID70684 | CID3027782 | CID12901617 |
| CID4060 | CID10915 | CID71245 | CID3028194 | CID12968471 |
| CID4069 | CID10917 | CID71301 | CID3032285 | CID13858094 |
| CID4091 | CID10935 | CID71349 | CID3032552 | CID14104268 |
| CID4095 | CID10958 | CID71391 | CID3032581 | CID14132199 |
| CID4096 | CID10964 | CID71853 | CID3032604 | CID14217092 |
| CID4098 | CID10986 | CID71905 | CID3032791 | CID14942883 |
| CID4114 | CID11006 | CID72120 | CID3034010 | CID15251598 |
| CID4115 | CID11016 | CID72185 | CID3034285 | CID15489645 |
| CID4116 | CID11045 | CID72271 | CID3034368 | CID15509899 |
| CID4122 | CID11057 | CID72277 | CID3034683 | CID15596285 |
| CID4130 | CID11080 | CID72281 | CID3034747 | CID16038120 |
| CID4133 | CID11089 | CID72301 | CID3034818 | CID16078972 |
| CID4156 | CID11095 | CID72303 | CID3035059 | CID16129627 |
| CID4158 | CID11167 | CID72307 | CID3035200 | CID16129666 |
| CID4169 | CID11192 | CID72310 | CID3035697 | CID16129675 |
| CID4171 | CID11247 | CID72323 | CID3035711 | CID16130199 |
| CID4178 | CID11254 | CID72344 | CID3035784 | CID16130978 |
| CID4189 | CID11289 | CID72370 | CID3036904 | CID16132113 |
| CID4192 | CID11292 | CID72378 | CID3036964 | CID16132280 |
| CID4197 | CID11294 | CID72429 | CID3038525 | CID16134896 |
| CID4205 | CID11361 | CID72435 | CID3052566 | CID16137348 |
| CID4210 | CID11387 | CID72724 | CID3062316 | CID16162108 |
| CID4211 | CID11392 | CID73040 | CID3069398 | CID16211460 |
| CID4212 | CID11442 | CID73078 | CID3080871 | CID16218906 |
| CID4216 | CID11443 | CID73124 | CID3080898 | CID16219783 |
| CID4233 | CID11559 | CID73149 | CID3081047 | CID16222096 |
| CID4247 | CID11568 | CID73296 | CID3081355 | CID16394563 |
| CID4261 | CID11617 | CID73309 | CID3081361 | CID16682746 |
| CID4282 | CID11641 | CID73314 | CID3081364 | CID16682983 |
| CID4284 | CID11645 | CID73358 | CID3081884 | CID16683012 |
| CID4362 | CID11683 | CID73432 | CID3083568 | CID16684434 |
| CID4380 | CID11742 | CID73467 | CID3084025 | CID16759173 |
| CID4386 | CID11790 | CID73549 | CID3085260 | CID16759369 |
| CID4427 | CID11813 | CID73581 | CID3086599 | CID16760348 |
| CID4456 | CID11831 | CID73583 | CID3091786 | CID16760394 |
| CID4463 | CID11855 | CID73597 | CID3247059 | CID16760566 |
| CID4474 | CID11884 | CID73641 | CID3260292 | CID16760588 |
| CID4477 | CID11937 | CID73665 | CID3260293 | CID16760625 |
| CID4485 | CID11941 | CID73675 | CID3423265 | CID16760658 |
| CID4488 | CID11957 | CID73864 | CID3467590 | CID17755052 |
| CID4494 | CID12018 | CID73963 | CID3476986 | CID18477728 |
| CID4495 | CID12035 | CID73973 | CID3495594 | CID18541395 |
| CID4497 | CID12041 | CID74002 | CID3641960 | CID18982644 |
| CID4499 | CID12105 | CID74322 | CID3651377 | CID19065601 |
| CID4506 | CID12111 | CID74395 | CID3854666 | CID19362114 |
| CID4507 | CID12124 | CID74483 | CID4210951 | CID20055000 |
| CID4510 | CID12130 | CID74989 | CID4234241 | CID20749848 |
| CID4539 | CID12228 | CID75552 | CID4306515 | CID21944094 |
| CID4543 | CID12251 | CID75846 | CID4338370 | CID22152019 |
| CID4553 | CID12374 | CID75919 | CID4369270 | CID22186063 |
| CID4564 | CID12377 | CID76079 | CID4470790 | CID22273644 |
| CID4578 | CID12388 | CID76302 | CID4521392 | CID22833503 |
| CID4583 | CID12389 | CID77082 | CID4659569 | CID22833565 |
| CID4585 | CID12446 | CID77222 | CID4984721 | CID23305158 |
| CID4592 | CID12446 | CID77487 | CID4990817 | CID23557187 |
| CID4594 | CID12468 | CID77999 | CID5005498 | CID23584939 |
| CID4601 | CID12486 | CID78160 | CID5081913 | CID23634407 |
| CID4616 | CID12512 | CID78479 | CID5111791 | CID23662354 |
| CID4622 | CID12544 | CID79633 | CID5113032 | CID23663992 |
| CID4624 | CID12550 | CID81261 | CID5271566 | CID23665637 |
| CID4630 | CID12560 | CID81595 | CID5273469 | CID23667301 |
| CID4632 | CID12580 | CID82143 | CID5273569 | CID23667301 |
| CID4641 | CID12665 | CID82146 | CID5273755 | CID23668193 |
| CID4644 | CID12688 | CID82153 | CID5278396 | CID23668620 |
| CID4650 | CID12699 | CID82178 | CID5280335 | CID23673461 |
| CID4685 | CID12733 | CID82755 | CID5280343 | CID23693553 |
| CID4696 | CID12753 | CID84029 | CID5280352 | CID23725123 |
| CID4705 | CID12877 | CID84046 | CID5280360 | CID24199313 |
| CID4707 | CID12967 | CID84979 | CID5280363 | CID24204050 |
| CID4708 | CID13089 | CID85689 | CID5280373 | CID24721561 |
| CID4712 | CID13109 | CID86132 | CID5280378 | CID24772860 |
| CID4713 | CID13129 | CID86418 | CID5280389 | CID24776445 |
| CID4735 | CID13165 | CID86429 | CID5280427 | CID24799410 |
| CID4737 | CID13192 | CID86876 | CID5280435 | CID24826799 |
| CID4740 | CID13205 | CID87691 | CID5280436 | CID24838730 |
| CID4752 | CID13243 | CID88708 | CID5280443 | CID24848438 |
| CID4754 | CID13257 | CID88881 | CID5280445 | CID24848729 |
| CID4760 | CID13360 | CID89105 | CID5280448 | CID24883813 |
| CID4763 | CID13462 | CID89440 | CID5280450 | CID24884190 |
| CID4764 | CID13512 | CID89594 | CID5280453 | CID24901738 |
| CID4768 | CID13539 | CID91466 | CID5280453 | CID24978544 |
| CID4775 | CID13542 | CID91469 | CID5280457 | CID25022668 |
| CID4778 | CID13543 | CID91472 | CID5280483 | CID25033539 |
| CID4781 | CID13586 | CID91474 | CID5280483 | CID25074887 |
| CID4790 | CID13588 | CID91475 | CID5280489 | CID25078013 |
| CID4793 | CID13591 | CID91497 | CID5280489 | CID25091945 |
| CID4807 | CID13619 | CID91501 | CID5280492 | CID25092289 |
| CID4829 | CID13636 | CID91516 | CID5280493 | CID25093353 |
| CID4871 | CID13643 | CID91550 | CID5280531 | CID25108684 |
| CID4875 | CID13643 | CID91585 | CID5280598 | CID25192998 |
| CID4878 | CID13676 | CID91586 | CID5280644 | CID25197364 |
| CID4882 | CID13708 | CID91605 | CID5280754 | CID25200276 |
| CID4893 | CID13726 | CID91649 | CID5280757 | CID25254071 |
| CID4908 | CID13791 | CID91662 | CID5280794 | CID31196406 |
| CID4910 | CID14016 | CID91708 | CID5280795 | CID42608454 |
| CID4912 | CID14017 | CID91731 | CID5280805 | CID42642645 |
| CID4913 | CID14039 | CID92110 | CID5280863 | CID44134788 |
| CID4939 | CID14085 | CID92207 | CID5280883 | CID44135119 |
| CID4943 | CID14116 | CID92422 | CID5280884 | CID44145628 |
| CID4946 | CID14160 | CID92427 | CID5280899 | CID44182395 |
| CID4947 | CID14196 | CID92727 | CID5280914 | CID44251562 |
| CID4971 | CID14223 | CID92729 | CID5280933 | CID44317747 |
| CID4971 | CID14257 | CID92775 | CID5280934 | CID44429859 |
| CID4992 | CID14296 | CID92785 | CID5280951 | CID44475945 |
| CID4993 | CID14369 | CID92794 | CID5280954 | CID44575767 |
| CID5018 | CID14410 | CID92810 | CID5280961 | CID44586019 |
| CID5035 | CID14421 | CID92815 | CID5280965 | CID44586883 |
| CID5042 | CID14425 | CID93004 | CID5281004 | CID44607530 |
| CID5054 | CID14457 | CID95168 | CID5281025 | CID45115059 |
| CID5059 | CID14484 | CID95170 | CID5281033 | CID46173707 |
| CID5070 | CID14770 | CID95526 | CID5281040 | CID46839346 |
| CID5073 | CID14771 | CID95575 | CID5281051 | CID46864187 |
| CID5083 | CID14784 | CID96710 | CID5281104 | CID46926346 |
| CID5083 | CID14802 | CID97226 | CID5281126 | CID49850262 |
| CID5087 | CID14805 | CID97536 | CID5281168 | CID49868330 |
| CID5090 | CID14806 | CID97598 | CID5281222 | CID53248909 |
| CID5092 | CID14814 | CID97616 | CID5281232 | CID53308705 |
| CID5104 | CID14821 | CID98280 | CID5281303 | CID53319374 |
| CID5142 | CID14828 | CID99474 | CID5281321 | CID53398644 |
| CID5143 | CID14829 | CID99479 | CID5281416 | CID53435999 |
| CID5144 | CID14915 | CID99769 | CID5281437 | CID53477714 |
| CID5146 | CID14945 | CID99920 | CID5281544 | CID54670067 |
| CID5152 | CID14969 | CID100016 | CID5281571 | CID54671203 |
| CID5154 | CID14982 | CID100017 | CID5281576 | CID54675776 |
| CID5166 | CID14985 | CID100313 | CID5281605 | CID54675776 |
| CID5186 | CID14986 | CID100551 | CID5281605 | CID54675779 |
| CID5193 | CID15032 | CID100572 | CID5281607 | CID54675783 |
| CID5202 | CID15103 | CID101526 | CID5281613 | CID54675866 |
| CID5206 | CID15104 | CID101616 | CID5281614 | CID54676038 |
| CID5208 | CID15107 | CID102288 | CID5281616 | CID54676228 |
| CID5212 | CID15209 | CID104730 | CID5281647 | CID54676345 |
| CID5216 | CID15284 | CID104739 | CID5281650 | CID54676478 |
| CID5219 | CID15352 | CID104741 | CID5281654 | CID54676537 |
| CID5231 | CID15413 | CID104746 | CID5281656 | CID54676539 |
| CID5234 | CID15460 | CID104747 | CID5281670 | CID54676905 |
| CID5235 | CID15478 | CID104751 | CID5281672 | CID54677470 |
| CID5245 | CID15586 | CID104756 | CID5281673 | CID54678486 |
| CID5253 | CID15600 | CID104758 | CID5281701 | CID54678924 |
| CID5259 | CID15604 | CID104764 | CID5281703 | CID54679073 |
| CID5265 | CID15624 | CID104767 | CID5281707 | CID54680085 |
| CID5281 | CID15625 | CID104772 | CID5281708 | CID54680783 |
| CID5291 | CID15723 | CID104774 | CID5281718 | CID54680783 |
| CID5311 | CID15730 | CID104775 | CID5281727 | CID54682566 |
| CID5312 | CID15787 | CID104794 | CID5281728 | CID54682930 |
| CID5318 | CID15865 | CID104795 | CID5281744 | CID54684141 |
| CID5323 | CID15906 | CID104802 | CID5281771 | CID54684141 |
| CID5329 | CID15939 | CID104806 | CID5281787 | CID54698174 |
| CID5335 | CID15965 | CID104807 | CID5281793 | CID54710406 |
| CID5336 | CID15993 | CID104826 | CID5281794 | CID54740488 |
| CID5342 | CID16043 | CID104834 | CID5281800 | CID54742991 |
| CID5350 | CID16078 | CID104850 | CID5281807 | CID56593244 |
| CID5355 | CID16118 | CID104858 | CID5281847 | CID56593253 |
| CID5358 | CID16231 | CID104895 | CID5281855 | CID56840810 |
| CID5359 | CID16263 | CID104901 | CID5281872 | CID56841034 |
| CID5361 | CID16307 | CID104913 | CID5281875 | CID56841493 |
| CID5381 | CID16315 | CID104920 | CID5281881 | CID56841621 |
| CID5382 | CID16441 | CID104926 | CID5281884 | CID56841897 |
| CID5386 | CID16590 | CID104930 | CID5281887 | CID56841904 |
| CID5392 | CID16590 | CID104954 | CID5281888 | CID56841950 |
| CID5394 | CID16720 | CID104987 | CID5281894 | CID56841987 |
| CID5403 | CID16741 | CID105003 | CID5281912 | CID56841988 |
| CID5405 | CID16754 | CID105009 | CID5281915 | CID56841995 |
| CID5426 | CID16899 | CID105020 | CID5281915 | CID56842121 |
| CID5429 | CID16942 | CID105039 | CID5281929 | CID56842157 |
| CID5430 | CID16945 | CID105056 | CID5281951 | CID56842162 |
| CID5437 | CID17004 | CID105059 | CID5281969 | CID56842207 |
| CID5443 | CID17038 | CID105075 | CID5282217 | CID56842208 |
| CID5452 | CID17097 | CID107650 | CID5282227 | CID56842239 |
| CID5453 | CID17109 | CID107656 | CID5282230 | CID56928150 |
| CID5455 | CID17253 | CID107707 | CID5282253 | CID56947294 |
| CID5483 | CID17321 | CID107712 | CID5282280 | CID56947296 |
| CID5491 | CID17435 | CID107722 | CID5282347 | CID56947298 |
| CID5505 | CID17520 | CID107724 | CID5282348 | CID56947300 |
| CID5510 | CID17534 | CID107736 | CID5282350 | CID56947303 |
| CID5519 | CID17599 | CID107759 | CID5282360 | CID56973721 |
| CID5533 | CID17725 | CID107778 | CID5282379 | CID57347668 |
| CID5564 | CID17822 | CID107807 | CID5282381 | CID57367544 |
| CID5565 | CID18283 | CID107848 | CID5282443 | CID57513496 |
| CID5566 | CID18300 | CID107876 | CID5282452 | CID60156256 |
| CID5570 | CID18301 | CID107901 | CID5282455 | CID60202288 |
| CID5591 | CID18343 | CID107935 | CID5282517 | CID66557782 |
| CID5601 | CID18529 | CID107982 | CID5282796 | CID70691022 |
| CID5610 | CID18617 | CID107985 | CID5282820 | CID70695221 |
| CID5641 | CID18692 | CID108007 | CID5282948 | CID71299323 |
| CID5646 | CID18730 | CID108018 | CID5283009 | CID71300623 |
| CID5649 | CID18746 | CID108047 | CID5283218 | CID71311860 |
| CID5665 | CID19001 | CID108052 | CID5283280 | CID71312131 |
| CID5681 | CID19009 | CID108097 | CID5283344 | CID71433907 |
| CID5687 | CID19037 | CID108098 | CID5283454 | CID71587681 |
| CID5694 | CID19103 | CID108130 | CID5283560 | CID73383317 |
| CID5708 | CID19395 | CID108142 | CID5283711 | CID73755113 |
