# Supplementary material for: A computational method for the identification of candidate drugs for non-small cell lung cancer
Source: PLoS One. 2017 Aug 18;12(8):e0183411. doi: 10.1371/journal.pone.0183411 (PMC5562320; doi:10.1371/journal.pone.0183411)
Supplement: S2 Table — (DOCX) [file pone.0183411.s002.docx]

**S2 Table.** 148 genes related to NSCLC

| **Gene symbol** | **Source** |
| --- | --- |
| ALK | CTD |
| APC | CTD |
| AXL | CTD |
| BAD | KEGG |
| CAT | CTD |
| CD9 | CTD |
| EGF | KEGG |
| MET | CTD |
| MT3 | CTD |
| PXN | CTD |
| RB1 | CTD, KEGG |
| SYP | CTD |
| ABCB1 | CTD |
| ADAMTS1 | CTD |
| AKR1C1 | CTD |
| AKT1 | KEGG |
| AKT2 | KEGG |
| AKT3 | KEGG |
| ARAF | KEGG |
| AREG | CTD |
| ATF3 | CTD |
| AVEN | CTD |
| BIRC5 | CTD |
| BRAF | KEGG |
| CASP8 | CTD |
| CASP9 | KEGG |
| CCND1 | KEGG |
| CD74 | CTD |
| CDH13 | CTD |
| CDK4 | KEGG |
| CDK6 | KEGG |
| CDKN2A | CTD, KEGG |
| CHFR | CTD |
| CHGA | CTD |
| CLPTM1L | CTD |
| CORO1C | CTD |
| CSF3 | CTD |
| CST6 | CTD |
| CXCL8 | CTD |
| CYLD | CTD |
| CYP2E1 | CTD |
| DAPK1 | CTD |
| DUSP3 | CTD |
| E2F1 | KEGG |
| E2F2 | KEGG |
| E2F3 | KEGG |
| EGFR | CTD, KEGG |
| EML4 | CTD |
| ENO1 | CTD |
| ENO2 | CTD |
| ERBB2 | KEGG |
| ERCC1 | CTD |
| FASLG | CTD |
| FGF9 | CTD |
| FGFR1 | CTD |
| FHIT | CTD, KEGG |
| FOXM1 | CTD |
| FOXO3 | CTD, KEGG |
| GCLC | CTD |
| GIPR | CTD |
| GRB2 | KEGG |
| GSTM2 | CTD |
| GSTP1 | CTD |
| HES1 | CTD |
| HRAS | KEGG |
| IL10 | CTD |
| IL6R | CTD |
| ITGB1 | CTD |
| KDM1A | CTD |
| KEAP1 | CTD |
| KIF5B | CTD |
| KRAS | CTD, KEGG |
| LGALS1 | CTD |
| MAP2K1 | KEGG |
| MAP2K2 | KEGG |
| MAPK1 | KEGG |
| MAPK3 | KEGG |
| MDM2 | CTD |
| MIR1246 | CTD |
| MIR126 | CTD |
| MIR145 | CTD |
| MIR29A | CTD |
| MIR30C1 | CTD |
| MIR638 | CTD |
| MMP1 | CTD |
| MMP11 | CTD |
| MMP9 | CTD |
| MXRA5 | CTD |
| NCOA3 | CTD |
| NDRG1 | CTD |
| NFE2L2 | CTD |
| NNAT | CTD |
| NOTCH3 | CTD |
| NQO1 | CTD |
| NRAS | KEGG |
| OSMR | CTD |
| OXTR | CTD |
| PDPK1 | KEGG |
| PGAM1 | CTD |
| PHLDA2 | CTD |
| PIK3CA | KEGG |
| PIK3CB | KEGG |
| PIK3CD | KEGG |
| PIK3CG | KEGG |
| PIK3R1 | KEGG |
| PIK3R2 | KEGG |
| PIK3R3 | KEGG |
| PIK3R5 | KEGG |
| PLBD1 | CTD |
| PLCG1 | KEGG |
| PLCG2 | KEGG |
| PPIA | CTD |
| PRAME | CTD |
| PRDX1 | CTD |
| PRKCA | KEGG |
| PRKCB | KEGG |
| PRKCG | KEGG |
| PRR13 | CTD |
| PTHLH | CTD |
| PYCARD | CTD |
| RAF1 | CTD, KEGG |
| RARB | KEGG |
| RASSF1 | CTD, KEGG |
| RASSF5 | KEGG |
| RBM7 | CTD |
| RECK | CTD |
| RHEB | CTD |
| ROS1 | CTD |
| RRAD | CTD |
| RRM1 | CTD |
| RXRA | KEGG |
| RXRB | KEGG |
| RXRG | KEGG |
| SDC4 | CTD |
| SLC34A2 | CTD |
| SOD2 | CTD |
| SOS1 | KEGG |
| SOS2 | KEGG |
| STAT3 | CTD |
| STK4 | KEGG |
| TERT | CTD |
| TGFA | CTD, KEGG |
| TP53 | CTD, KEGG |
| TSG11 | CTD |
| UCHL1 | CTD |
| VEGFA | CTD |
| VIMP | CTD |
| COL4A3BP | CTD |
