# Supplementary material for: A computational method for the identification of candidate drugs for non-small cell lung cancer
Source: PLoS One. 2017 Aug 18;12(8):e0183411. doi: 10.1371/journal.pone.0183411 (PMC5562320; doi:10.1371/journal.pone.0183411)
Supplement: S3 Table — (PDF) [file pone.0183411.s003.pdf]

S3 Table. 3261 possible compounds after preliminary screening

PubChem ID	Rating score on NSCLC-related chemicals	P-value on NSCLC-related chemicals	Rating score on NSCLC-related genes	P-value on NSCLC-related genes
CID6	306.358	0.310	385.500	0.199
CID15	253.353	0.258	759.500	0.036
CID51	401.564	0.798	342.000	0.611
CID119	372.974	0.029	230.000	0.868
CID137	291.034	0.173	298.500	0.163
CID138	249.202	0.256	190.333	0.159
CID143	350.698	0.377	265.667	0.156
CID146	344.053	-	0.000	-
CID170	367.941	-	0.000	-
CID174	290.464	0.144	218.750	0.263
CID175	344.589	0.089	359.659	0.492
CID180	296.502	0.134	246.773	0.174
CID187	349.437	0.098	528.529	0.571
CID190	333.474	0.358	340.800	0.185
CID196	244.926	0.220	199.000	0.062
CID206	310.689	0.022	248.000	0.193
CID222	392.707	0.664	487.400	0.336
CID236	276.669	0.144	178.857	0.775
CID237	267.277	0.252	730.200	0.036
CID241	296.193	0.093	348.500	0.040
CID247	372.338	0.190	206.000	0.305
CID259	308.012	0.474	263.452	0.491
CID264	318.366	0.108	604.514	0.402
CID271	424.655	0.460	627.338	0.023

CID276	273.605	-	0.000	-
CID280	380.391	0.712	293.127	0.610
CID281	326.521	0.502	399.000	0.235
CID283	399.946	0.616	569.938	0.284
CID297	288.095	0.080	197.500	0.474
CID303	256.377	-	0.000	-
CID305	339.618	0.200	345.250	0.347
CID311	376.889	0.434	561.125	0.110
CID312	439.555	0.780	643.976	0.010
CID314	279.125	-	0.000	-
CID338	285.161	0.336	583.375	0.113
CID379	305.588	-	0.000	-
CID385	198.388	-	0.000	-
CID389	404.333	-	0.000	-
CID402	334.274	0.220	401.154	0.196
CID424	304.692	0.136	215.933	0.394
CID444	311.539	0.340	743.667	0.121
CID487	354.321	-	0.000	-
CID586	324.065	0.276	241.200	0.442
CID588	333.898	0.082	249.417	0.345
CID596	589.000	-	0.000	-
CID597	327.565	0.228	249.471	0.242
CID598	328.356	-	0.000	-
CID602	305.881	0.033	316.800	0.240
CID611	325.504	0.013	363.750	0.206
CID612	307.221	0.080	304.000	0.238
CID648	291.500	-	0.000	-
CID649	305.212	0.264	167.600	0.381
CID679	310.278	0.606	341.600	0.202
CID681	391.153	0.031	511.200	0.584

CID702	334.806	0.012	388.081	0.103
CID712	333.645	0.190	299.076	0.041
CID713	299.726	0.408	197.643	0.391
CID743	277.753	0.373	180.000	0.266
CID750	356.342	0.236	307.529	0.526
CID753	456.755	0.738	516.171	0.064
CID764	327.154	0.316	351.034	0.131
CID767	364.641	0.590	513.143	0.265
CID772	262.552	-	0.000	-
CID774	370.232	0.157	599.696	0.605
CID778	333.167	0.292	599.250	0.150
CID781	335.564	0.472	275.000	0.448
CID783	794.098	0.790	901.935	0.159
CID784	371.307	0.649	617.194	0.020
CID787	287.667	0.436	248.000	0.102
CID790	325.212	0.452	511.000	0.090
CID795	330.606	0.444	182.000	0.882
CID813	330.697	0.192	352.500	0.638
CID834	349.403	-	0.000	-
CID836	267.240	0.360	161.000	0.277
CID857	311.879	0.100	222.500	0.274
CID867	316.652	0.520	403.833	0.183
CID876	326.655	0.060	233.621	0.146
CID887	315.095	0.103	269.953	0.271
CID888	469.363	0.993	749.229	0.001
CID892	327.088	0.365	523.875	0.115
CID909	256.563	-	0.000	-
CID923	402.585	0.693	449.615	0.398
CID936	326.644	0.545	294.000	0.351
CID937	380.996	0.330	225.000	0.812

CID943	335.582	0.471	297.125	0.534
CID948	305.874	0.357	177.667	0.446
CID961	523.855	1.000	641.404	0.890
CID967	382.506	-	0.000	-
CID977	439.559	0.577	391.113	0.427
CID978	318.411	-	0.000	-
CID994	275.304	0.419	229.692	0.293
CID996	308.018	0.204	305.833	0.236
CID1003	399.896	0.453	495.417	0.724
CID1023	482.594	0.923	528.882	0.850
CID1032	320.422	0.123	681.222	0.253
CID1043	253.370	-	0.000	-
CID1044	332.264	0.138	157.000	0.348
CID1046	358.452	0.328	170.500	0.354
CID1048	258.319	0.193	520.500	0.054
CID1049	281.820	0.128	363.833	0.061
CID1054	336.950	-	0.000	-
CID1060	561.437	0.444	668.000	0.258
CID1099	322.612	0.398	259.750	0.209
CID1110	360.512	0.285	289.167	0.484
CID1112	469.578	0.114	187.000	0.201
CID1117	323.514	0.127	203.227	0.789
CID1125	360.691	0.533	395.000	0.228
CID1130	347.226	0.261	497.500	0.041
CID1135	333.043	0.187	245.667	0.254
CID1140	300.429	0.096	640.250	0.040
CID1153	359.118	0.292	252.000	<0.001
CID1174	348.821	0.262	197.556	0.574
CID1175	317.330	0.434	606.000	0.075
CID1188	310.168	0.359	397.833	0.132

CID1198	278.830	0.224	171.429	0.307
CID1336	203.909	-	0.000	-
CID1401	300.697	0.188	551.400	0.100
CID1405	418.750	-	0.000	-
CID1464	269.787	0.388	253.692	0.112
CID1493	248.102	0.377	169.500	0.191
CID1546	243.667	-	0.000	-
CID1567	357.991	0.516	398.346	0.265
CID1674	289.172	0.180	324.923	0.242
CID1697	173.000	0.247	461.000	0.006
CID1773	291.692	-	0.000	-
CID1775	308.954	0.256	396.556	0.210
CID1805	278.263	-	0.000	-
CID1816	309.345	-	0.000	-
CID1821	160.667	-	0.000	-
CID1923	265.765	0.190	150.000	0.240
CID1983	306.034	0.252	279.276	<0.001
CID2007	272.853	0.248	165.000	0.143
CID2016	229.175	0.343	245.000	0.055
CID2022	275.344	0.230	223.667	0.248
CID2044	249.500	0.140	475.500	0.034
CID2051	235.368	0.210	573.250	0.086
CID2088	291.522	0.303	504.000	0.113
CID2092	260.340	0.349	319.500	0.131
CID2094	272.293	0.264	486.000	0.133
CID2123	222.818	-	0.000	-
CID2134	292.875	-	0.000	-
CID2140	266.953	0.377	206.200	0.393
CID2141	278.414	0.274	466.400	0.055
CID2145	351.288	-	0.000	-

CID2148	278.875	0.341	489.000	0.093
CID2153	301.206	0.183	271.714	0.331
CID2154	384.167	-	0.000	-
CID2156	307.105	0.318	245.400	0.257
CID2160	341.995	0.224	784.500	0.203
CID2179	299.783	0.231	605.750	0.060
CID2187	285.204	0.314	239.500	0.122
CID2201	255.224	-	0.000	-
CID2202	262.568	0.438	800.000	0.012
CID2210	238.000	-	0.000	-
CID2244	318.857	0.090	417.700	0.168
CID2265	302.520	0.260	427.000	0.099
CID2266	196.449	-	0.000	-
CID2313	211.189	-	0.000	-
CID2336	308.447	0.165	296.267	<0.001
CID2349	308.180	0.088	254.200	0.136
CID2365	223.295	-	0.000	-
CID2375	310.922	0.283	448.625	0.151
CID2380	301.225	0.445	181.000	0.269
CID2459	212.591	0.375	168.500	0.173
CID2467	246.200	-	0.000	-
CID2475	235.902	0.339	161.000	0.292
CID2478	342.694	0.253	496.500	0.071
CID2514	339.197	0.687	528.604	0.050
CID2519	296.244	0.190	501.000	0.145
CID2520	306.020	0.189	466.600	0.373
CID2528	231.000	-	0.000	-
CID2538	305.455	-	0.000	-
CID2554	325.163	0.203	499.000	0.166
CID2568	199.500	-	0.000	-

CID2569	285.500	-	0.000	-
CID2577	351.615	0.130	303.000	0.021
CID2578	321.917	0.249	409.267	0.103
CID2662	291.797	0.310	535.407	0.263
CID2708	321.676	0.258	414.333	0.099
CID2719	299.629	0.261	447.778	0.211
CID2733	257.504	0.338	585.000	0.082
CID2746	364.000	0.148	188.333	0.103
CID2756	305.100	0.278	780.750	0.148
CID2764	305.654	0.350	408.667	0.113
CID2767	318.515	0.220	466.587	0.278
CID2770	284.048	0.225	800.000	<0.001
CID2776	281.421	0.149	658.250	0.037
CID2794	321.857	0.212	908.000	<0.001
CID2818	333.339	0.190	436.333	0.440
CID2907	328.268	0.168	315.323	0.227
CID2912	252.828	0.335	208.667	0.295
CID2944	286.200	-	0.000	-
CID2955	278.562	0.311	426.750	0.138
CID2969	264.269	0.099	240.000	0.368
CID3016	321.593	0.101	781.333	0.128
CID3032	304.273	0.271	458.889	0.276
CID3071	240.754	0.227	479.000	0.071
CID3082	267.809	0.275	152.000	0.197
CID3100	292.785	0.219	162.000	0.301
CID3108	286.069	0.329	792.250	0.083
CID3121	287.467	0.145	296.875	0.142
CID3152	245.526	0.369	702.333	0.067
CID3180	196.333	-	0.000	-
CID3189	278.600	-	0.000	-

CID3213	261.917	0.360	654.500	0.097
CID3228	214.750	-	0.000	-
CID3255	158.529	-	0.000	-
CID3276	341.071	-	0.000	-
CID3283	287.186	0.063	248.750	0.220
CID3300	224.286	0.244	175.000	0.035
CID3305	266.726	0.433	159.000	0.230
CID3307	208.333	0.336	168.000	0.146
CID3308	278.273	0.297	750.000	0.062
CID3324	261.296	-	0.000	-
CID3347	268.266	0.336	334.250	0.067
CID3348	275.471	0.363	778.333	0.064
CID3365	273.880	0.379	509.000	0.054
CID3366	330.140	0.237	186.000	0.127
CID3383	366.571	-	0.000	-
CID3385	327.543	0.291	541.762	0.049
CID3394	285.667	0.317	728.333	0.044
CID3397	293.664	0.289	475.000	0.151
CID3405	262.500	-	0.000	-
CID3414	298.589	0.360	239.000	0.085
CID3440	290.283	0.214	388.667	0.266
CID3446	305.147	0.225	750.000	0.060
CID3454	268.158	0.319	202.000	0.241
CID3467	310.133	0.138	322.850	0.103
CID3468	268.416	0.408	233.273	0.275
CID3485	292.314	0.192	200.938	0.394
CID3503	256.080	0.462	453.000	0.080
CID3510	205.778	-	0.000	-
CID3517	258.000	0.225	171.500	0.312
CID3589	283.922	0.242	498.500	0.057

CID3624	291.666	0.276	238.784	0.187
CID3639	313.689	-	0.000	-
CID3652	318.766	0.251	184.000	0.210
CID3657	320.241	0.212	393.778	0.108
CID3658	260.619	-	0.000	-
CID3672	273.017	0.152	403.667	0.272
CID3676	326.569	0.204	547.600	0.172
CID3690	350.052	0.317	373.250	0.131
CID3715	316.876	0.218	581.037	0.123
CID3767	285.699	0.316	467.250	0.073
CID3776	309.868	0.457	323.286	0.453
CID3825	279.850	0.346	150.000	0.467
CID3883	262.923	0.326	366.667	0.132
CID3899	349.884	0.212	565.000	0.067
CID3902	338.446	0.246	370.667	0.112
CID3922	301.303	0.223	641.000	0.057
CID3947	281.860	-	0.000	-
CID3950	339.815	0.310	334.000	0.035
CID3954	255.653	0.380	906.000	0.016
CID3958	312.766	0.257	168.000	0.202
CID3973	298.291	0.354	588.628	0.299
CID4004	292.484	0.148	177.667	0.457
CID4051	271.817	-	0.000	-
CID4053	279.000	-	0.000	-
CID4054	252.698	0.348	161.000	0.318
CID4075	263.033	0.272	503.500	0.169
CID4091	309.434	0.356	527.588	0.383
CID4095	273.608	0.289	595.500	0.107
CID4112	524.400	-	0.000	-
CID4114	259.087	0.358	185.000	0.311

CID4122	321.053	0.270	276.706	0.443
CID4139	278.620	0.366	224.667	0.295
CID4156	313.143	0.251	296.000	0.188
CID4158	310.620	-	0.000	-
CID4168	287.458	-	0.000	-
CID4169	283.422	0.277	548.667	0.041
CID4173	310.074	0.364	247.750	0.139
CID4189	291.594	0.313	852.000	0.045
CID4211	329.481	0.209	166.000	0.163
CID4212	343.892	0.182	472.429	0.189
CID4261	355.283	0.208	541.625	0.147
CID4409	361.675	-	0.000	-
CID4485	317.994	0.189	482.222	0.264
CID4495	276.931	0.270	321.000	0.189
CID4511	174.091	-	0.000	-
CID4553	332.195	0.289	530.286	0.189
CID4578	293.581	0.289	180.000	0.114
CID4594	265.615	0.260	355.500	0.196
CID4595	289.781	0.462	491.000	0.134
CID4604	352.840	0.162	207.000	0.069
CID4609	297.015	0.268	585.727	0.081
CID4610	277.538	-	0.000	-
CID4614	280.540	0.512	166.000	0.508
CID4616	271.968	0.497	260.500	0.305
CID4641	235.783	-	0.000	-
CID4649	262.522	-	0.000	-
CID4666	0.000	-	226.000	-
CID4673	321.653	0.307	409.333	0.137
CID4679	262.324	0.317	852.000	0.037
CID4705	261.303	0.164	563.875	0.100

CID4707	265.136	0.183	708.000	0.031
CID4708	263.652	0.133	663.000	0.031
CID4713	293.370	0.313	502.147	0.350
CID4735	285.714	-	0.000	-
CID4740	253.064	0.311	552.000	0.174
CID4763	306.258	0.168	582.091	0.137
CID4766	272.679	0.377	224.706	0.169
CID4781	269.972	-	0.000	-
CID4784	298.201	0.338	228.622	0.263
CID4829	306.429	0.270	575.417	0.425
CID4842	355.333	-	0.000	-
CID4873	325.646	0.038	259.829	0.249
CID4909	306.177	0.330	244.000	0.194
CID4911	264.389	0.318	510.500	0.229
CID4913	333.205	0.274	206.000	0.220
CID4915	363.500	0.236	247.333	0.068
CID4917	282.832	-	0.000	-
CID4939	287.644	0.362	272.564	0.144
CID4946	343.336	0.072	472.625	0.252
CID4971	347.889	0.326	437.000	0.115
CID4973	463.655	0.253	809.500	0.287
CID4993	314.976	0.411	636.800	0.074
CID4996	378.167	0.140	163.000	0.138
CID5035	320.887	0.332	410.048	0.193
CID5070	245.032	0.370	598.000	0.145
CID5090	285.919	0.293	300.600	0.162
CID5198	327.750	-	0.000	-
CID5202	387.137	0.054	572.500	0.581
CID5210	259.486	-	0.000	-
CID5213	238.459	0.268	826.111	0.038

CID5215	313.325	0.289	811.000	<0.001
CID5234	340.201	<0.001	259.243	0.183
CID5235	255.372	0.360	321.650	0.076
CID5245	306.562	0.291	527.000	0.061
CID5291	308.654	0.305	417.059	0.385
CID5311	324.202	0.198	458.407	0.117
CID5315	267.278	-	0.000	-
CID5329	307.810	-	0.000	-
CID5333	276.211	0.312	376.000	0.133
CID5336	368.179	-	0.000	-
CID5342	269.862	0.227	831.000	0.038
CID5344	283.276	0.314	161.000	0.289
CID5345	295.828	0.295	533.500	0.060
CID5360	251.845	0.523	569.556	0.251
CID5381	348.818	0.145	680.000	0.084
CID5386	288.027	-	0.000	-
CID5394	277.094	0.393	318.684	0.226
CID5413	290.799	0.316	241.000	0.340
CID5426	299.323	0.303	464.053	0.274
CID5453	319.816	0.218	352.750	0.046
CID5455	260.069	0.494	719.000	0.024
CID5472	255.990	0.386	819.000	0.085
CID5508	328.083	-	0.000	-
CID5546	250.221	0.408	279.000	0.178
CID5561	265.000	-	0.000	-
CID5578	291.426	0.330	313.000	0.098
CID5582	307.774	-	0.000	-
CID5585	274.100	-	0.000	-
CID5590	309.873	0.149	263.065	0.091
CID5641	278.162	0.250	467.250	0.064

CID5706	223.444	-	0.000	-
CID5724	241.000	-	0.000	-
CID5743	318.873	0.096	558.737	0.325
CID5744	200.916	0.333	171.500	0.177
CID5746	313.781	0.169	439.167	0.149
CID5754	311.072	0.072	399.273	0.544
CID5755	289.768	0.130	327.800	0.245
CID5757	333.304	0.096	532.537	<0.001
CID5760	219.881	0.247	169.500	0.273
CID5768	301.043	0.224	660.333	0.071
CID5770	284.394	0.176	418.667	0.213
CID5778	571.714	-	0.000	-
CID5789	310.473	0.043	258.500	0.347
CID5790	280.625	0.179	204.000	0.210
CID5793	359.467	0.001	317.788	0.050
CID5798	294.149	0.166	909.000	<0.001
CID5799	257.484	-	0.000	-
CID5802	292.808	-	0.000	-
CID5815	396.278	0.129	670.077	0.537
CID5819	311.962	0.438	468.333	0.211
CID5825	309.393	0.131	164.000	0.277
CID5833	304.884	0.332	473.600	0.230
CID5834	205.029	0.407	800.000	<0.001
CID5852	282.957	0.214	166.000	0.300
CID5865	300.091	0.154	299.143	0.221
CID5877	195.833	0.696	237.333	0.091
CID5884	891.828	0.564	918.000	0.107
CID5885	585.129	0.906	566.100	0.610
CID5897	281.979	0.230	537.000	0.037
CID5901	228.231	-	0.000	-

CID5904	303.229	0.299	409.000	0.162
CID5905	224.300	0.278	151.000	0.282
CID5920	318.995	0.402	441.417	0.249
CID5937	240.889	-	0.000	-
CID5954	326.898	0.165	275.200	0.057
CID5955	258.239	0.336	444.400	0.005
CID5957	418.866	0.648	768.188	0.034
CID5959	320.743	0.191	295.600	0.122
CID5960	417.254	0.254	208.000	0.772
CID5961	341.659	0.267	289.702	0.527
CID5962	354.362	0.148	381.030	0.420
CID5978	303.750	0.216	819.000	<0.001
CID5984	300.022	0.072	286.600	0.253
CID5988	337.216	0.019	239.657	0.512
CID5994	320.423	0.063	529.400	0.011
CID5997	353.848	0.007	329.485	0.522
CID6006	336.745	0.321	196.222	0.253
CID6011	168.375	-	0.000	-
CID6013	314.375	0.096	357.484	0.121
CID6021	370.000	0.358	151.000	0.616
CID6022	673.775	0.613	884.734	0.020
CID6029	314.565	0.106	176.200	0.765
CID6030	376.353	0.286	428.000	0.422
CID6035	278.650	0.249	234.864	0.271
CID6037	311.114	0.348	286.650	0.307
CID6047	344.549	0.321	374.800	0.272
CID6049	337.030	0.006	274.413	0.151
CID6058	299.953	0.237	341.000	0.144
CID6061	225.846	-	0.000	-
CID6076	324.099	0.030	487.591	0.257

CID6083	461.227	0.622	628.441	0.288
CID6104	277.393	0.244	180.000	0.029
CID6113	273.540	-	0.000	-
CID6115	282.312	0.123	534.000	0.028
CID6163	315.111	0.097	150.000	0.061
CID6167	280.258	0.536	500.333	0.181
CID6175	357.866	0.218	172.667	0.571
CID6194	232.625	-	0.000	-
CID6197	305.042	0.177	441.861	0.219
CID6199	279.414	0.330	205.000	0.216
CID6212	296.961	0.119	256.848	0.133
CID6224	293.421	0.106	209.550	0.384
CID6235	253.318	-	0.000	-
CID6249	321.416	0.130	244.667	0.259
CID6251	294.536	0.338	397.500	0.167
CID6252	320.799	0.178	257.111	0.169
CID6256	258.393	0.305	522.667	0.048
CID6262	376.851	0.240	649.500	0.366
CID6274	320.461	0.247	270.833	0.538
CID6279	299.076	0.143	442.600	0.087
CID6287	341.496	0.089	196.000	0.626
CID6288	362.287	0.265	180.000	0.615
CID6306	322.579	0.198	180.800	0.837
CID6322	353.323	0.199	381.968	0.490
CID6325	293.313	0.231	288.750	0.085
CID6329	288.082	0.408	175.000	0.434
CID6337	243.490	-	0.000	-
CID6338	274.964	0.278	886.667	0.006
CID6342	270.032	0.321	194.727	0.485
CID6363	303.218	-	0.000	-

CID6421	294.067	0.229	255.000	0.203
CID6436	322.987	0.213	407.000	0.027
CID6446	173.698	-	0.000	-
CID6468	277.862	0.227	158.800	0.427
CID6497	290.258	-	0.000	-
CID6503	349.595	0.020	300.659	0.119
CID6508	276.395	0.264	176.000	0.180
CID6579	286.215	0.299	268.300	0.191
CID6674	221.826	-	0.000	-
CID6675	333.714	0.356	313.667	0.228
CID6693	228.061	-	0.000	-
CID6694	292.044	0.313	212.579	0.293
CID6713	355.714	-	0.000	-
CID6741	276.498	0.206	618.400	0.045
CID6742	164.429	0.345	170.429	0.086
CID6780	264.980	-	0.000	-
CID6802	308.678	0.332	397.125	0.103
CID6811	255.939	0.608	238.400	0.277
CID6830	402.076	0.359	671.537	0.302
CID6968	255.200	-	0.000	-
CID6994	199.125	-	0.000	-
CID7099	243.250	0.363	181.000	0.046
CID7187	257.913	0.110	909.000	0.011
CID7207	208.182	-	0.000	-
CID7272	260.471	-	0.000	-
CID7339	577.349	-	0.000	-
CID7570	274.317	-	0.000	-
CID7639	260.167	-	0.000	-
CID7658	0.000	-	0.000	-
CID7767	162.667	-	0.000	-

CID7847	278.169	0.188	550.000	0.264
CID7870	203.333	-	0.000	-
CID7901	278.444	-	0.000	-
CID7909	222.817	0.511	257.000	0.104
CID7912	140.600	0.659	156.000	0.356
CID7950	224.158	0.303	288.000	<0.001
CID7965	197.105	-	0.000	-
CID8019	286.905	-	0.000	-
CID8021	221.281	-	0.000	-
CID8027	275.878	-	0.000	-
CID8198	213.333	-	0.000	-
CID8323	214.271	-	0.000	-
CID8378	286.779	0.176	295.565	0.175
CID8471	256.350	-	0.000	-
CID8505	265.919	0.384	176.000	0.399
CID8549	275.004	0.440	434.571	0.136
CID8582	504.742	-	0.000	-
CID8583	266.674	0.352	173.500	0.358
CID8589	195.000	-	0.000	-
CID8642	324.355	-	0.000	-
CID8646	293.431	0.286	152.000	0.304
CID8686	128.000	-	0.000	-
CID8701	223.896	-	0.000	-
CID8706	307.800	-	0.000	-
CID8769	143.333	-	0.000	-
CID8778	305.049	0.141	248.000	0.193
CID8971	230.737	-	0.000	-
CID8977	773.765	0.347	873.000	0.137
CID8987	270.324	0.242	540.000	0.074
CID9033	273.761	0.236	296.500	0.056

CID9048	398.241	0.214	259.000	0.085
CID9050	163.525	-	0.000	-
CID9053	182.577	-	0.000	-
CID9059	201.000	-	0.000	-
CID9062	256.322	0.297	520.500	0.111
CID9210	247.075	0.475	320.400	0.058
CID9215	237.239	0.390	203.500	0.047
CID9222	247.071	-	0.000	-
CID9250	293.565	0.233	458.000	0.046
CID9260	286.730	0.189	247.857	0.127
CID9334	169.333	-	0.000	-
CID9427	332.263	-	0.000	-
CID9444	292.630	0.385	400.391	0.284
CID9533	253.357	-	0.000	-
CID9559	228.591	-	0.000	-
CID9570	215.571	-	0.000	-
CID9571	189.000	-	0.000	-
CID9642	237.391	-	0.000	-
CID9651	260.704	-	0.000	-
CID9679	282.741	0.339	197.000	0.250
CID9700	363.202	0.184	171.000	0.540
CID9750	311.769	0.611	384.500	0.226
CID9782	263.130	0.262	380.000	0.120
CID9864	285.337	0.254	822.000	0.008
CID10001	0.000	-	0.000	-
CID10258	240.554	0.556	800.000	0.035
CID10340	301.277	0.099	272.429	0.134
CID10413	290.507	0.091	180.000	0.261
CID10423	178.182	-	0.000	-
CID10428	281.600	-	0.000	-

CID10430	266.242	0.125	161.000	0.172
CID10439	207.903	0.482	164.000	0.271
CID10457	261.125	0.166	169.000	0.100
CID10461	250.653	0.356	484.400	0.333
CID10465	313.863	0.023	551.000	<0.001
CID10466	179.238	-	0.000	-
CID10467	292.790	-	0.000	-
CID10531	249.398	0.481	800.000	0.043
CID10607	289.378	-	0.000	-
CID10635	313.634	0.122	419.875	0.183
CID10713	224.286	-	0.000	-
CID10786	271.450	0.104	179.667	0.240
CID10819	263.100	-	0.000	-
CID10848	322.167	-	0.000	-
CID10898	273.875	-	0.000	-
CID10964	283.525	0.224	290.000	0.178
CID11005	370.101	0.016	204.200	0.584
CID11016	354.500	-	0.000	-
CID11103	309.920	0.225	224.333	0.029
CID11108	225.444	-	0.000	-
CID11177	424.935	-	0.000	-
CID11178	84.600	0.320	187.000	0.003
CID11254	329.250	0.205	493.600	0.076
CID11266	286.151	0.152	196.000	0.089
CID11337	186.000	-	0.000	-
CID11339	211.979	0.619	169.000	0.235
CID11347	194.429	-	0.000	-
CID11558	185.000	-	0.000	-
CID11583	275.240	-	0.000	-
CID11668	275.871	0.373	161.000	0.435

CID11683	240.680	0.297	163.000	0.302
CID11684	197.333	-	0.000	-
CID11848	174.000	-	0.000	-
CID11870	243.429	-	0.000	-
CID11979	215.188	0.502	229.750	0.288
CID12021	203.143	-	0.000	-
CID12035	270.611	0.284	393.333	0.306
CID12137	224.485	0.308	178.750	0.152
CID12219	232.500	-	0.000	-
CID12392	233.000	-	0.000	-
CID12560	299.599	0.195	533.000	0.047
CID12587	210.852	-	0.000	-
CID12660	265.855	0.285	192.667	0.146
CID12681	26.000	-	0.000	-
CID12699	279.068	0.199	313.167	0.091
CID12729	205.750	-	0.000	-
CID12733	284.765	0.138	381.000	0.123
CID12736	200.875	0.299	186.000	0.038
CID12836	258.533	-	0.000	-
CID12967	256.846	0.297	286.400	0.076
CID13116	281.200	0.240	409.000	0.028
CID13136	231.481	-	0.000	-
CID13201	282.500	-	0.000	-
CID13342	296.786	0.311	437.000	0.161
CID13344	237.630	-	0.000	-
CID13369	205.286	-	0.000	-
CID13588	264.302	0.200	264.857	0.093
CID13698	294.172	0.313	220.476	0.161
CID13711	334.768	0.235	262.313	0.234
CID13712	308.228	0.330	192.667	0.392

CID13730	293.046	-	0.000	-
CID13849	420.724	0.015	856.857	0.533
CID13945	386.333	0.275	924.000	0.032
CID14051	293.264	0.335	197.200	0.346
CID14129	198.063	-	0.000	-
CID14227	238.568	0.450	230.000	0.141
CID14457	253.589	0.093	179.000	0.131
CID14724	185.683	-	0.000	-
CID14797	289.761	0.112	225.333	0.192
CID14798	301.206	0.033	216.217	0.352
CID14888	266.058	0.210	419.790	0.005
CID14932	208.300	0.389	237.000	0.032
CID14941	245.274	0.370	231.000	0.097
CID15032	306.902	0.194	209.000	0.181
CID15184	251.375	-	0.000	-
CID15531	284.645	-	0.000	-
CID15628	173.667	-	0.000	-
CID15702	239.167	-	0.000	-
CID15979	259.864	-	0.000	-
CID15993	334.308	0.264	312.250	0.340
CID16368	0.000	-	0.000	-
CID16456	290.000	-	0.000	-
CID16552	204.750	-	0.000	-
CID16574	227.000	-	0.000	-
CID16834	245.911	0.260	592.667	0.050
CID16850	279.125	0.326	229.000	0.279
CID16923	217.735	-	0.000	-
CID16938	209.080	-	0.000	-
CID17513	287.600	0.236	199.000	0.146
CID17648	454.875	-	0.000	-

CID17649	259.909	-	0.000	-
CID18343	323.970	0.213	605.800	0.238
CID18397	414.000	-	0.000	-
CID18407	72.125	0.390	221.000	0.028
CID18730	281.608	0.342	186.792	0.550
CID18757	237.571	-	0.000	-
CID18771	230.600	-	0.000	-
CID19001	273.818	0.348	184.071	0.187
CID19079	247.571	-	0.000	-
CID19128	224.600	-	0.000	-
CID19161	197.000	-	0.000	-
CID19261	185.853	0.229	150.000	0.028
CID19494	106.667	-	0.000	-
CID19702	207.714	-	0.000	-
CID19731	290.524	0.293	200.588	0.487
CID19888	0.000	-	0.000	-
CID20188	206.500	-	0.000	-
CID20193	252.800	-	0.000	-
CID20279	290.897	0.212	614.667	0.115
CID20469	372.286	0.174	516.667	0.114
CID20984	39.333	0.481	459.000	0.006
CID21157	271.133	0.289	506.067	0.130
CID21319	314.675	-	0.000	-
CID21363	126.167	0.782	178.000	0.019
CID21649	230.857	-	0.000	-
CID21672	182.588	0.385	190.000	0.011
CID21704	241.347	0.275	203.000	0.087
CID21765	299.800	-	0.000	-
CID21826	288.800	-	0.000	-
CID22128	234.111	-	0.000	-

CID22230	168.200	-	0.000	-
CID22318	294.118	0.258	683.500	0.093
CID22469	304.596	0.237	811.000	<0.001
CID22986	205.000	0.108	156.000	<0.001
CID23267	265.500	0.185	178.500	0.203
CID23333	170.400	-	0.000	-
CID23424	264.409	0.173	180.000	0.019
CID23723	236.808	0.454	197.000	0.299
CID23830	332.044	0.297	314.451	0.186
CID23930	389.422	0.910	605.842	0.041
CID23934	231.000	-	0.000	-
CID23939	302.862	0.321	316.667	0.238
CID23957	227.640	0.784	582.000	0.008
CID23963	278.286	0.297	508.000	0.051
CID23968	270.636	0.384	204.727	0.243
CID23978	358.516	0.326	387.261	0.330
CID23982	286.709	0.515	168.500	0.665
CID23991	320.708	0.388	527.000	0.096
CID23993	268.211	-	0.000	-
CID23994	444.015	0.995	556.986	0.007
CID24066	350.957	-	0.000	-
CID24083	300.596	0.170	262.000	0.172
CID24203	300.701	0.133	208.955	0.449
CID24247	283.205	0.316	193.000	0.319
CID24261	290.960	0.097	373.615	0.170
CID24293	317.624	0.369	179.889	0.768
CID24360	288.281	0.342	446.250	0.168
CID24441	302.204	0.253	233.550	0.222
CID24450	281.876	0.256	306.600	0.051
CID24466	373.667	-	0.000	-

CID24524	281.056	0.347	207.385	0.357
CID24529	312.844	0.455	320.375	0.124
CID24538	304.736	0.153	254.667	0.180
CID24584	315.423	0.055	239.741	0.121
CID24632	287.889	0.229	295.000	0.099
CID24719	321.122	-	0.000	-
CID24748	239.429	0.107	199.000	0.136
CID24759	254.264	0.229	361.625	0.187
CID24763	278.561	0.244	326.000	0.050
CID24766	292.198	0.367	211.000	0.603
CID24769	283.376	0.318	188.500	0.233
CID24775	311.700	0.246	245.680	0.148
CID24777	271.727	-	0.000	-
CID24841	321.207	0.582	327.895	0.568
CID24854	320.559	0.066	236.792	0.194
CID24855	251.037	-	0.000	-
CID25306	362.500	-	0.000	-
CID25419	264.000	0.311	298.800	0.049
CID25447	386.538	-	0.000	-
CID25473	244.302	0.243	700.000	<0.001
CID25572	291.541	-	0.000	-
CID25774	258.000	-	0.000	-
CID26031	317.250	-	0.000	-
CID26105	310.606	0.382	167.000	0.026
CID26650	276.881	0.634	160.000	0.653
CID26819	233.000	-	0.000	-
CID26879	275.403	0.434	196.800	0.272
CID26986	283.412	-	0.000	-
CID27012	215.400	-	0.000	-
CID27287	343.281	0.154	180.000	0.229

CID27337	248.294	0.266	413.000	0.018
CID28240	258.556	-	0.000	-
CID28280	307.000	-	0.000	-
CID28417	307.155	0.286	221.000	0.315
CID28486	307.187	0.186	572.375	0.096
CID28694	278.500	-	0.000	-
CID28780	245.872	0.272	184.000	0.398
CID29029	358.000	0.254	434.000	0.017
CID29142	253.600	-	0.000	-
CID29243	271.167	-	0.000	-
CID29327	280.048	0.297	483.267	0.151
CID29335	345.474	-	0.000	-
CID29336	204.000	-	0.000	-
CID29393	242.867	-	0.000	-
CID30323	311.747	0.158	391.273	0.266
CID30623	282.760	0.267	150.000	0.109
CID30751	225.277	0.293	800.000	0.013
CID30819	467.028	0.652	575.944	0.299
CID30975	304.811	-	0.000	-
CID31083	289.389	0.119	183.000	0.158
CID31254	253.118	0.132	700.000	<0.001
CID31261	240.213	-	0.000	-
CID31307	309.679	0.235	512.000	0.087
CID31401	283.623	0.254	657.400	0.159
CID31402	323.372	0.196	724.000	0.014
CID31515	293.529	-	0.000	-
CID31586	204.462	-	0.000	-
CID31694	0.000	-	0.000	-
CID31703	317.730	0.104	569.902	0.136
CID31789	245.267	-	0.000	-

CID31937	236.500	-	0.000	-
CID32798	285.548	-	0.000	-
CID32874	275.593	0.185	181.000	<0.001
CID33032	422.269	0.746	786.600	0.287
CID33039	218.571	0.497	163.000	0.035
CID33113	409.573	0.437	729.364	0.025
CID33557	280.844	0.340	240.000	0.229
CID33576	288.586	0.230	244.500	0.203
CID33613	331.319	0.261	525.500	0.033
CID33641	355.857	0.087	700.000	<0.001
CID33676	287.889	-	0.000	-
CID33741	301.136	-	0.000	-
CID33776	285.886	0.257	491.750	0.019
CID34192	208.037	0.411	192.000	0.018
CID34457	327.727	0.127	237.000	0.005
CID34755	532.860	0.346	384.000	0.640
CID35370	267.172	0.278	439.800	0.170
CID35595	241.200	-	0.000	-
CID36294	317.234	0.311	221.333	0.149
CID36462	323.811	0.169	554.395	0.171
CID36603	254.054	0.540	260.000	0.110
CID36646	170.000	-	0.000	-
CID36797	219.188	0.403	190.000	0.057
CID37542	258.397	0.448	379.500	0.183
CID37625	170.667	-	0.000	-
CID37768	337.519	0.280	230.000	0.122
CID38003	264.667	0.198	181.500	0.197
CID38347	322.628	0.238	295.600	0.069
CID38777	366.850	0.379	282.000	0.032
CID38852	278.991	0.217	253.500	0.130

CID39186	291.861	0.322	882.500	0.034
CID39214	330.649	0.273	150.000	0.054
CID39562	276.310	0.214	706.500	0.020
CID39981	351.800	0.180	161.000	0.090
CID40291	217.333	-	0.000	-
CID40772	234.000	0.239	204.000	0.224
CID40839	365.093	0.207	186.667	0.071
CID41526	295.143	-	0.000	-
CID41867	342.417	0.219	428.357	0.121
CID41945	212.778	-	0.000	-
CID42616	298.763	0.168	970.000	<0.001
CID42640	260.500	-	0.000	-
CID42890	352.155	0.199	296.000	0.094
CID43157	319.722	-	0.000	-
CID43805	453.353	0.198	800.000	0.024
CID43806	186.818	-	0.000	-
CID44093	319.786	0.231	491.167	0.176
CID44567	393.750	-	0.000	-
CID46151	479.000	-	0.000	-
CID46855	262.667	-	0.000	-
CID47073	306.917	-	0.000	-
CID47326	262.620	0.235	182.000	0.077
CID47938	209.077	0.189	268.000	0.006
CID49561	220.688	0.431	173.000	0.040
CID50515	242.167	0.239	469.000	<0.001
CID50599	308.685	0.233	194.000	0.113
CID50981	255.118	0.303	209.000	0.043
CID52999	227.821	0.434	161.000	0.347
CID53232	265.680	0.351	661.435	0.192
CID54048	261.000	-	0.000	-

CID54217	264.157	-	0.000	-
CID54368	404.667	-	0.000	-
CID54369	278.786	-	0.000	-
CID54454	285.955	0.303	620.219	0.172
CID54575	282.586	-	0.000	-
CID54586	295.286	-	0.000	-
CID54687	295.845	0.309	806.250	0.145
CID54744	216.615	-	0.000	-
CID54786	212.500	-	0.000	-
CID54840	259.822	-	0.000	-
CID54886	232.261	0.443	179.000	0.133
CID54888	373.000	-	0.000	-
CID54949	179.000	-	0.000	-
CID55245	314.255	0.269	424.409	0.281
CID55283	297.946	0.336	871.667	0.009
CID55361	256.200	-	0.000	-
CID55466	290.937	0.377	239.000	0.044
CID55498	484.500	-	0.000	-
CID55748	247.222	-	0.000	-
CID57009	0.000	-	0.000	-
CID57469	317.326	0.265	700.000	0.107
CID59226	246.167	-	0.000	-
CID60172	346.385	-	0.000	-
CID60198	264.094	0.242	376.750	0.064
CID60407	272.200	-	0.000	-
CID60417	393.857	-	0.000	-
CID60496	246.921	-	0.000	-
CID60505	247.500	-	0.000	-
CID60606	283.597	0.419	617.500	0.092
CID60613	294.205	0.381	194.000	0.077

CID60668	206.200	-	0.000	-
CID60699	303.375	0.343	731.222	0.065
CID60749	320.739	0.228	499.838	0.126
CID60751	334.143	-	0.000	-
CID60772	234.709	-	0.000	-
CID60779	306.804	0.317	291.867	0.069
CID60795	318.933	0.170	344.000	0.246
CID60808	268.429	0.204	800.000	<0.001
CID60822	286.810	0.343	562.444	0.364
CID60825	330.228	0.315	408.333	0.128
CID60830	271.528	-	0.000	-
CID60834	295.071	-	0.000	-
CID60837	309.574	0.310	439.694	0.244
CID60852	307.756	0.246	262.000	0.121
CID60855	231.400	-	0.000	-
CID60863	264.200	-	0.000	-
CID60871	261.424	-	0.000	-
CID60877	365.314	-	0.000	-
CID60934	300.701	0.306	836.500	0.026
CID60953	312.059	0.302	370.500	0.265
CID60955	225.632	0.272	230.000	0.025
CID60961	354.496	0.023	507.792	0.447
CID61038	199.818	-	0.000	-
CID61164	195.333	-	0.000	-
CID61336	238.351	-	0.000	-
CID61475	221.524	-	0.000	-
CID61492	203.367	-	0.000	-
CID61565	309.000	0.081	200.500	0.015
CID61635	229.955	0.332	299.750	0.063
CID61671	311.780	0.332	259.634	0.235

CID62110	52.600	-	0.000	-
CID62210	231.867	0.193	150.000	0.111
CID62369	204.833	-	0.000	-
CID62432	227.250	-	0.000	-
CID62673	224.600	-	0.000	-
CID63022	188.067	-	0.000	-
CID64142	298.541	0.291	699.200	0.146
CID64147	244.108	-	0.000	-
CID64376	186.000	-	0.000	-
CID64627	204.667	-	0.000	-
CID64730	250.123	0.083	192.000	0.146
CID64780	228.556	-	0.000	-
CID64840	343.000	-	0.000	-
CID64968	480.231	0.128	904.500	0.050
CID65005	239.206	0.282	187.000	0.024
CID65014	219.717	0.506	207.000	0.203
CID65028	221.487	-	0.000	-
CID65041	293.094	0.167	190.500	0.259
CID65046	289.303	0.405	172.929	0.479
CID65053	244.579	-	0.000	-
CID65063	483.457	0.375	274.000	0.221
CID65064	284.911	0.224	550.579	0.162
CID65087	198.200	-	0.000	-
CID65091	384.511	0.407	921.000	0.019
CID65106	266.353	0.431	179.000	0.029
CID65110	309.773	0.428	347.333	0.159
CID65138	427.500	-	0.000	-
CID65185	279.537	0.414	180.600	0.529
CID65191	230.931	0.409	173.000	0.072
CID65217	278.847	0.312	334.200	0.017

CID65237	295.806	0.233	162.000	0.037
CID65253	279.917	0.461	182.000	0.143
CID65260	323.800	-	0.000	-
CID65275	299.222	0.306	242.909	0.109
CID65366	205.429	-	0.000	-
CID65407	336.140	0.221	207.000	0.259
CID65430	218.114	-	0.000	-
CID65457	257.800	-	0.000	-
CID65577	258.075	0.253	165.667	0.308
CID65582	241.167	-	0.000	-
CID65600	215.333	-	0.000	-
CID65628	322.027	0.251	536.200	0.049
CID65702	262.000	-	0.000	-
CID65948	259.542	0.141	800.000	<0.001
CID65958	230.623	0.430	383.333	0.045
CID66031	79.000	-	0.000	-
CID66249	284.182	-	0.000	-
CID66328	190.000	-	0.000	-
CID66373	234.000	-	0.000	-
CID66377	228.248	0.414	188.400	0.382
CID66868	305.831	0.305	360.000	0.117
CID66898	326.700	-	0.000	-
CID67105	197.000	-	0.000	-
CID67431	283.667	0.187	161.000	0.260
CID67469	171.000	-	0.000	-
CID67491	287.417	0.213	196.000	0.012
CID67538	279.438	-	0.000	-
CID67574	185.000	-	0.000	-
CID67973	345.909	-	0.000	-
CID68152	283.320	0.255	238.318	0.197

CID68172	226.500	-	0.000	-
CID68216	213.000	-	0.000	-
CID68271	293.875	-	0.000	-
CID68329	196.000	0.374	258.000	0.024
CID68368	305.890	0.164	161.000	0.425
CID68691	185.500	-	0.000	-
CID68737	185.000	-	0.000	-
CID68740	321.326	0.329	392.167	0.076
CID68770	234.154	0.263	937.000	<0.001
CID68888	187.000	-	0.000	-
CID69190	317.667	-	0.000	-
CID69191	187.000	-	0.000	-
CID69435	236.926	0.325	900.000	<0.001
CID69785	484.308	-	0.000	-
CID70186	231.760	-	0.000	-
CID70347	178.000	-	0.000	-
CID71017	146.000	-	0.000	-
CID71068	264.869	0.397	184.625	0.227
CID71184	259.852	0.389	264.000	0.029
CID71237	213.438	0.478	161.000	0.035
CID71352	234.824	-	0.000	-
CID71384	295.200	-	0.000	-
CID71398	148.500	0.176	183.000	0.124
CID71406	188.067	0.484	846.000	<0.001
CID71474	154.381	-	0.000	-
CID71563	277.077	0.231	900.000	<0.001
CID71601	225.911	-	0.000	-
CID71606	63.727	-	0.000	-
CID71614	192.333	-	0.000	-
CID71616	249.340	0.391	166.000	0.263

CID71655	224.947	-	0.000	-
CID71682	213.068	0.525	162.000	0.116
CID71734	306.429	-	0.000	-
CID71741	312.227	0.249	180.000	<0.001
CID71993	362.000	-	0.000	-
CID72031	266.200	-	0.000	-
CID72120	310.000	-	0.000	-
CID72231	414.250	-	0.000	-
CID72271	278.607	0.227	452.810	0.211
CID72398	302.091	-	0.000	-
CID72402	281.895	0.401	160.500	0.090
CID72403	327.000	-	0.000	-
CID72422	301.000	-	0.000	-
CID72423	474.857	-	0.000	-
CID72435	252.333	0.266	345.667	0.028
CID72438	390.250	-	0.000	-
CID72439	0.000	-	0.000	-
CID72440	179.667	-	0.000	-
CID72441	196.000	-	0.000	-
CID72466	0.000	-	0.000	-
CID72495	217.167	-	0.000	-
CID72512	287.667	-	0.000	-
CID72564	230.000	-	0.000	-
CID72571	239.667	0.221	158.000	0.017
CID72675	354.750	-	0.000	-
CID72828	266.700	-	0.000	-
CID72991	257.600	-	0.000	-
CID73000	291.035	-	0.000	-
CID73009	222.143	0.252	196.000	0.029
CID73115	223.000	-	0.000	-

CID73212	275.718	0.249	343.913	0.016
CID73240	284.500	-	0.000	-
CID73374	363.000	-	0.000	-
CID73577	231.667	-	0.000	-
CID73627	364.333	-	0.000	-
CID74600	205.000	-	0.000	-
CID74946	221.000	-	0.000	-
CID74989	287.160	0.438	227.000	0.193
CID74999	193.000	-	0.000	-
CID75142	271.649	0.321	307.957	0.171
CID75394	339.261	-	0.000	-
CID75444	136.000	-	0.000	-
CID75928	170.667	-	0.000	-
CID77341	228.871	-	0.000	-
CID77466	278.088	0.539	161.000	0.490
CID77990	269.937	-	0.000	-
CID77998	348.988	0.319	636.725	0.427
CID78061	65.000	-	0.000	-
CID78165	447.611	0.641	636.571	0.058
CID78896	0.000	-	0.000	-
CID79034	267.758	-	0.000	-
CID80078	223.000	-	0.000	-
CID80170	258.471	0.342	158.000	0.154
CID81485	273.500	-	0.000	-
CID81531	249.310	-	0.000	-
CID82146	277.288	0.227	591.615	0.129
CID83030	347.111	-	0.000	-
CID83695	278.714	0.293	196.125	0.371
CID83821	163.667	-	0.000	-
CID83869	247.172	-	0.000	-

CID83970	279.647	0.221	181.000	0.025
CID84029	290.380	0.262	279.000	0.144
CID84078	381.667	-	0.000	-
CID84691	252.125	0.320	336.000	0.050
CID84759	290.444	0.247	182.000	<0.001
CID84867	228.667	-	0.000	-
CID86222	218.400	0.247	181.000	0.016
CID89105	385.576	0.181	930.000	0.010
CID89594	313.525	0.316	496.472	0.200
CID90235	152.833	-	0.000	-
CID90819	326.000	-	0.000	-
CID91271	298.000	-	0.000	-
CID91438	178.810	-	0.000	-
CID91443	497.522	-	0.000	-
CID91482	190.208	0.349	159.000	0.265
CID91683	253.444	-	0.000	-
CID91687	275.416	0.640	209.000	0.263
CID91741	245.083	-	0.000	-
CID91750	216.381	-	0.000	-
CID92242	299.286	0.282	170.333	0.041
CID92312	201.667	-	0.000	-
CID92368	254.250	-	0.000	-
CID92556	0.000	-	0.000	-
CID92727	290.460	0.307	307.833	0.109
CID92787	324.221	0.229	414.000	0.075
CID92929	298.500	-	0.000	-
CID93004	301.000	0.096	545.571	0.058
CID93542	210.583	-	0.000	-
CID93577	253.958	0.147	191.000	0.058
CID93860	272.500	0.104	151.000	0.170

CID94312	249.000	0.411	164.500	0.280
CID94715	318.602	0.219	180.000	0.508
CID95030	147.000	-	0.000	-
CID95054	210.632	-	0.000	-
CID95735	203.650	-	0.000	-
CID96356	307.281	-	0.000	-
CID96359	219.333	-	0.000	-
CID97226	295.421	-	0.000	-
CID97272	247.667	-	0.000	-
CID97283	324.833	-	0.000	-
CID97406	185.000	-	0.000	-
CID97530	221.500	-	0.000	-
CID97575	228.600	-	0.000	-
CID97703	189.000	-	0.000	-
CID97857	218.500	-	0.000	-
CID97865	276.500	-	0.000	-
CID98264	248.667	-	0.000	-
CID98487	210.741	0.544	277.000	0.133
CID98513	0.000	-	0.000	-
CID98792	370.517	-	0.000	-
CID99681	281.500	-	0.000	-
CID99735	370.655	-	0.000	-
CID99769	327.188	-	0.000	-
CID99959	0.000	-	0.000	-
CID100150	196.800	-	0.000	-
CID100153	352.467	0.191	181.000	0.019
CID100154	253.474	0.353	161.000	0.072
CID100175	360.500	-	0.000	-
CID100214	352.333	-	0.000	-
CID100255	0.000	-	0.000	-

CID100278	286.500	-	0.000	-
CID100323	218.875	-	0.000	-
CID100376	0.000	-	636.000	-
CID100427	324.750	0.216	165.000	0.022
CID100516	412.667	-	0.000	-
CID100521	0.000	-	0.000	-
CID100606	305.000	-	0.000	-
CID100668	301.333	-	0.000	-
CID100678	309.000	-	0.000	-
CID100708	242.500	-	0.000	-
CID100824	0.000	-	0.000	-
CID101190	0.000	-	0.000	-
CID101200	360.333	-	0.000	-
CID101524	195.783	-	0.000	-
CID102288	419.667	0.039	150.000	0.153
CID102682	0.000	-	0.000	-
CID102871	0.000	-	0.000	-
CID104625	505.000	-	0.000	-
CID104682	242.500	-	0.000	-
CID104727	275.967	0.248	419.800	0.100
CID104741	297.826	0.273	341.261	0.327
CID104746	299.600	0.175	181.000	0.071
CID104747	382.900	0.208	438.000	0.262
CID104758	273.742	-	0.000	-
CID104760	218.127	0.642	174.000	0.236
CID104762	357.167	-	0.000	-
CID104799	302.933	0.186	900.000	<0.001
CID104807	294.708	0.233	346.750	0.012
CID104810	269.351	0.372	268.000	0.180
CID104842	278.028	0.438	501.333	0.212

CID104849	298.571	0.364	150.000	0.288
CID104865	245.615	-	0.000	-
CID105035	303.802	0.311	189.000	0.067
CID105040	332.786	-	0.000	-
CID105111	274.283	0.314	226.000	0.073
CID105145	255.278	0.431	700.000	0.011
CID105148	237.272	-	0.000	-
CID105162	245.561	0.586	173.000	0.207
CID107744	351.947	0.242	900.000	0.031
CID107863	326.250	-	0.000	-
CID107865	311.625	0.131	164.000	0.011
CID107901	262.545	0.256	217.750	0.041
CID107935	267.276	0.197	420.538	0.087
CID108007	295.565	0.181	549.571	0.041
CID108150	233.792	0.331	533.000	0.080
CID108169	298.794	0.332	274.350	0.106
CID108188	311.045	-	0.000	-
CID108194	271.256	0.322	199.000	0.314
CID110634	240.625	0.305	153.000	0.193
CID110635	253.542	0.438	817.000	0.016
CID111123	244.219	0.230	207.429	0.031
CID111218	232.667	-	0.000	-
CID111332	240.167	0.172	188.000	0.054
CID112813	179.000	-	0.000	-
CID114586	148.000	-	0.000	-
CID114806	284.000	-	0.000	-
CID114861	334.200	-	0.000	-
CID114863	249.333	-	0.000	-
CID114873	304.855	0.235	206.333	0.345
CID114897	235.000	-	0.000	-

CID114933	244.250	-	0.000	-
CID114944	305.418	0.146	220.333	0.354
CID115102	199.100	-	0.000	-
CID115215	226.750	0.176	181.000	<0.001
CID115237	316.458	-	0.000	-
CID115384	231.000	-	0.000	-
CID115967	185.000	-	0.000	-
CID119031	333.152	0.212	676.000	0.174
CID119055	560.696	-	0.000	-
CID119182	291.121	0.153	496.000	0.036
CID119185	256.429	-	0.000	-
CID119196	327.750	0.152	150.000	0.095
CID119305	270.222	-	0.000	-
CID119373	265.963	0.331	997.000	<0.001
CID119378	280.364	-	0.000	-
CID119607	287.714	0.197	173.000	0.223
CID119828	311.870	-	0.000	-
CID120712	438.667	-	0.000	-
CID121591	285.643	0.238	177.500	0.111
CID121749	228.347	-	0.000	-
CID121955	262.000	-	0.000	-
CID122060	319.737	-	0.000	-
CID122335	224.688	-	0.000	-
CID122347	520.292	-	0.000	-
CID122797	272.429	-	0.000	-
CID122842	241.000	-	0.000	-
CID122877	221.333	0.176	175.000	0.018
CID123053	260.071	-	0.000	-
CID123151	259.200	0.584	166.000	0.438
CID123317	248.538	-	0.000	-

CID123606	238.063	0.173	800.000	0.025
CID123613	270.438	-	0.000	-
CID123617	343.714	-	0.000	-
CID123619	282.795	0.179	800.000	0.029
CID123628	215.214	-	0.000	-
CID123667	217.333	-	0.000	-
CID123714	176.000	-	0.000	-
CID123865	272.776	0.146	272.417	0.071
CID123964	301.467	-	0.000	-
CID124087	315.321	0.128	800.000	0.074
CID124109	204.000	-	0.000	-
CID124318	296.500	-	0.000	-
CID124331	470.667	0.006	724.000	<0.001
CID124946	238.600	-	0.000	-
CID125001	174.000	-	0.000	-
CID125196	205.000	-	0.000	-
CID125424	0.000	-	0.000	-
CID125704	243.667	-	0.000	-
CID125767	216.000	-	0.000	-
CID125843	165.000	-	0.000	-
CID125982	239.200	-	0.000	-
CID126337	179.500	-	0.000	-
CID126382	409.000	-	0.000	-
CID126545	247.500	-	0.000	-
CID126690	276.000	0.194	181.000	0.019
CID127650	232.800	-	0.000	-
CID127726	265.857	-	0.000	-
CID127963	230.000	-	0.000	-
CID128044	223.000	-	0.000	-
CID128068	0.000	-	0.000	-

CID128338	0.000	-	0.000	-
CID128803	219.000	-	0.000	-
CID128872	205.000	0.014	572.000	0.006
CID129195	177.333	-	0.000	-
CID129829	205.000	-	0.000	-
CID129999	176.000	-	0.000	-
CID130330	0.000	-	0.000	-
CID130333	422.000	-	0.000	-
CID130456	386.000	-	0.000	-
CID130731	316.429	-	0.000	-
CID130881	279.553	-	0.000	-
CID130966	237.714	0.146	377.600	0.031
CID131128	0.000	-	0.000	-
CID131182	276.143	-	0.000	-
CID131682	204.357	-	0.000	-
CID131995	198.000	-	0.000	-
CID132088	224.286	-	0.000	-
CID132114	284.500	-	0.000	-
CID132429	477.000	-	0.000	-
CID132444	179.500	-	0.000	-
CID132999	245.222	0.191	250.000	0.087
CID133006	0.000	-	0.000	-
CID133246	353.095	0.258	161.000	0.163
CID133411	232.571	-	0.000	-
CID133596	193.500	-	0.000	-
CID133911	0.000	-	0.000	-
CID134018	249.240	-	0.000	-
CID134020	0.000	-	0.000	-
CID134685	190.000	-	0.000	-
CID134966	226.750	-	0.000	-

CID135548	201.000	-	0.000	-
CID136539	214.535	0.365	168.333	0.063
CID137339	0.000	-	0.000	-
CID137767	238.435	-	0.000	-
CID140170	279.000	-	0.000	-
CID141643	380.098	0.219	232.250	0.176
CID145068	333.582	0.100	511.265	0.043
CID145729	425.953	0.379	923.000	0.005
CID146570	295.526	-	0.000	-
CID146621	231.500	-	0.000	-
CID146936	0.000	-	0.000	-
CID147515	279.833	-	0.000	-
CID147818	221.000	-	0.000	-
CID148121	293.320	0.466	166.000	0.066
CID148123	317.241	0.280	504.206	0.223
CID148147	233.000	-	0.000	-
CID148162	0.000	-	0.000	-
CID148177	286.189	0.219	463.917	0.104
CID148189	195.889	0.556	416.667	0.068
CID148191	230.818	0.211	212.000	0.047
CID148195	281.785	0.247	639.538	0.097
CID148201	289.391	0.195	968.000	<0.001
CID148202	243.000	-	0.000	-
CID148802	319.200	-	0.000	-
CID149096	380.610	0.185	493.500	0.067
CID149436	390.296	-	0.000	-
CID150311	273.245	0.305	487.500	0.145
CID151071	216.000	-	0.000	-
CID151072	173.250	-	0.000	-
CID151074	191.909	-	0.000	-

CID151114	280.350	-	0.000	-
CID151166	230.550	0.245	157.500	0.309
CID151173	229.440	-	0.000	-
CID151193	294.016	0.276	485.000	0.104
CID151199	268.901	0.398	204.857	0.203
CID151993	0.000	-	0.000	-
CID152704	0.000	-	0.000	-
CID152803	206.579	-	0.000	-
CID152917	315.444	-	0.000	-
CID153751	346.268	0.160	293.000	0.138
CID153941	241.642	-	0.000	-
CID153994	246.625	-	0.000	-
CID153997	294.263	0.124	864.000	<0.001
CID154058	197.045	0.682	187.000	0.067
CID154272	0.000	-	0.000	-
CID155232	194.000	-	0.000	-
CID155799	169.000	-	0.000	-
CID156391	295.317	0.262	337.667	0.180
CID156413	331.293	0.155	781.000	0.054
CID156418	268.891	0.331	169.000	0.205
CID156422	290.158	0.116	452.800	0.329
CID157348	239.857	-	0.000	-
CID157688	218.429	-	0.000	-
CID157879	0.000	-	0.000	-
CID157920	188.333	-	0.000	-
CID157921	266.579	-	0.000	-
CID158040	250.067	-	0.000	-
CID158371	0.000	-	0.000	-
CID158786	272.750	0.100	370.000	<0.001
CID159269	221.882	-	0.000	-

CID159324	302.207	0.185	331.300	0.077
CID159454	195.750	-	0.000	-
CID159594	242.500	0.228	167.000	0.065
CID159790	284.000	-	0.000	-
CID159832	274.809	0.122	311.625	0.116
CID159854	374.462	0.100	826.000	<0.001
CID160112	170.667	-	0.000	-
CID160202	212.333	-	0.000	-
CID160355	262.320	0.307	634.923	0.195
CID160368	0.000	-	0.000	-
CID160514	218.500	-	0.000	-
CID160913	420.188	0.460	336.000	0.041
CID161113	232.300	0.315	180.333	0.101
CID161383	316.579	-	0.000	-
CID162113	178.778	-	0.000	-
CID162637	205.043	-	0.000	-
CID163523	0.000	-	0.000	-
CID163659	230.582	0.311	562.000	0.121
CID164238	366.333	-	0.000	-
CID164259	193.000	-	0.000	-
CID164400	0.000	-	0.000	-
CID164462	181.500	-	0.000	-
CID164509	249.900	-	0.000	-
CID165213	134.714	-	0.000	-
CID165331	342.043	0.493	900.000	0.008
CID165904	248.429	0.207	205.000	0.028
CID166042	0.000	-	0.000	-
CID166558	249.583	-	0.000	-
CID166617	183.833	0.338	170.500	0.181
CID166760	439.563	-	0.000	-

CID167253	255.359	0.459	178.667	0.404
CID168030	279.720	-	0.000	-
CID168374	272.893	-	0.000	-
CID168545	235.000	-	0.000	-
CID168708	316.059	-	0.000	-
CID168924	279.214	-	0.000	-
CID169370	278.343	-	0.000	-
CID169446	0.000	-	0.000	-
CID170361	240.509	-	0.000	-
CID171548	326.224	-	0.000	-
CID172197	329.206	0.068	585.500	0.122
CID176166	256.868	0.231	706.556	0.075
CID176718	268.000	-	0.000	-
CID176873	273.917	0.184	170.500	0.105
CID177358	231.700	-	0.000	-
CID178038	0.000	-	0.000	-
CID179337	355.138	0.153	444.000	0.025
CID179344	314.313	-	0.000	-
CID181625	203.385	-	0.000	-
CID182762	327.571	-	0.000	-
CID184077	254.800	-	0.000	-
CID185184	0.000	-	0.000	-
CID185460	222.429	-	0.000	-
CID187790	281.840	0.345	210.200	0.278
CID188376	204.000	-	0.000	-
CID189821	297.143	-	0.000	-
CID189915	248.400	-	0.000	-
CID190217	228.053	-	0.000	-
CID190550	220.000	-	0.000	-
CID190763	337.000	-	0.000	-

CID191247	173.000	0.101	212.000	0.025
CID194173	234.080	0.122	161.000	0.052
CID194637	261.333	-	0.000	-
CID196373	303.100	-	0.000	-
CID196754	242.000	-	0.000	-
CID196786	205.400	-	0.000	-
CID196970	212.600	-	0.000	-
CID197256	199.833	-	0.000	-
CID198062	0.000	-	0.000	-
CID198790	277.750	-	0.000	-
CID198800	190.000	-	0.000	-
CID200599	902.000	-	0.000	-
CID208656	213.545	-	0.000	-
CID208898	269.259	0.242	150.000	0.147
CID208902	238.037	-	0.000	-
CID208908	307.412	0.303	504.750	0.119
CID213022	295.875	-	0.000	-
CID213040	299.300	0.110	282.000	0.022
CID213206	191.000	-	0.000	-
CID214902	0.000	-	0.000	-
CID216235	217.080	-	0.000	-
CID216237	228.160	0.404	429.000	0.061
CID216239	303.879	0.300	605.471	0.324
CID216240	180.000	-	0.000	-
CID216326	340.961	0.152	567.000	0.128
CID216453	462.923	0.127	172.333	0.060
CID216468	257.167	0.157	344.333	0.081
CID219018	197.529	-	0.000	-
CID219022	284.857	-	0.000	-
CID219023	242.600	0.284	170.000	0.019

CID219078	294.654	-	0.000	-
CID219100	238.000	0.134	170.000	0.065
CID219165	0.000	-	0.000	-
CID219166	0.000	-	0.000	-
CID220401	253.109	0.339	187.000	0.171
CID221493	376.721	0.166	339.923	0.622
CID222528	321.926	0.082	394.455	0.366
CID222786	296.228	0.130	707.000	0.046
CID232136	369.000	-	0.000	-
CID234103	262.500	-	0.000	-
CID235481	322.500	-	0.000	-
CID236668	166.000	-	0.000	-
CID240980	285.125	-	0.000	-
CID241902	248.089	0.401	827.000	<0.001
CID242437	364.667	-	0.000	-
CID243545	226.333	-	0.000	-
CID244818	179.000	-	0.000	-
CID244989	250.250	-	0.000	-
CID245869	299.700	-	0.000	-
CID246230	0.000	-	0.000	-
CID246231	505.000	-	0.000	-
CID249187	0.000	-	0.000	-
CID251636	220.571	-	0.000	-
CID252156	221.667	-	0.000	-
CID252682	352.778	0.114	613.000	0.014
CID253083	290.500	-	0.000	-
CID254916	166.000	-	0.000	-
CID254975	218.000	-	0.000	-
CID255402	186.000	-	0.000	-
CID256556	208.500	-	0.000	-

CID257608	340.630	-	0.000	-
CID259329	250.100	-	0.000	-
CID259331	281.959	0.360	166.000	0.130
CID260143	170.000	-	0.000	-
CID266285	219.545	-	0.000	-
CID266552	270.200	-	0.000	-
CID270820	209.000	-	0.000	-
CID271920	301.500	-	0.000	-
CID274726	249.000	-	0.000	-
CID275196	334.167	0.183	431.500	0.040
CID275978	161.000	-	0.000	-
CID276171	320.333	-	0.000	-
CID276175	258.000	-	0.000	-
CID277243	271.500	-	0.000	-
CID277387	0.000	-	0.000	-
CID277767	211.000	-	0.000	-
CID277822	307.318	-	0.000	-
CID278692	197.000	-	0.000	-
CID278767	312.500	-	0.000	-
CID279062	497.000	-	0.000	-
CID279748	376.500	-	0.000	-
CID280518	314.571	-	0.000	-
CID280526	278.000	-	0.000	-
CID280861	286.667	-	0.000	-
CID281604	176.000	-	0.000	-
CID281832	250.500	-	0.000	-
CID282479	321.286	-	0.000	-
CID283277	0.000	-	0.000	-
CID284536	302.333	-	0.000	-
CID285033	254.558	-	0.000	-

CID285701	511.000	-	0.000	-
CID286483	343.864	-	0.000	-
CID290525	247.000	-	0.000	-
CID292559	209.000	-	0.000	-
CID294641	237.000	-	0.000	-
CID296105	0.000	-	0.000	-
CID299867	392.333	-	0.000	-
CID300574	193.500	-	0.000	-
CID301389	279.565	0.296	180.000	0.034
CID304673	211.000	-	0.000	-
CID308111	305.667	-	0.000	-
CID308171	358.917	-	0.000	-
CID308963	0.000	-	0.000	-
CID309981	175.000	-	0.000	-
CID311931	278.000	-	0.000	-
CID312145	310.020	0.305	517.944	0.169
CID312287	527.500	-	0.000	-
CID312901	498.000	-	0.000	-
CID315320	191.000	-	0.000	-
CID315411	238.246	0.551	190.500	0.118
CID317560	326.429	-	0.000	-
CID317641	255.333	-	0.000	-
CID319771	196.000	-	0.000	-
CID320801	258.000	-	0.000	-
CID320803	267.500	-	0.000	-
CID323563	194.500	-	0.000	-
CID323701	198.000	-	0.000	-
CID324081	260.650	0.255	665.500	0.006
CID325910	242.500	-	0.000	-
CID325911	313.778	-	0.000	-

CID325912	225.800	-	0.000	-
CID326600	155.000	-	0.000	-
CID326834	455.500	-	0.000	-
CID327404	381.571	0.329	506.000	0.010
CID328137	190.000	-	0.000	-
CID328145	185.000	-	0.000	-
CID329826	288.000	-	0.000	-
CID330164	334.000	-	0.000	-
CID330851	405.000	-	0.000	-
CID331941	241.333	-	0.000	-
CID332237	458.833	-	0.000	-
CID333459	201.000	-	0.000	-
CID334410	498.000	-	0.000	-
CID334706	174.000	-	0.000	-
CID334838	226.000	-	0.000	-
CID335395	425.500	-	0.000	-
CID335981	300.125	-	0.000	-
CID336805	330.375	-	0.000	-
CID337012	557.000	-	0.000	-
CID337948	445.000	-	0.000	-
CID339411	377.500	-	0.000	-
CID341041	281.000	-	0.000	-
CID341263	176.000	-	0.000	-
CID341279	457.333	-	0.000	-
CID341280	385.000	-	0.000	-
CID341883	313.500	-	0.000	-
CID342397	377.741	-	0.000	-
CID342903	174.000	-	0.000	-
CID343070	211.000	-	0.000	-
CID343093	337.667	-	0.000	-

CID343097	285.429	-	0.000	-
CID344265	278.743	0.265	190.333	0.240
CID346727	226.500	-	0.000	-
CID347381	368.667	-	0.000	-
CID348175	325.375	-	0.000	-
CID348669	196.429	-	0.000	-
CID351143	215.000	-	0.000	-
CID352349	0.000	-	0.000	-
CID352938	538.000	-	0.000	-
CID352946	0.000	-	0.000	-
CID353016	380.500	-	0.000	-
CID353606	309.000	-	0.000	-
CID354175	304.000	-	0.000	-
CID354440	263.000	-	0.000	-
CID354572	174.000	-	0.000	-
CID354624	358.000	-	0.000	-
CID354677	233.167	-	0.000	-
CID354679	229.000	-	0.000	-
CID354767	359.600	-	0.000	-
CID355134	192.000	-	0.000	-
CID355392	217.000	-	0.000	-
CID356433	392.400	-	0.000	-
CID356434	411.250	-	0.000	-
CID357254	285.667	-	0.000	-
CID357989	497.000	-	0.000	-
CID358176	316.000	-	0.000	-
CID358641	355.552	0.243	209.500	0.139
CID358793	259.000	-	0.000	-
CID358880	242.500	0.056	159.000	0.009
CID359157	0.000	-	0.000	-

CID359414	224.000	-	0.000	-
CID359499	174.000	-	0.000	-
CID359756	0.000	-	0.000	-
CID359816	281.000	-	0.000	-
CID360414	228.000	-	0.000	-
CID360682	371.500	-	0.000	-
CID361311	249.000	-	0.000	-
CID361500	383.500	-	0.000	-
CID361501	300.000	-	0.000	-
CID361635	152.000	-	0.000	-
CID362422	363.833	-	0.000	-
CID362423	186.000	-	0.000	-
CID363634	290.500	-	0.000	-
CID363636	185.500	-	0.000	-
CID363644	0.000	-	0.000	-
CID363645	190.000	-	0.000	-
CID363647	233.000	-	0.000	-
CID363887	172.000	-	0.000	-
CID364089	0.000	-	0.000	-
CID364289	249.500	-	0.000	-
CID364586	215.000	-	0.000	-
CID364708	309.000	-	0.000	-
CID364716	0.000	-	0.000	-
CID366358	159.000	-	0.000	-
CID366819	162.500	-	0.000	-
CID366928	276.500	-	0.000	-
CID367405	171.000	-	0.000	-
CID368012	359.500	-	0.000	-
CID368107	0.000	-	0.000	-
CID368136	210.000	-	0.000	-

CID368141	163.000	-	0.000	-
CID368149	329.333	-	0.000	-
CID368250	243.438	-	0.000	-
CID368640	154.000	-	0.000	-
CID368696	253.667	-	0.000	-
CID369150	334.667	-	0.000	-
CID369910	255.250	-	0.000	-
CID369916	151.000	-	0.000	-
CID369918	152.000	-	0.000	-
CID369919	170.000	-	0.000	-
CID369922	214.500	-	0.000	-
CID369923	196.667	-	0.000	-
CID369924	0.000	-	0.000	-
CID369927	163.333	-	0.000	-
CID369949	0.000	-	0.000	-
CID369951	244.000	-	0.000	-
CID369961	323.333	-	0.000	-
CID369962	272.000	-	0.000	-
CID371509	180.000	0.328	250.000	0.012
CID371711	285.800	-	0.000	-
CID371712	192.000	-	0.000	-
CID372559	190.500	-	0.000	-
CID372869	258.667	-	0.000	-
CID372988	0.000	-	0.000	-
CID372992	175.000	-	0.000	-
CID372994	280.000	-	0.000	-
CID373001	234.500	-	0.000	-
CID373780	270.297	0.334	188.667	0.425
CID373798	155.000	-	0.000	-
CID373806	205.000	-	0.000	-

CID373969	0.000	-	0.000	-
CID374204	160.000	-	0.000	-
CID374273	201.000	-	0.000	-
CID374276	0.000	-	0.000	-
CID374628	210.500	-	0.000	-
CID374898	0.000	-	0.000	-
CID375123	184.000	-	0.000	-
CID375312	306.000	-	0.000	-
CID375860	285.000	-	0.000	-
CID375861	273.000	-	0.000	-
CID376098	0.000	-	0.000	-
CID376373	251.000	-	0.000	-
CID377395	285.500	-	0.000	-
CID377425	220.000	-	0.000	-
CID377426	391.333	-	0.000	-
CID377427	200.500	-	0.000	-
CID377468	477.000	-	0.000	-
CID378973	358.800	-	0.000	-
CID379107	337.400	-	0.000	-
CID379108	291.000	-	0.000	-
CID379284	314.333	-	0.000	-
CID379285	313.143	-	0.000	-
CID379286	298.800	-	0.000	-
CID379514	276.333	-	0.000	-
CID379556	333.600	-	0.000	-
CID379641	177.750	-	0.000	-
CID380865	170.000	-	0.000	-
CID380866	303.333	-	0.000	-
CID380867	206.000	-	0.000	-
CID380968	675.500	-	0.000	-

CID380970	457.600	-	0.000	-
CID381036	299.500	-	0.000	-
CID381128	327.667	-	0.000	-
CID381129	371.000	-	0.000	-
CID381130	331.400	-	0.000	-
CID381131	377.375	-	0.000	-
CID381133	371.286	-	0.000	-
CID381134	324.600	-	0.000	-
CID381135	241.750	-	0.000	-
CID381232	325.045	-	0.000	-
CID381234	360.667	-	0.000	-
CID381378	156.500	-	0.000	-
CID381387	153.000	-	0.000	-
CID381390	0.000	-	0.000	-
CID381392	205.000	-	0.000	-
CID381525	408.250	-	0.000	-
CID381595	355.500	-	0.000	-
CID382164	485.500	-	0.000	-
CID382367	156.000	-	0.000	-
CID382829	408.000	-	0.000	-
CID382830	371.000	-	0.000	-
CID383472	234.100	-	0.000	-
CID383473	231.500	-	0.000	-
CID383474	342.500	-	0.000	-
CID383475	224.333	-	0.000	-
CID383476	208.667	-	0.000	-
CID383481	0.000	-	0.000	-
CID384060	168.500	-	0.000	-
CID384061	189.000	-	0.000	-
CID384062	191.000	-	0.000	-

CID384063	209.000	-	0.000	-
CID384066	211.667	-	0.000	-
CID384067	199.667	-	0.000	-
CID384068	230.250	-	0.000	-
CID384217	321.000	-	0.000	-
CID384248	350.800	-	0.000	-
CID384249	356.000	-	0.000	-
CID384250	312.667	-	0.000	-
CID384253	366.154	-	0.000	-
CID384254	274.000	-	0.000	-
CID384258	432.500	-	0.000	-
CID384407	267.500	-	0.000	-
CID384478	169.000	-	0.000	-
CID384634	283.000	-	0.000	-
CID384795	212.500	-	0.000	-
CID385455	0.000	-	0.000	-
CID386431	169.000	-	0.000	-
CID386436	214.000	-	0.000	-
CID386496	242.000	-	0.000	-
CID386571	356.000	-	0.000	-
CID386586	262.000	-	0.000	-
CID386587	338.444	-	0.000	-
CID386588	220.000	-	0.000	-
CID386752	165.500	-	0.000	-
CID386904	421.000	-	0.000	-
CID387050	321.000	-	0.000	-
CID387052	364.250	-	0.000	-
CID387447	293.390	0.223	556.976	0.051
CID387684	229.500	-	0.000	-
CID387685	164.500	-	0.000	-

CID387686	202.667	-	0.000	-
CID387687	272.667	-	0.000	-
CID387951	359.538	-	0.000	-
CID388554	397.143	-	0.000	-
CID388556	347.333	-	0.000	-
CID388671	277.000	-	0.000	-
CID388985	0.000	-	0.000	-
CID389161	214.000	-	0.000	-
CID389396	0.000	-	0.000	-
CID389479	207.000	-	0.000	-
CID389510	204.000	-	0.000	-
CID389858	469.000	-	0.000	-
CID390191	246.000	-	0.000	-
CID390212	285.667	-	0.000	-
CID390521	0.000	-	0.000	-
CID390987	182.333	-	0.000	-
CID390988	209.500	-	0.000	-
CID391311	321.600	-	0.000	-
CID391584	446.250	-	0.000	-
CID391585	420.500	-	0.000	-
CID391586	280.750	-	0.000	-
CID391597	175.500	-	0.000	-
CID391856	354.000	-	0.000	-
CID392057	259.500	-	0.000	-
CID392061	172.000	-	0.000	-
CID392570	191.000	-	0.000	-
CID392571	190.500	-	0.000	-
CID392611	479.500	-	0.000	-
CID392613	549.000	-	0.000	-
CID392622	295.656	0.345	476.438	0.243

CID393098	290.500	-	0.000	-
CID393099	287.000	-	0.000	-
CID393100	281.500	-	0.000	-
CID393413	428.857	-	0.000	-
CID393470	373.500	-	0.000	-
CID393532	417.667	-	0.000	-
CID393599	355.769	-	0.000	-
CID393604	390.500	-	0.000	-
CID393605	410.667	-	0.000	-
CID393691	383.400	-	0.000	-
CID393692	417.250	-	0.000	-
CID393791	393.800	-	0.000	-
CID393792	555.000	-	0.000	-
CID393956	185.500	-	0.000	-
CID394048	185.000	-	0.000	-
CID394231	316.000	-	0.000	-
CID394236	311.333	-	0.000	-
CID394240	0.000	-	0.000	-
CID394347	950.000	0.012	979.000	<0.001
CID394348	166.000	-	0.000	-
CID394384	264.667	-	0.000	-
CID394386	0.000	-	0.000	-
CID394493	191.000	-	0.000	-
CID394646	169.000	-	0.000	-
CID394836	389.000	-	0.000	-
CID394871	194.000	-	0.000	-
CID395475	193.000	-	0.000	-
CID395559	451.500	-	0.000	-
CID395560	291.800	-	0.000	-
CID395561	391.333	-	0.000	-

CID395562	442.000	-	0.000	-
CID395621	302.667	-	0.000	-
CID395622	281.667	-	0.000	-
CID395803	291.333	-	0.000	-
CID395870	444.000	-	0.000	-
CID395871	311.000	-	0.000	-
CID395977	151.000	-	0.000	-
CID396011	365.667	-	0.000	-
CID396059	409.500	-	0.000	-
CID396064	179.000	-	0.000	-
CID396192	243.400	-	0.000	-
CID396198	474.500	-	0.000	-
CID396276	255.500	-	0.000	-
CID396466	0.000	-	0.000	-
CID396584	219.000	-	0.000	-
CID396901	187.000	-	0.000	-
CID397134	356.833	-	0.000	-
CID397887	315.444	-	0.000	-
CID398017	367.000	-	0.000	-
CID398330	245.300	-	0.000	-
CID398531	329.875	-	0.000	-
CID398532	298.167	-	0.000	-
CID399297	166.000	-	0.000	-
CID399617	327.167	-	0.000	-
CID399639	218.000	-	0.000	-
CID399990	253.000	-	0.000	-
CID400010	254.316	0.250	583.800	0.110
CID400499	378.500	-	0.000	-
CID400769	245.250	0.237	748.143	0.102
CID401169	175.000	-	0.000	-

CID402874	413.167	-	0.000	-
CID402877	380.625	-	0.000	-
CID403357	244.000	-	0.000	-
CID403577	314.500	-	0.000	-
CID403578	304.000	-	0.000	-
CID403923	346.324	0.189	604.000	0.012
CID404330	265.000	-	0.000	-
CID404841	208.857	-	0.000	-
CID404842	265.167	-	0.000	-
CID404843	345.286	-	0.000	-
CID404935	262.000	-	0.000	-
CID404936	0.000	-	0.000	-
CID405222	0.000	-	0.000	-
CID405950	0.000	-	0.000	-
CID406094	261.500	-	0.000	-
CID406499	306.750	-	0.000	-
CID406537	0.000	-	0.000	-
CID410253	229.529	0.223	201.000	0.012
CID410817	107.250	-	0.000	-
CID418098	364.500	-	0.000	-
CID418099	417.000	-	0.000	-
CID419263	414.000	-	0.000	-
CID420454	204.000	-	0.000	-
CID420455	0.000	-	0.000	-
CID420691	172.000	-	0.000	-
CID420759	220.500	-	0.000	-
CID420763	414.500	-	0.000	-
CID421299	352.652	-	0.000	-
CID421455	360.895	-	0.000	-
CID421884	253.000	-	0.000	-

CID422684	224.000	-	0.000	-
CID423209	205.000	0.049	719.000	<0.001
CID423893	269.667	-	0.000	-
CID425430	420.941	0.331	800.000	0.010
CID426244	409.000	-	0.000	-
CID429008	0.000	-	0.000	-
CID429342	245.000	-	0.000	-
CID429536	166.000	-	0.000	-
CID430475	239.500	-	0.000	-
CID430857	246.500	-	0.000	-
CID432762	121.400	-	0.000	-
CID434718	412.333	-	0.000	-
CID434973	532.333	-	0.000	-
CID435445	335.333	-	0.000	-
CID435681	212.750	-	0.000	-
CID435732	307.000	-	0.000	-
CID438410	167.000	-	0.000	-
CID438534	255.333	-	0.000	-
CID438545	165.000	-	0.000	-
CID438551	0.000	-	0.000	-
CID438894	197.000	-	0.000	-
CID438971	344.750	-	0.000	-
CID438992	232.000	-	0.000	-
CID438996	0.000	-	0.000	-
CID438998	241.250	-	0.000	-
CID439002	302.000	-	0.000	-
CID439010	400.500	-	0.000	-
CID439022	258.000	-	0.000	-
CID439023	202.000	-	0.000	-
CID439155	626.521	0.337	159.000	0.788

CID439175	645.280	-	0.000	-
CID439196	328.857	0.297	267.053	0.351
CID439234	392.909	-	0.000	-
CID439237	517.357	-	0.000	-
CID439260	408.736	0.036	623.000	0.524
CID439285	320.659	0.304	491.800	0.088
CID439353	394.900	0.123	284.222	0.406
CID439501	295.364	0.197	544.111	0.144
CID439520	272.452	0.141	171.000	0.401
CID439530	301.783	0.196	237.231	0.291
CID439655	330.659	0.349	900.000	<0.001
CID439693	291.111	0.234	363.500	0.085
CID439920	351.121	-	0.000	-
CID439960	42.350	-	0.000	-
CID440033	542.154	-	0.000	-
CID440474	414.833	-	0.000	-
CID441276	285.687	0.309	540.560	0.171
CID441542	300.203	0.300	225.533	0.338
CID442070	289.247	0.256	367.318	0.217
CID442514	287.967	0.261	282.065	0.108
CID442530	347.089	0.249	338.833	0.079
CID442972	318.452	0.249	372.571	0.157
CID442977	233.000	0.110	174.500	0.006
CID443090	298.719	0.192	205.000	0.131
CID443151	208.857	-	0.000	-
CID443154	293.567	0.242	199.000	0.135
CID443294	253.400	-	0.000	-
CID443314	293.157	0.122	245.814	0.128
CID443388	554.500	-	0.000	-
CID443484	506.000	-	0.000	-

CID443831	281.923	-	0.000	-
CID443872	328.968	-	0.000	-
CID444030	213.952	-	0.000	-
CID444208	171.500	-	0.000	-
CID444260	275.600	-	0.000	-
CID444503	180.714	0.270	159.000	0.172
CID444593	237.688	0.380	183.000	0.184
CID444732	322.560	0.327	399.607	0.332
CID444795	314.512	0.200	549.150	<0.001
CID444899	335.335	0.102	519.286	0.360
CID445008	197.714	0.283	184.000	0.033
CID445091	208.000	-	0.000	-
CID445117	663.500	-	0.000	-
CID445154	295.613	0.395	534.825	0.059
CID445226	293.783	0.312	165.000	0.124
CID445354	274.218	0.296	215.500	0.423
CID445533	236.357	0.334	163.000	0.100
CID445643	273.168	0.418	661.250	0.113
CID446087	209.447	-	0.000	-
CID446129	260.500	0.264	300.000	0.020
CID446157	275.106	0.249	155.500	0.367
CID446181	115.161	-	0.000	-
CID446313	304.889	0.149	336.000	0.025
CID446357	818.500	-	0.000	-
CID446378	305.279	0.288	643.455	0.064
CID446541	274.178	0.313	150.000	0.423
CID446556	254.500	-	0.000	-
CID446752	166.000	-	0.000	-
CID446838	291.657	0.238	276.750	0.140
CID447043	308.638	0.247	170.000	0.259

CID447077	303.375	0.155	506.667	0.365
CID447258	238.154	-	0.000	-
CID447316	288.952	0.248	528.333	0.070
CID447348	273.238	0.469	220.000	0.498
CID447356	265.214	0.448	223.714	0.293
CID447462	228.508	-	0.000	-
CID447612	229.700	0.137	160.000	0.069
CID447664	202.700	-	0.000	-
CID447700	386.769	0.136	486.500	0.064
CID447718	124.600	-	0.000	-
CID447833	189.786	-	0.000	-
CID447865	204.500	0.468	163.000	0.021
CID448013	216.870	0.378	161.000	0.022
CID448219	314.250	-	0.000	-
CID448537	327.376	0.078	518.846	0.379
CID448545	331.760	0.142	334.250	0.033
CID448657	186.938	0.337	196.000	0.115
CID448799	247.703	0.470	199.667	0.266
CID448839	262.297	0.455	182.000	0.230
CID448991	279.696	0.276	843.000	0.032
CID449005	331.071	0.321	264.000	0.387
CID449051	300.625	0.151	173.200	0.207
CID449129	210.667	-	0.000	-
CID449171	314.605	0.235	654.000	0.071
CID449193	284.741	0.232	810.000	0.055
CID449223	414.667	0.040	205.000	0.059
CID449459	293.626	0.301	537.630	0.084
CID449540	252.708	0.155	161.000	0.130
CID449546	233.053	0.306	340.000	0.022
CID450503	256.000	-	0.000	-

CID450516	262.626	0.412	205.400	0.470
CID451514	328.571	-	0.000	-
CID451668	311.253	0.305	467.773	0.097
CID451931	288.375	0.060	233.000	0.015
CID452548	331.041	0.215	463.333	0.052
CID455319	180.333	-	0.000	-
CID456130	34.000	-	0.000	-
CID456201	287.824	0.321	537.000	0.145
CID457785	271.842	-	0.000	-
CID457954	306.421	0.308	683.667	0.067
CID457964	272.455	0.292	239.857	0.327
CID459977	268.000	-	0.000	-
CID460612	356.776	0.136	265.000	0.138
CID461396	232.250	-	0.000	-
CID462186	174.667	-	0.000	-
CID466151	291.500	0.166	548.250	0.089
CID468595	265.902	-	0.000	-
CID472335	248.400	-	0.000	-
CID474409	243.636	-	0.000	-
CID478879	230.000	-	0.000	-
CID482128	264.250	-	0.000	-
CID484959	515.000	-	0.000	-
CID484960	420.500	-	0.000	-
CID484961	354.000	-	0.000	-
CID489614	324.750	-	0.000	-
CID493570	337.044	0.357	231.000	0.232
CID496212	274.000	-	0.000	-
CID496347	224.000	-	0.000	-
CID496913	250.093	-	0.000	-
CID513384	232.500	-	0.000	-

CID517045	288.767	0.119	186.200	0.440
CID518605	258.373	0.231	811.125	0.029
CID519332	184.167	-	0.000	-
CID520092	217.500	-	0.000	-
CID520535	317.240	0.265	388.517	0.198
CID521017	279.156	0.188	387.000	0.048
CID546075	224.476	0.599	159.000	0.246
CID566323	190.000	-	0.000	-
CID594789	189.750	-	0.000	-
CID626221	251.500	-	0.000	-
CID636283	148.667	-	0.000	-
CID636380	270.963	-	0.000	-
CID637463	26.000	-	0.000	-
CID637469	0.000	-	0.000	-
CID638072	334.842	0.194	207.000	0.232
CID639315	233.000	-	0.000	-
CID643078	181.718	-	0.000	-
CID643587	190.000	-	0.000	-
CID643668	232.400	-	0.000	-
CID644241	307.413	0.255	394.889	0.332
CID644350	391.273	-	0.000	-
CID656664	249.617	-	0.000	-
CID657090	197.750	-	0.000	-
CID657237	299.183	0.172	529.941	0.107
CID667490	346.162	0.248	478.143	0.155
CID668040	192.600	-	0.000	-
CID676166	262.647	-	0.000	-
CID683248	321.400	-	0.000	-
CID688222	220.000	-	0.000	-
CID688442	0.000	-	0.000	-

CID720071	278.155	0.263	194.667	0.159
CID725168	219.000	-	0.000	-
CID753704	282.188	-	0.000	-
CID820318	205.667	-	0.000	-
CID901806	0.000	-	0.000	-
CID969472	235.025	-	0.000	-
CID1008484	214.500	-	0.000	-
CID1048845	293.763	0.264	203.000	0.156
CID1106052	244.400	-	0.000	-
CID1202545	0.000	-	0.000	-
CID1377751	222.500	-	0.000	-
CID1474853	211.059	0.310	714.500	0.082
CID1474860	0.000	-	254.500	-
CID1548886	286.584	0.297	440.519	0.222
CID1548999	276.690	0.182	187.000	0.057
CID1893730	301.313	0.261	538.000	0.009
CID2238111	226.091	-	0.000	-
CID2289475	0.000	-	0.000	-
CID2594324	206.000	-	0.000	-
CID2723601	306.632	0.316	321.500	0.129
CID2724126	220.648	0.322	168.500	0.095
CID2724189	221.063	0.334	207.000	0.016
CID2724385	297.159	0.206	498.400	0.104
CID2724387	253.120	0.419	800.000	<0.001
CID2724884	99.000	-	0.000	-
CID2725144	170.000	-	0.000	-
CID2725145	198.667	-	0.000	-
CID2725194	0.000	-	0.000	-
CID2725429	193.400	-	0.000	-
CID2725653	208.000	-	0.000	-

CID2725672	296.714	-	0.000	-
CID2726466	253.000	-	0.000	-
CID2726501	178.000	-	0.000	-
CID2726502	258.750	-	0.000	-
CID2726536	218.400	-	0.000	-
CID2726541	234.750	-	0.000	-
CID2733525	308.399	0.321	579.093	0.066
CID2760897	197.143	-	0.000	-
CID2761467	191.667	-	0.000	-
CID2782689	257.667	0.429	638.000	0.029
CID2795457	210.091	0.254	166.000	0.053
CID2797650	0.000	-	0.000	-
CID2807595	217.833	0.325	330.000	0.020
CID2808828	0.000	-	0.000	-
CID2812173	266.578	0.413	253.333	0.192
CID2987927	247.867	0.231	181.000	0.201
CID3001028	330.225	0.238	368.417	0.181
CID3001029	267.200	-	0.000	-
CID3001055	263.044	0.270	814.000	0.021
CID3003994	591.500	-	0.000	-
CID3005572	290.563	0.288	594.000	0.094
CID3005582	0.000	-	0.000	-
CID3006484	297.667	-	0.000	-
CID3006531	287.603	0.193	471.810	0.194
CID3010858	205.000	-	0.000	-
CID3011155	203.963	-	0.000	-
CID3013632	271.091	-	0.000	-
CID3015145	0.000	-	0.000	-
CID3018505	315.533	-	0.000	-
CID3025986	244.636	0.239	493.000	0.142

CID3031661	265.850	0.209	208.000	0.007
CID3032417	377.833	-	0.000	-
CID3032581	297.288	0.181	243.737	0.286
CID3033963	381.200	-	0.000	-
CID3034010	235.721	0.309	747.000	0.079
CID3034013	210.667	-	0.000	-
CID3034391	286.500	-	0.000	-
CID3034646	336.571	-	0.000	-
CID3034746	217.938	-	0.000	-
CID3035239	239.000	-	0.000	-
CID3035240	233.000	-	0.000	-
CID3035714	212.167	-	0.000	-
CID3035755	233.000	-	0.000	-
CID3035882	201.750	-	0.000	-
CID3036505	299.400	-	0.000	-
CID3037617	282.857	0.170	407.000	0.026
CID3037883	423.000	-	0.000	-
CID3038497	240.000	-	0.000	-
CID3038522	291.742	0.221	317.000	0.123
CID3040488	205.667	-	0.000	-
CID3040815	214.667	-	0.000	-
CID3051113	282.000	-	0.000	-
CID3052762	198.667	-	0.000	-
CID3052775	279.061	0.286	900.000	0.048
CID3053112	324.000	-	0.000	-
CID3062316	311.517	0.296	521.029	0.076
CID3070537	215.364	0.417	243.500	0.090
CID3080544	283.333	-	0.000	-
CID3080607	0.000	-	0.000	-
CID3080619	321.100	-	0.000	-

CID3080716	0.000	-	0.000	-
CID3080757	254.667	-	0.000	-
CID3081361	325.111	0.300	471.059	0.324
CID3082777	214.909	0.240	263.000	0.036
CID3083352	213.000	-	0.000	-
CID3083408	230.250	-	0.000	-
CID3084046	279.033	0.130	234.000	0.223
CID3084116	216.000	-	0.000	-
CID3086257	360.571	-	0.000	-
CID3246719	307.033	0.619	163.000	0.104
CID3428705	168.000	-	0.000	-
CID3449946	0.000	-	0.000	-
CID3478765	205.000	-	0.000	-
CID3480542	205.667	-	0.000	-
CID3658531	239.950	-	0.000	-
CID3664418	356.667	-	0.000	-
CID3733518	301.800	0.267	773.000	<0.001
CID3795021	404.500	-	0.000	-
CID3807643	153.000	-	0.000	-
CID4056967	256.000	-	0.000	-
CID4297981	191.000	-	0.000	-
CID4357887	0.000	-	0.000	-
CID4369270	223.806	0.520	150.000	0.193
CID4369446	311.917	-	0.000	-
CID4404138	220.000	-	0.000	-
CID4602007	427.333	-	0.000	-
CID4678317	213.143	-	0.000	-
CID4806509	432.000	-	0.000	-
CID4990817	442.333	-	0.000	-
CID4998669	190.421	0.322	719.000	0.016

CID5001396	275.994	0.319	500.750	0.172
CID5023375	262.667	-	0.000	-
CID5222465	269.660	0.142	583.636	0.098
CID5231521	279.000	-	0.000	-
CID5273370	223.500	-	0.000	-
CID5280343	316.459	0.072	370.477	<0.001
CID5280352	303.794	0.262	301.000	0.385
CID5280360	344.820	0.093	749.150	0.292
CID5280453	296.095	0.371	435.429	0.002
CID5280483	297.688	0.392	528.000	0.086
CID5280492	346.632	0.242	609.231	0.443
CID5280493	409.222	0.237	711.571	0.556
CID5280961	303.970	0.328	508.264	0.010
CID5280965	277.540	0.250	181.625	0.394
CID5281004	324.427	0.243	410.667	0.232
CID5281040	357.857	0.272	374.000	0.202
CID5281051	253.649	0.321	248.000	0.096
CID5281078	297.960	0.359	390.667	0.163
CID5281321	244.235	0.280	612.333	0.043
CID5281614	235.907	0.202	462.200	0.163
CID5281767	281.456	0.333	631.550	0.132
CID5281819	182.750	-	0.000	-
CID5281828	244.200	0.423	171.333	0.096
CID5281877	271.576	0.152	293.000	0.247
CID5281888	307.143	0.160	807.000	0.048
CID5281915	272.818	0.539	241.615	0.138
CID5281955	264.110	0.250	529.333	0.010
CID5281967	242.667	-	0.000	-
CID5282375	317.250	0.230	744.500	0.071
CID5282379	269.903	0.308	364.714	0.016

CID5282381	273.056	0.296	707.000	0.050
CID5282411	366.387	0.272	683.667	0.382
CID5282412	270.324	0.266	239.000	0.072
CID5282442	261.939	0.496	182.000	0.305
CID5282451	255.974	0.171	151.000	0.284
CID5282497	416.833	-	0.000	-
CID5284344	235.738	0.317	800.000	<0.001
CID5284371	335.224	0.178	245.000	0.238
CID5284380	254.348	0.308	159.000	0.046
CID5284448	200.848	-	0.000	-
CID5284513	308.053	0.244	307.143	0.068
CID5284558	220.208	0.143	800.000	0.008
CID5284566	271.982	0.466	159.000	0.164
CID5284616	293.316	0.307	552.956	0.208
CID5287457	204.958	-	0.000	-
CID5287587	81.143	0.498	900.000	<0.001
CID5287955	900.000	-	0.000	-
CID5287969	243.558	0.300	598.095	0.235
CID5288014	316.727	-	0.000	-
CID5288209	278.031	0.230	416.105	0.305
CID5288382	283.500	0.342	556.313	0.150
CID5288628	196.780	0.209	900.000	<0.001
CID5288693	0.000	-	519.000	-
CID5288718	206.094	-	0.000	-
CID5288783	343.176	0.241	800.000	0.040
CID5288818	260.333	-	0.000	-
CID5288826	348.449	0.091	704.154	0.161
CID5289072	216.698	-	0.000	-
CID5289247	239.367	0.312	331.400	0.086
CID5289419	213.882	0.231	820.667	0.044

CID5311051	264.536	0.219	150.000	<0.001
CID5311181	263.790	0.368	678.000	0.184
CID5311263	265.007	0.270	393.133	0.137
CID5311497	281.524	0.231	381.000	0.006
CID5311498	299.507	0.310	171.000	0.169
CID5311510	324.870	-	0.000	-
CID5312804	208.800	-	0.000	-
CID5318183	0.000	-	0.000	-
CID5319777	0.000	-	0.000	-
CID5321426	281.000	-	0.000	-
CID5321702	203.500	-	0.000	-
CID5321748	0.000	-	0.000	-
CID5323510	406.304	0.181	475.000	0.009
CID5326861	228.119	0.524	285.667	0.019
CID5327044	259.000	0.203	161.000	0.205
CID5327065	0.000	-	177.667	-
CID5328779	283.261	0.214	550.167	0.209
CID5328940	316.786	0.211	643.000	0.335
CID5329006	210.333	0.129	444.500	0.012
CID5329032	284.778	0.076	313.250	0.034
CID5329098	264.357	0.379	450.900	0.239
CID5329102	293.690	0.411	538.900	0.526
CID5330175	330.000	0.057	317.500	0.024
CID5330197	38.000	-	0.000	-
CID5338590	169.000	-	0.000	-
CID5340178	177.000	-	0.000	-
CID5351132	442.250	-	0.000	-
CID5351153	283.211	-	0.000	-
CID5351254	187.000	-	0.000	-
CID5351307	312.167	-	0.000	-

CID5351332	400.750	-	0.000	-
CID5351344	246.560	0.711	162.000	0.226
CID5351347	203.500	-	0.000	-
CID5351360	192.867	0.627	434.000	0.016
CID5351386	250.867	-	0.000	-
CID5352019	301.727	0.275	250.800	0.016
CID5353425	224.000	-	0.000	-
CID5353562	371.085	0.237	386.143	0.103
CID5353586	310.906	0.055	352.778	0.184
CID5353888	174.500	-	0.000	-
CID5353980	298.393	0.232	228.500	0.145
CID5354357	160.718	0.570	239.000	0.025
CID5354883	221.000	-	0.000	-
CID5358467	284.500	-	0.000	-
CID5359264	278.792	0.347	237.643	0.238
CID5359282	136.000	0.436	188.000	<0.001
CID5359476	274.347	0.276	472.250	0.194
CID5359596	286.528	0.289	454.047	0.366
CID5360515	272.992	0.357	800.000	0.035
CID5360588	234.905	-	0.000	-
CID5360596	248.400	-	0.000	-
CID5360621	289.889	0.276	199.500	0.146
CID5362070	364.167	-	0.000	-
CID5362372	312.385	-	0.000	-
CID5362420	292.846	0.221	327.000	0.026
CID5362440	287.255	0.326	784.000	0.080
CID5362564	220.283	0.440	206.111	0.110
CID5367237	209.000	-	0.000	-
CID5367272	219.600	-	0.000	-
CID5370875	353.333	-	0.000	-

CID5372567	0.000	-	0.000	-
CID5372798	355.692	-	0.000	-
CID5374787	456.000	-	0.000	-
CID5378367	200.000	-	0.000	-
CID5380438	193.000	-	0.000	-
CID5381226	303.328	0.233	389.125	0.188
CID5381230	181.000	-	0.000	-
CID5384001	318.929	-	0.000	-
CID5384098	311.000	-	0.000	-
CID5385089	194.000	-	0.000	-
CID5385554	155.000	-	0.000	-
CID5386092	275.138	0.230	167.000	0.158
CID5388755	218.500	-	0.000	-
CID5388767	190.500	-	0.000	-
CID5388779	0.000	-	0.000	-
CID5388861	222.500	-	0.000	-
CID5388862	0.000	-	0.000	-
CID5458428	284.571	0.311	559.429	0.067
CID5459063	233.000	-	0.000	-
CID5459073	227.833	-	0.000	-
CID5459262	352.500	-	0.000	-
CID5459858	341.200	-	0.000	-
CID5459918	509.444	-	0.000	-
CID5460029	106.500	-	0.000	-
CID5460037	284.910	0.029	199.867	0.522
CID5460048	349.228	0.090	230.455	0.694
CID5460448	759.500	-	0.000	-
CID5460555	235.169	0.327	176.000	0.093
CID5460556	234.057	0.426	151.000	0.357
CID5460631	784.674	-	0.000	-

CID5461123	275.822	0.165	165.000	0.409
CID5462099	247.586	0.511	191.000	0.083
CID5462328	293.743	0.242	365.000	0.052
CID5462337	281.882	-	0.000	-
CID5463137	357.600	-	0.000	-
CID5464084	247.000	-	0.000	-
CID5464850	327.000	-	0.000	-
CID5464851	383.667	-	0.000	-
CID5465490	230.000	-	0.000	-
CID5465913	0.000	-	0.000	-
CID5465919	251.000	-	0.000	-
CID5465920	262.000	-	0.000	-
CID5465988	259.000	-	0.000	-
CID5465991	359.000	-	0.000	-
CID5466067	248.500	-	0.000	-
CID5466480	261.000	-	0.000	-
CID5466667	592.000	-	0.000	-
CID5467019	0.000	-	0.000	-
CID5467055	272.800	-	0.000	-
CID5467340	343.000	-	0.000	-
CID5467416	164.000	-	0.000	-
CID5467420	0.000	-	0.000	-
CID5467768	306.000	-	0.000	-
CID5468270	395.500	-	0.000	-
CID5468287	251.000	-	0.000	-
CID5468408	378.333	-	0.000	-
CID5468409	513.500	-	0.000	-
CID5468506	204.000	-	0.000	-
CID5468574	184.667	-	0.000	-
CID5468688	291.667	-	0.000	-

CID5468689	172.500	-	0.000	-
CID5468698	250.667	-	0.000	-
CID5468914	163.000	-	0.000	-
CID5468915	306.000	-	0.000	-
CID5468960	236.800	-	0.000	-
CID5468967	364.833	-	0.000	-
CID5469103	228.500	-	0.000	-
CID5469120	291.500	-	0.000	-
CID5469122	524.500	-	0.000	-
CID5469144	269.750	-	0.000	-
CID5469147	0.000	-	0.000	-
CID5469318	239.778	0.133	441.111	0.044
CID5469762	315.500	-	0.000	-
CID5469772	475.000	-	0.000	-
CID5469809	247.000	-	0.000	-
CID5470051	170.000	-	0.000	-
CID5470230	493.500	-	0.000	-
CID5470366	231.500	-	0.000	-
CID5470409	253.000	-	0.000	-
CID5470410	163.000	-	0.000	-
CID5470965	256.750	-	0.000	-
CID5471488	223.000	-	0.000	-
CID5471969	0.000	-	0.000	-
CID5472495	290.684	0.170	627.636	0.127
CID5472884	340.000	-	0.000	-
CID5473339	458.667	-	0.000	-
CID5474206	253.826	0.351	514.000	<0.001
CID5476374	245.237	0.343	249.667	0.175
CID5477614	271.259	0.163	199.000	0.010
CID5478977	0.000	-	0.000	-

CID5479494	186.800	-	0.000	-
CID5479530	394.889	0.217	199.000	0.198
CID5480431	245.330	0.276	812.000	0.015
CID5481173	349.744	0.259	164.000	0.435
CID5481306	229.333	-	0.000	-
CID5481307	179.000	-	0.000	-
CID5481350	317.400	0.231	700.000	<0.001
CID5483659	167.000	-	0.000	-
CID5484390	196.000	-	0.000	-
CID5484402	227.667	-	0.000	-
CID5484731	286.000	0.252	161.000	0.149
CID5485223	207.444	-	0.000	-
CID5486799	248.444	-	0.000	-
CID5487262	274.500	-	0.000	-
CID5487301	217.931	0.567	800.000	0.059
CID5487322	302.600	-	0.000	-
CID5487525	278.938	0.096	344.750	0.022
CID5488547	208.533	-	0.000	-
CID5488855	307.500	-	0.000	-
CID5488895	164.625	-	0.000	-
CID5489033	145.250	-	0.000	-
CID5489042	195.929	-	0.000	-
CID5489047	174.000	-	0.000	-
CID5489345	210.000	-	0.000	-
CID5489429	201.333	-	0.000	-
CID5489467	102.000	-	0.000	-
CID5491524	166.000	-	0.000	-
CID5491932	220.000	-	0.000	-
CID5493381	216.290	-	0.000	-
CID5493444	297.809	-	0.000	-

CID5494319	258.000	-	0.000	-
CID5494336	188.000	-	0.000	-
CID5494341	230.000	-	0.000	-
CID5494343	241.333	-	0.000	-
CID5494351	303.000	-	0.000	-
CID5494354	498.400	-	0.000	-
CID5494388	231.178	-	0.000	-
CID5494407	215.875	0.385	214.000	0.064
CID5494424	216.850	0.229	180.000	0.115
CID5494449	290.323	0.296	507.818	0.316
CID5497150	202.071	-	0.000	-
CID5702003	223.000	-	0.000	-
CID5702211	195.167	-	0.000	-
CID5702402	0.000	-	0.000	-
CID5702553	278.748	0.292	211.619	0.174
CID5745032	0.000	-	0.000	-
CID5748301	44.000	-	0.000	-
CID5748311	188.400	-	0.000	-
CID5748525	226.833	-	0.000	-
CID5785686	267.500	-	0.000	-
CID5834397	253.000	-	0.000	-
CID5843399	249.750	-	0.000	-
CID5864210	188.000	-	0.000	-
CID5871897	257.000	-	0.000	-
CID5892010	266.500	-	0.000	-
CID5907304	217.000	-	0.000	-
CID5919495	219.000	-	0.000	-
CID5923821	267.000	-	0.000	-
CID5923825	271.000	-	0.000	-
CID5927311	176.000	-	0.000	-

CID5930524	164.000	-	0.000	-
CID5930549	172.500	-	0.000	-
CID6091659	275.095	0.072	332.833	0.006
CID6102657	227.000	-	0.000	-
CID6167828	301.064	0.283	211.500	0.169
CID6279389	210.000	-	0.000	-
CID6309639	0.000	-	0.000	-
CID6321408	267.716	-	0.000	-
CID6323367	187.167	-	0.000	-
CID6323490	302.832	0.270	222.167	0.212
CID6324671	319.435	0.224	259.667	0.235
CID6328190	201.773	-	0.000	-
CID6331826	456.625	-	0.000	-
CID6335178	230.000	-	0.000	-
CID6336144	185.000	-	0.000	-
CID6337614	220.273	-	0.000	-
CID6364505	257.333	-	0.000	-
CID6398629	228.333	-	0.000	-
CID6419957	303.813	0.117	249.880	0.127
CID6420135	199.429	0.253	157.000	0.045
CID6432013	228.671	0.395	163.000	0.119
CID6433076	187.375	-	0.000	-
CID6433082	426.000	-	0.000	-
CID6433191	157.000	-	0.000	-
CID6433272	249.861	-	0.000	-
CID6433557	296.887	0.344	392.500	0.265
CID6434870	221.673	-	0.000	-
CID6435110	285.315	0.431	368.750	0.085
CID6435755	381.400	-	0.000	-
CID6436750	0.000	-	0.000	-

CID6437358	205.875	-	0.000	-
CID6438581	278.333	-	0.000	-
CID6438891	190.000	0.396	163.000	0.028
CID6438895	227.750	-	0.000	-
CID6439072	235.714	0.251	399.000	<0.001
CID6439494	100.000	-	0.000	-
CID6440032	249.333	-	0.000	-
CID6440175	266.241	0.256	491.600	0.132
CID6440853	172.500	-	0.000	-
CID6441311	0.000	-	0.000	-
CID6442177	295.770	0.325	290.063	0.177
CID6444692	343.333	0.071	817.500	0.006
CID6445533	325.163	0.223	500.667	0.194
CID6445540	248.571	0.324	205.667	0.016
CID6445562	322.081	0.153	605.833	0.112
CID6447311	186.200	-	0.000	-
CID6450551	292.765	0.266	352.286	0.197
CID6450813	226.474	0.347	564.000	0.014
CID6450816	224.750	-	0.000	-
CID6456014	214.083	-	0.000	-
CID6456015	254.679	0.245	323.500	0.110
CID6481127	247.000	-	0.000	-
CID6505803	292.837	0.275	576.889	0.098
CID6509979	245.463	-	0.000	-
CID6516858	0.000	-	0.000	-
CID6519698	285.500	-	0.000	-
CID6540268	230.660	0.274	224.333	0.216
CID6540295	234.803	0.384	190.250	0.046
CID6540456	292.000	-	0.000	-
CID6560141	237.554	-	0.000	-

CID6603746	286.867	-	0.000	-
CID6603857	239.833	0.204	616.667	0.018
CID6604200	329.448	0.255	454.000	0.034
CID6711219	243.500	-	0.000	-
CID6711222	240.923	-	0.000	-
CID6711240	274.333	-	0.000	-
CID6711246	184.000	-	0.000	-
CID6711254	0.000	-	0.000	-
CID6711255	0.000	-	0.000	-
CID6711269	267.000	-	0.000	-
CID6711270	260.500	-	0.000	-
CID6711584	187.600	-	0.000	-
CID6711820	251.500	-	0.000	-
CID6711944	177.000	-	0.000	-
CID6711997	242.000	-	0.000	-
CID6712095	209.500	-	0.000	-
CID6712096	159.000	-	0.000	-
CID6712194	166.000	-	0.000	-
CID6712215	210.500	-	0.000	-
CID6712242	267.000	-	0.000	-
CID6712270	249.000	-	0.000	-
CID6712271	235.667	-	0.000	-
CID6712409	285.000	-	0.000	-
CID6712471	259.500	-	0.000	-
CID6712547	445.000	-	0.000	-
CID6712549	524.000	-	0.000	-
CID6712626	211.333	-	0.000	-
CID6712908	248.000	-	0.000	-
CID6713928	213.184	0.234	233.667	0.040
CID6850715	281.100	0.284	220.739	0.221

CID6850719	144.768	0.611	175.000	0.029
CID6850726	234.071	0.172	271.167	0.014
CID6850753	247.951	0.164	227.333	0.291
CID6857944	217.857	-	0.000	-
CID6913191	146.000	-	0.000	-
CID6914628	188.667	0.200	521.000	0.042
CID6917781	209.200	0.357	800.000	0.019
CID6917838	196.000	-	0.000	-
CID6918054	238.500	-	0.000	-
CID6918091	216.667	-	0.000	-
CID6918107	191.500	-	0.000	-
CID6918164	312.000	-	0.000	-
CID6918182	269.113	-	0.000	-
CID6918220	248.080	0.294	181.000	0.029
CID6918250	250.667	0.227	170.000	0.074
CID6918259	193.500	-	0.000	-
CID6918289	272.739	0.333	325.154	0.097
CID6918296	259.465	0.210	150.000	0.110
CID6918310	212.375	-	0.000	-
CID6918317	265.000	-	0.000	-
CID6918321	243.000	-	0.000	-
CID6918335	185.500	-	0.000	-
CID6918347	209.182	-	0.000	-
CID6918365	246.510	0.442	162.000	0.162
CID6918403	305.217	0.127	677.000	0.034
CID6918404	235.700	-	0.000	-
CID6918407	187.222	-	0.000	-
CID6918412	260.583	0.230	425.000	0.017
CID6918453	271.946	0.277	499.500	0.032
CID6918454	284.618	0.230	313.643	0.120

CID6918456	277.938	0.306	380.500	0.080
CID6918457	185.714	-	0.000	-
CID6918461	242.833	-	0.000	-
CID6918473	193.375	-	0.000	-
CID6918491	261.000	-	0.000	-
CID6918493	271.563	-	0.000	-
CID6918500	233.000	-	0.000	-
CID6918506	94.000	-	0.000	-
CID6918508	229.333	0.174	725.500	0.005
CID6918537	262.895	0.272	196.000	0.081
CID6918540	415.778	-	0.000	-
CID6918554	271.261	-	0.000	-
CID6918558	228.091	0.432	800.000	0.017
CID6918589	314.000	-	0.000	-
CID6918638	349.538	0.158	411.800	0.131
CID6918712	185.500	-	0.000	-
CID6918835	257.333	-	0.000	-
CID6918837	330.721	0.198	606.000	0.162
CID6931237	307.500	-	0.000	-
CID6950200	26.000	-	0.000	-
CID6950523	124.600	-	0.000	-
CID6995012	172.120	-	0.000	-
CID7009631	250.000	-	0.000	-
CID7010485	0.000	-	0.000	-
CID7018299	233.000	-	0.000	-
CID9547169	216.412	0.135	202.500	0.039
CID9547891	241.333	-	0.000	-
CID9547917	213.647	0.365	159.000	0.064
CID9547920	192.583	-	0.000	-
CID9547971	47.765	-	0.000	-

CID9548778	221.286	-	0.000	-
CID9549284	283.872	0.256	300.600	0.103
CID9549297	261.824	0.329	390.357	0.304
CID9549299	283.364	0.320	338.143	0.085
CID9554281	212.571	-	0.000	-
CID9554740	235.250	-	0.000	-
CID9554745	195.000	-	0.000	-
CID9554753	222.500	-	0.000	-
CID9556392	177.000	-	0.000	-
CID9562060	280.510	0.234	330.143	0.164
CID9566088	277.500	-	0.000	-
CID9566247	205.000	-	0.000	-
CID9566249	247.000	-	0.000	-
CID9566250	165.000	-	0.000	-
CID9568512	258.474	0.383	205.333	0.095
CID9568613	210.333	-	0.000	-
CID9568614	185.375	-	0.000	-
CID9569925	0.000	-	0.000	-
CID9570480	197.000	-	0.000	-
CID9571616	273.000	-	0.000	-
CID9571617	0.000	-	0.000	-
CID9571636	169.000	-	0.000	-
CID9571679	0.000	-	0.000	-
CID9571836	210.826	0.493	235.000	0.038
CID9571908	338.400	-	0.000	-
CID9572047	213.500	-	0.000	-
CID9572090	244.000	-	0.000	-
CID9572091	174.000	-	0.000	-
CID9572386	239.500	-	0.000	-
CID9574101	264.696	-	0.000	-

CID9574395	437.500	-	0.000	-
CID9576037	223.733	-	0.000	-
CID9576411	259.273	-	0.000	-
CID9576912	212.750	-	0.000	-
CID9577124	242.273	-	0.000	-
CID9577595	0.000	-	0.000	-
CID9578572	237.583	-	0.000	-
CID9589473	198.528	-	0.000	-
CID9630043	319.000	-	0.000	-
CID9690109	203.333	-	0.000	-
CID9690117	386.000	-	0.000	-
CID9795739	202.600	-	0.000	-
CID9796068	261.750	0.185	180.000	0.051
CID9800306	244.700	0.205	800.000	<0.001
CID9801640	230.667	-	0.000	-
CID9802561	207.875	-	0.000	-
CID9804302	351.800	0.157	425.000	0.067
CID9804991	309.354	0.315	356.400	0.321
CID9806229	335.500	-	0.000	-
CID9807128	270.667	-	0.000	-
CID9808225	217.560	-	0.000	-
CID9809714	247.000	0.311	778.000	0.182
CID9810373	0.000	-	0.000	-
CID9810701	281.000	-	0.000	-
CID9811076	306.333	-	0.000	-
CID9811638	356.143	-	0.000	-
CID9820073	196.750	-	0.000	-
CID9825149	273.368	0.195	700.000	0.016
CID9826528	307.400	0.228	443.583	0.050
CID9828138	213.250	-	0.000	-

CID9829639	197.389	-	0.000	-
CID9829828	240.333	-	0.000	-
CID9832423	268.556	-	0.000	-
CID9832447	254.500	0.155	575.000	0.031
CID9832687	195.250	-	0.000	-
CID9832804	210.077	-	0.000	-
CID9838021	199.688	-	0.000	-
CID9838389	245.857	-	0.000	-
CID9843206	269.286	0.256	436.000	0.049
CID9846537	203.600	-	0.000	-
CID9849735	372.050	0.136	386.100	0.047
CID9854073	326.000	0.210	823.000	<0.001
CID9855343	205.667	-	0.000	-
CID9859947	220.040	-	0.000	-
CID9863342	354.300	0.141	432.625	0.043
CID9865515	315.500	0.142	530.500	0.082
CID9865808	328.103	-	0.000	-
CID9867642	248.000	-	0.000	-
CID9867822	279.440	-	0.000	-
CID9868037	222.556	0.179	700.000	0.015
CID9868332	367.857	-	0.000	-
CID9868524	233.353	0.185	372.500	0.086
CID9869929	207.300	-	0.000	-
CID9871747	214.444	-	0.000	-
CID9874151	147.714	-	0.000	-
CID9874248	207.154	-	0.000	-
CID9874912	214.143	0.236	753.667	0.013
CID9875401	238.939	-	0.000	-
CID9876867	189.636	-	0.000	-
CID9877265	337.000	-	0.000	-

CID9881652	262.857	0.090	595.250	0.048
CID9882105	244.760	-	0.000	-
CID9882189	195.000	0.639	181.000	<0.001
CID9883933	213.563	-	0.000	-
CID9895066	204.800	-	0.000	-
CID9907842	232.154	0.066	175.500	0.379
CID9909438	223.300	-	0.000	-
CID9910224	268.091	-	0.000	-
CID9911821	234.688	-	0.000	-
CID9913881	305.600	0.097	734.000	0.016
CID9914412	229.900	0.297	500.500	0.101
CID9915743	292.485	0.233	478.583	0.172
CID9918734	100.000	-	0.000	-
CID9926791	228.111	0.343	820.000	0.028
CID9927456	0.000	-	0.000	-
CID9930048	237.941	-	0.000	-
CID9931953	291.000	-	0.000	-
CID9933475	287.426	0.289	575.400	0.195
CID9934643	273.100	-	0.000	-
CID9937534	199.333	-	0.000	-
CID9939609	270.250	-	0.000	-
CID9949641	231.714	-	0.000	-
CID9952884	204.167	-	0.000	-
CID9953592	208.917	-	0.000	-
CID9955116	231.739	-	0.000	-
CID9956726	213.000	-	0.000	-
CID9962663	199.000	-	0.000	-
CID9977819	277.640	0.177	709.000	0.226
CID9978660	544.500	-	0.000	-
CID9982711	0.000	-	0.000	-

CID9987363	263.000	-	0.000	-
CID9988100	432.500	-	0.000	-
CID10010808	254.000	-	0.000	-
CID10012635	486.000	-	0.000	-
CID10016910	288.800	-	0.000	-
CID10022395	201.667	-	0.000	-
CID10026174	307.625	-	0.000	-
CID10027278	229.731	0.234	665.571	0.061
CID10044425	217.000	-	0.000	-
CID10056458	325.091	-	0.000	-
CID10062738	43.556	0.577	171.000	0.055
CID10062753	200.250	-	0.000	-
CID10070686	370.333	-	0.000	-
CID10074640	346.875	-	0.000	-
CID10079106	191.000	-	0.000	-
CID10096043	265.810	0.318	221.000	0.062
CID10113978	304.526	0.274	546.545	0.190
CID10126189	201.067	0.421	504.000	0.014
CID10127622	310.550	0.265	396.714	0.037
CID10138259	203.000	0.336	573.333	0.232
CID10154024	275.522	0.323	192.500	0.394
CID10158940	244.167	-	0.000	-
CID10163178	0.000	-	0.000	-
CID10172943	237.600	0.222	687.500	0.047
CID10218498	261.722	0.272	186.500	0.152
CID10255081	0.000	-	0.000	-
CID10282255	312.562	0.263	370.818	0.341
CID10302451	243.000	0.234	249.000	0.114
CID10317566	210.000	0.232	237.000	<0.001
CID10319891	232.500	0.095	334.000	0.009

CID10322450	313.895	0.108	326.000	0.049
CID10338254	319.000	-	0.000	-
CID10341154	227.636	-	0.000	-
CID10346993	457.333	-	0.000	-
CID10350612	489.000	-	0.000	-
CID10351178	176.000	-	0.000	-
CID10367662	291.444	-	0.000	-
CID10368812	195.333	-	0.000	-
CID10384072	221.750	0.237	658.000	0.112
CID10420128	179.800	-	0.000	-
CID10429020	253.462	-	0.000	-
CID10435195	239.667	-	0.000	-
CID10437018	250.700	0.365	895.500	0.006
CID10458325	277.238	0.180	190.000	0.011
CID10460379	310.722	0.256	424.000	0.171
CID10461815	301.609	0.152	496.000	0.143
CID10484730	303.000	-	0.000	-
CID10584166	178.333	-	0.000	-
CID10610417	185.500	-	0.000	-
CID10662822	0.000	-	0.000	-
CID10903498	378.000	-	0.000	-
CID10950581	224.500	-	0.000	-
CID10953556	155.286	0.203	211.000	0.022
CID10993999	167.000	-	0.000	-
CID11013287	129.778	-	0.000	-
CID11020241	315.194	0.409	325.538	0.428
CID11069089	256.400	-	0.000	-
CID11154925	224.533	0.261	466.000	0.023
CID11155655	233.000	-	0.000	-
CID11158972	235.667	-	0.000	-

CID11167602	277.786	0.219	425.000	0.064
CID11196273	262.221	0.351	240.900	0.158
CID11210478	412.200	0.061	611.600	0.059
CID11226090	200.545	-	0.000	-
CID11228183	254.059	0.307	439.125	0.118
CID11228257	208.333	-	0.000	-
CID11233655	216.000	-	0.000	-
CID11234052	311.167	0.196	417.000	0.103
CID11234794	248.333	-	0.000	-
CID11253490	189.000	-	0.000	-
CID11270518	442.500	-	0.000	-
CID11279826	243.000	-	0.000	-
CID11281905	233.000	-	0.000	-
CID11282283	254.889	0.174	461.000	0.011
CID11284169	198.250	-	0.000	-
CID11315214	281.667	-	0.000	-
CID11315474	202.857	-	0.000	-
CID11330431	0.000	-	0.000	-
CID11335394	225.095	-	0.000	-
CID11336170	249.500	-	0.000	-
CID11349170	262.571	0.168	823.000	<0.001
CID11350701	210.857	-	0.000	-
CID11353099	167.000	-	0.000	-
CID11354606	170.059	-	0.000	-
CID11354735	208.800	-	0.000	-
CID11363962	314.200	-	0.000	-
CID11364421	253.043	0.290	369.667	0.099
CID11375566	440.000	-	0.000	-
CID11399964	202.000	-	0.000	-
CID11404337	252.290	0.203	643.000	0.009

CID11411233	179.333	-	0.000	-
CID11414799	235.667	0.160	156.000	<0.001
CID11427824	336.000	-	0.000	-
CID11452147	484.000	-	0.000	-
CID11453337	0.000	-	0.000	-
CID11454753	181.000	-	0.000	-
CID11455973	286.778	-	0.000	-
CID11467166	204.182	-	0.000	-
CID11476171	286.583	-	0.000	-
CID11485656	251.391	0.291	487.000	0.224
CID11488320	276.800	-	0.000	-
CID11494412	324.556	0.149	413.333	0.011
CID11504294	255.857	-	0.000	-
CID11511120	302.176	0.131	576.000	0.025
CID11520587	263.000	-	0.000	-
CID11520894	240.188	0.270	170.500	0.026
CID11526696	208.500	-	0.000	-
CID11528249	192.857	-	0.000	-
CID11533060	319.000	-	0.000	-
CID11534420	256.063	0.288	327.000	0.086
CID11559546	264.500	-	0.000	-
CID11561674	228.400	-	0.000	-
CID11578515	296.871	0.193	849.600	0.008
CID11597571	0.000	-	739.800	-
CID11599950	247.875	0.105	159.000	<0.001
CID11611054	327.000	-	0.000	-
CID11639084	318.400	-	0.000	-
CID11640390	227.571	-	0.000	-
CID11646823	249.313	0.244	166.000	0.137
CID11647372	311.556	0.120	690.000	0.049

CID11655119	216.250	0.170	314.000	0.022
CID11661757	242.000	-	0.000	-
CID11667893	285.290	0.213	200.333	0.151
CID11671467	215.353	0.345	482.333	0.119
CID11672431	302.667	-	0.000	-
CID11675324	256.000	-	0.000	-
CID11679764	355.429	0.331	170.000	0.223
CID11707110	321.200	0.157	414.111	0.042
CID11713159	218.333	0.273	594.333	0.253
CID11714998	203.900	0.364	172.000	0.102
CID11717001	252.923	0.203	735.000	0.011
CID11738933	247.913	-	0.000	-
CID11758240	198.500	-	0.000	-
CID11822705	242.417	0.251	161.000	0.067
CID11849561	348.000	-	0.000	-
CID11953947	221.000	0.324	192.000	<0.001
CID11960529	296.222	-	0.000	-
CID11966249	263.750	0.110	177.000	0.266
CID11966311	320.036	<0.001	265.311	0.012
CID11969855	433.000	-	0.000	-
CID11969927	292.500	-	0.000	-
CID11969976	518.000	-	0.000	-
CID11970251	289.203	0.353	309.250	0.043
CID11977753	288.714	0.374	448.625	0.181
CID11979378	226.600	-	0.000	-
CID11984561	267.000	-	0.000	-
CID11984597	216.188	-	0.000	-
CID11987672	252.755	0.361	232.250	0.244
CID11987811	119.400	0.721	575.000	0.008
CID12007524	234.333	-	0.000	-

CID12083343	206.889	-	0.000	-
CID12123876	177.000	-	0.000	-
CID12149061	203.656	-	0.000	-
CID12210218	267.412	-	0.000	-
CID12295218	167.000	-	0.000	-
CID12339760	460.000	-	0.000	-
CID13174052	0.000	-	0.000	-
CID13559381	0.000	-	0.000	-
CID13715114	44.000	-	0.000	-
CID13783824	201.600	0.290	159.000	0.094
CID13829173	166.000	-	0.000	-
CID13868540	269.900	-	0.000	-
CID13891595	245.398	0.433	161.000	0.385
CID13899584	0.000	-	0.000	-
CID13922196	234.667	0.312	181.000	0.068
CID14009640	0.000	-	0.000	-
CID14143518	0.000	-	0.000	-
CID14476155	235.889	0.379	157.000	0.123
CID14518752	230.250	-	0.000	-
CID14797778	199.000	-	0.000	-
CID14942883	269.000	0.143	258.000	0.019
CID15167899	0.000	-	0.000	-
CID15461339	263.143	-	0.000	-
CID15547703	191.818	0.522	800.000	<0.001
CID15942416	0.000	-	0.000	-
CID15942656	241.214	-	0.000	-
CID15942688	205.000	-	0.000	-
CID15949377	214.000	-	0.000	-
CID15950351	297.667	0.042	613.000	<0.001
CID15950455	379.000	-	0.000	-

CID16007259	249.000	-	0.000	-
CID16019984	265.608	0.365	169.000	0.471
CID16038120	281.625	0.104	714.737	0.337
CID16049841	344.500	-	0.000	-
CID16061579	204.760	-	0.000	-
CID16070111	216.000	-	0.000	-
CID16072143	276.000	-	0.000	-
CID16090528	298.000	-	0.000	-
CID16090529	424.833	-	0.000	-
CID16090535	386.750	-	0.000	-
CID16090543	261.000	-	0.000	-
CID16090544	259.500	-	0.000	-
CID16098803	398.000	-	0.000	-
CID16098804	444.000	-	0.000	-
CID16098807	331.417	-	0.000	-
CID16098825	229.500	-	0.000	-
CID16098827	235.000	-	0.000	-
CID16098831	299.000	-	0.000	-
CID16115665	0.000	-	0.000	-
CID16122583	205.455	-	0.000	-
CID16129931	291.400	-	0.000	-
CID16129935	371.059	-	0.000	-
CID16129975	224.773	0.211	342.429	0.025
CID16130049	206.333	0.229	853.000	<0.001
CID16130072	338.778	-	0.000	-
CID16130904	241.333	-	0.000	-
CID16134956	290.895	0.217	199.000	0.241
CID16136245	218.913	-	0.000	-
CID16137271	216.050	-	0.000	-
CID16181638	257.271	0.446	159.500	0.458

CID16197683	0.000	-	0.000	-
CID16197723	200.286	0.477	151.000	0.038
CID16211699	0.000	-	0.000	-
CID16214793	207.929	-	0.000	-
CID16214946	362.400	-	0.000	-
CID16217590	263.250	0.213	192.000	0.270
CID16217673	218.167	-	0.000	-
CID16220188	194.000	-	0.000	-
CID16224745	210.000	-	0.000	-
CID16682876	332.136	-	0.000	-
CID16685274	304.649	-	0.000	-
CID16722832	243.900	-	0.000	-
CID16723044	332.500	-	0.000	-
CID16725726	229.462	0.267	756.000	0.103
CID16736529	252.286	0.299	160.500	0.042
CID16738509	355.833	-	0.000	-
CID16741197	51.667	-	0.000	-
CID16750073	245.052	0.650	221.000	0.207
CID16750408	279.333	-	0.000	-
CID16759369	498.667	0.026	441.000	0.018
CID16760281	228.157	0.344	266.500	0.150
CID16760627	258.417	0.189	705.222	0.309
CID16760673	187.000	-	0.000	-
CID16760674	0.000	-	0.000	-
CID16760691	270.000	0.052	814.000	<0.001
CID17749734	265.143	-	0.000	-
CID17754438	248.000	0.144	915.000	<0.001
CID17755020	382.000	-	0.000	-
CID17755023	302.500	-	0.000	-
CID17755025	447.667	-	0.000	-

CID17755052	363.125	0.101	598.583	0.061
CID17755122	232.500	-	0.000	-
CID17755126	545.333	-	0.000	-
CID17755409	196.000	-	0.000	-
CID17755517	420.667	-	0.000	-
CID17755622	198.667	-	0.000	-
CID17755624	207.500	-	0.000	-
CID17755638	348.667	-	0.000	-
CID17755735	467.750	-	0.000	-
CID17755739	336.000	-	0.000	-
CID17755743	417.000	-	0.000	-
CID17755745	273.000	-	0.000	-
CID17755847	413.333	-	0.000	-
CID17755849	283.500	-	0.000	-
CID17755854	259.500	-	0.000	-
CID17755972	469.000	-	0.000	-
CID17756083	387.500	-	0.000	-
CID17756087	451.000	-	0.000	-
CID17756089	247.000	-	0.000	-
CID17756199	324.500	-	0.000	-
CID17756600	377.800	-	0.000	-
CID17756796	220.333	-	0.000	-
CID17882607	244.625	-	0.000	-
CID17904999	204.333	-	0.000	-
CID18363976	279.500	-	0.000	-
CID18425712	321.700	-	0.000	-
CID18965963	215.800	-	0.000	-
CID18991124	201.045	0.439	168.000	0.100
CID20042692	900.000	0.008	808.739	<0.001
CID20055947	168.000	-	0.000	-

CID20620240	286.917	0.119	602.500	0.041
CID20715017	154.000	-	0.000	-
CID20835251	230.750	-	0.000	-
CID21673419	0.000	-	0.000	-
CID21674234	0.000	-	0.000	-
CID21697324	0.000	-	0.000	-
CID22024915	176.000	0.118	455.750	0.012
CID22137213	250.000	-	0.000	-
CID22243895	331.000	-	0.000	-
CID22451303	211.059	-	0.000	-
CID22608753	827.167	-	0.000	-
CID23582824	290.667	-	0.000	-
CID23624255	211.077	0.258	199.000	0.018
CID23635314	230.364	-	0.000	-
CID23635526	154.000	-	0.000	-
CID23635643	0.000	-	0.000	-
CID23653552	239.500	-	0.000	-
CID23661578	398.200	-	0.000	-
CID23668479	390.136	-	0.000	-
CID23674191	252.558	0.308	179.500	0.227
CID23690938	276.195	0.397	180.200	0.263
CID23724531	239.900	0.213	172.000	0.039
CID23725625	293.548	0.262	198.000	0.175
CID24180719	283.133	0.171	365.375	0.094
CID24199293	305.500	-	0.000	-
CID24200575	272.000	-	0.000	-
CID24202503	172.000	-	0.000	-
CID24202507	421.000	-	0.000	-
CID24202802	169.000	-	0.000	-
CID24202901	193.000	-	0.000	-

CID24202902	0.000	-	0.000	-
CID24202904	160.000	-	0.000	-
CID24202986	198.000	-	0.000	-
CID24203379	267.222	-	0.000	-
CID24203380	272.500	-	0.000	-
CID24203387	313.000	-	0.000	-
CID24203669	208.500	-	0.000	-
CID24204454	355.250	-	0.000	-
CID24205019	435.500	-	0.000	-
CID24205080	166.000	-	0.000	-
CID24205088	0.000	-	0.000	-
CID24205092	374.333	-	0.000	-
CID24205136	406.000	-	0.000	-
CID24205338	0.000	-	0.000	-
CID24205849	564.250	-	0.000	-
CID24205955	277.000	-	0.000	-
CID24728718	200.000	-	0.000	-
CID24739943	229.667	-	0.000	-
CID24756910	250.667	0.155	892.000	0.009
CID24764437	227.167	0.350	180.000	0.030
CID24764449	226.200	0.134	533.500	0.040
CID24771867	223.500	0.315	581.167	0.123
CID24776445	238.474	0.268	486.000	0.041
CID24779724	291.917	0.210	982.000	<0.001
CID24782986	184.000	-	0.000	-
CID24783227	267.621	0.184	253.000	0.023
CID24785538	234.222	0.210	556.667	0.037
CID24794418	341.167	0.061	822.000	0.011
CID24820119	266.600	-	0.000	-
CID24820122	0.000	-	172.000	-

CID24832036	567.000	-	0.000	-
CID24832095	222.679	-	0.000	-
CID24838940	251.357	0.184	170.000	0.207
CID24840378	330.697	0.167	916.000	<0.001
CID24847680	264.038	-	0.000	-
CID24847874	248.412	-	0.000	-
CID24856436	195.500	-	0.000	-
CID24892733	272.600	0.197	161.000	0.093
CID24901704	303.000	0.144	800.000	<0.001
CID24901722	307.933	0.155	260.000	0.041
CID24941245	256.640	0.184	182.000	0.045
CID24956525	274.593	0.274	345.667	0.020
CID24958200	256.143	-	0.000	-
CID24964624	264.560	0.311	589.800	0.053
CID24978514	308.379	0.112	241.175	0.162
CID24978538	249.742	0.271	750.000	0.022
CID24988881	261.000	-	0.000	-
CID25027363	321.333	0.122	246.500	0.080
CID25033539	503.500	0.058	835.000	<0.001
CID25076512	209.000	-	0.000	-
CID25081014	267.415	-	0.000	-
CID25087161	0.000	-	0.000	-
CID25088416	223.688	0.217	163.000	0.026
CID25102847	313.846	0.238	424.667	0.005
CID25113599	208.789	-	0.000	-
CID25126797	255.273	0.227	162.000	0.122
CID25167777	352.250	0.208	540.000	0.004
CID25171647	481.750	0.058	164.000	<0.001
CID25177719	209.875	-	0.000	-
CID25181561	273.357	-	0.000	-

CID25182616	233.125	0.193	417.778	0.171
CID25197651	202.500	-	0.000	-
CID25207668	224.800	-	0.000	-
CID26275995	282.581	0.392	461.800	0.161
CID27885548	312.346	0.200	445.519	0.135
CID28777137	267.619	0.164	318.000	0.141
CID28780578	0.000	-	0.000	-
CID42603598	188.667	-	0.000	-
CID42608440	201.783	-	0.000	-
CID42611257	340.147	0.227	339.611	0.099
CID42642645	323.783	0.104	698.222	0.291
CID44134894	305.049	0.148	328.208	0.067
CID44137945	274.133	0.150	823.000	0.010
CID44144423	233.000	-	0.000	-
CID44182395	409.333	0.074	209.000	0.030
CID44187362	266.833	-	0.000	-
CID44187953	273.167	0.164	754.500	0.015
CID44194024	201.333	-	0.000	-
CID44195570	269.833	-	0.000	-
CID44201342	200.778	-	0.000	-
CID44206063	260.667	-	0.000	-
CID44228987	430.429	0.093	234.333	0.054
CID44263835	305.342	0.150	247.887	0.097
CID44265722	0.000	-	0.000	-
CID44268108	300.456	0.157	457.118	0.136
CID44284466	386.000	-	0.000	-
CID44284481	300.876	0.213	296.353	0.261
CID44297792	386.000	-	0.000	-
CID44346852	0.000	-	0.000	-
CID44346870	343.000	-	0.000	-

CID44369013	354.714	-	0.000	-
CID44383484	420.000	-	0.000	-
CID44405214	412.000	-	0.000	-
CID44409870	197.000	-	0.000	-
CID44409873	494.000	-	0.000	-
CID44424639	301.000	-	0.000	-
CID44459146	202.000	-	0.000	-
CID44460192	292.020	0.307	428.091	0.304
CID44472890	206.745	0.318	154.000	0.249
CID44483210	217.055	0.234	170.200	0.094
CID44560005	216.286	-	0.000	-
CID44564107	220.727	-	0.000	-
CID44574681	212.000	-	0.000	-
CID44583974	185.000	-	0.000	-
CID44593851	224.590	0.257	270.000	0.016
CID44604932	194.471	0.344	185.000	0.070
CID44607530	401.067	0.090	690.333	0.019
CID44620954	165.056	-	0.000	-
CID44630480	181.667	-	0.000	-
CID44819697	345.000	-	0.000	-
CID45027806	411.500	-	0.000	-
CID45027831	510.667	-	0.000	-
CID45027834	375.000	-	0.000	-
CID45027835	348.000	-	0.000	-
CID45027837	185.000	-	0.000	-
CID45027839	189.000	-	0.000	-
CID45027842	243.500	-	0.000	-
CID45027851	186.000	-	0.000	-
CID45027853	261.500	-	0.000	-
CID45027874	247.500	-	0.000	-

CID45027885	198.000	-	0.000	-
CID45027893	325.000	-	0.000	-
CID45027980	356.429	-	0.000	-
CID45028149	313.800	-	0.000	-
CID45028394	0.000	-	0.000	-
CID45028539	0.000	-	0.000	-
CID45028841	214.000	-	0.000	-
CID45029440	181.000	-	0.000	-
CID45100465	139.909	-	0.000	-
CID45100498	265.600	0.172	184.000	0.096
CID45142457	230.000	0.036	206.000	<0.001
CID45259048	540.000	-	0.000	-
CID45270897	282.280	0.213	507.000	0.055
CID45280987	237.500	-	0.000	-
CID45382213	198.000	0.233	159.000	0.023
CID45480163	227.500	0.328	234.111	0.122
CID46173852	221.000	-	0.000	-
CID46216795	238.200	0.278	262.000	0.034
CID46238531	180.182	0.592	206.200	0.089
CID46738086	0.000	-	0.000	-
CID46780173	0.000	-	0.000	-
CID46885626	541.000	0.022	704.500	0.045
CID46898058	187.333	-	0.000	-
CID46911863	305.667	0.203	182.000	0.027
CID46931226	254.375	-	0.000	-
CID49769060	252.600	0.301	205.500	0.182
CID49769423	169.000	0.131	190.000	0.029
CID49770639	0.000	-	0.000	-
CID49773920	245.000	-	0.000	-
CID49786966	287.333	-	0.000	-

CID49837887	213.000	0.127	900.000	<0.001
CID49852665	327.800	-	0.000	-
CID49867926	257.600	0.368	207.000	0.008
CID49867930	238.909	-	0.000	-
CID51033073	220.000	-	0.000	-
CID51346199	217.000	0.264	220.000	0.195
CID51351607	166.000	-	0.000	-
CID51529148	369.053	-	0.000	-
CID51614528	178.000	-	0.000	-
CID52945214	281.500	-	0.000	-
CID52948907	232.000	-	0.000	-
CID52950079	375.000	-	0.000	-
CID53239748	233.756	-	0.000	-
CID53297359	243.000	-	0.000	-
CID53297363	227.250	-	0.000	-
CID53316630	168.500	-	0.000	-
CID53320414	166.182	-	0.000	-
CID53346506	221.552	0.557	272.571	0.172
CID53346509	179.667	-	0.000	-
CID53346510	218.391	0.462	241.333	0.193
CID53346511	228.481	0.430	215.500	0.303
CID53352191	189.867	0.426	193.667	0.123
CID53361478	215.667	-	0.000	-
CID53384665	222.974	0.269	152.000	0.134
CID53427792	261.474	0.443	199.250	0.157
CID53470395	215.267	-	0.000	-
CID53477714	306.088	0.165	473.586	0.161
CID53477911	289.675	0.033	201.667	0.489
CID53488435	88.294	-	0.000	-
CID53627507	217.276	-	0.000	-

CID53627540	108.556	-	0.000	-
CID53627975	186.500	-	0.000	-
CID53627990	316.071	-	0.000	-
CID53628752	225.500	-	0.000	-
CID53629012	264.143	-	0.000	-
CID53629505	317.439	0.212	461.000	0.223
CID53629594	201.333	-	0.000	-
CID53630250	266.000	-	0.000	-
CID53630776	200.500	0.203	180.000	<0.001
CID53630877	251.029	0.265	215.000	0.032
CID54514979	393.000	-	0.000	-
CID54600732	264.000	-	0.000	-
CID54600749	437.667	-	0.000	-
CID54601571	326.857	-	0.000	-
CID54601897	370.000	-	0.000	-
CID54604163	190.000	-	0.000	-
CID54604312	327.500	-	0.000	-
CID54604600	381.800	-	0.000	-
CID54607189	253.000	-	0.000	-
CID54607693	337.400	-	0.000	-
CID54608402	374.500	-	0.000	-
CID54608404	395.000	-	0.000	-
CID54608406	396.571	-	0.000	-
CID54608408	376.429	-	0.000	-
CID54608410	386.714	-	0.000	-
CID54608412	339.167	-	0.000	-
CID54608414	362.800	-	0.000	-
CID54608425	349.429	-	0.000	-
CID54608427	422.444	-	0.000	-
CID54608429	369.714	-	0.000	-

CID54608484	244.600	-	0.000	-
CID54608726	423.000	-	0.000	-
CID54608728	311.286	-	0.000	-
CID54608765	187.000	-	0.000	-
CID54608835	386.400	-	0.000	-
CID54608935	282.200	-	0.000	-
CID54608937	339.000	-	0.000	-
CID54608939	346.000	-	0.000	-
CID54609526	282.538	0.184	154.000	0.175
CID54612586	235.000	-	0.000	-
CID54612587	311.333	-	0.000	-
CID54612640	325.750	-	0.000	-
CID54612825	299.000	-	0.000	-
CID54612932	0.000	-	0.000	-
CID54613300	0.000	-	0.000	-
CID54613553	211.000	-	0.000	-
CID54671203	306.564	0.265	500.909	0.323
CID54680692	273.906	-	0.000	-
CID54684141	326.214	0.202	523.600	0.072
CID54684286	257.375	-	0.000	-
CID54686054	209.333	-	0.000	-
CID54693691	205.375	0.596	227.667	0.054
CID54710406	252.583	0.084	700.000	<0.001
CID54716343	214.667	-	0.000	-
CID54734719	269.979	0.312	235.833	0.058
CID54746910	166.000	-	0.000	-
CID54751306	184.000	-	0.000	-
CID56603655	255.479	0.367	397.000	<0.001
CID56603668	265.857	0.083	329.250	0.006
CID56603704	195.000	-	0.000	-

CID56649450	484.400	0.120	199.000	<0.001
CID56663890	286.000	-	0.000	-
CID56671814	0.000	-	798.000	-
CID56684138	248.069	0.117	159.000	0.185
CID56835777	228.596	-	0.000	-
CID56841630	291.125	-	0.000	-
CID56841665	276.385	-	0.000	-
CID56841667	315.400	-	0.000	-
CID56841714	375.188	-	0.000	-
CID56841756	314.556	-	0.000	-
CID56841764	320.250	0.122	162.000	0.008
CID56841800	219.692	0.276	167.000	0.023
CID56841866	190.615	-	0.000	-
CID56841932	212.125	-	0.000	-
CID56841944	209.222	-	0.000	-
CID56841950	314.667	-	0.000	-
CID56841986	102.800	-	0.000	-
CID56841998	284.833	-	0.000	-
CID56841999	311.154	0.092	288.000	<0.001
CID56842042	333.292	0.168	262.000	0.008
CID56842080	218.714	-	0.000	-
CID56842082	331.000	0.088	186.000	0.011
CID56842117	327.128	0.275	271.500	0.045
CID56842118	190.286	0.430	182.000	0.004
CID56842121	328.571	0.277	310.000	0.054
CID56842148	203.182	-	0.000	-
CID56842156	236.571	-	0.000	-
CID56842157	418.652	0.105	205.000	0.022
CID56842159	376.833	-	0.000	-
CID56842161	248.286	-	0.000	-

CID56842163	277.857	-	0.000	-
CID56842198	272.143	-	0.000	-
CID56842358	121.000	-	0.000	-
CID56843240	246.625	0.220	171.000	0.041
CID56951714	260.041	-	0.000	-
CID56952026	238.667	0.102	183.000	<0.001
CID57325589	267.429	-	0.000	-
CID57326580	277.111	-	0.000	-
CID57339136	203.000	-	0.000	-
CID57339833	257.000	-	0.000	-
CID57372617	174.500	-	0.000	-
CID57390074	286.333	0.085	237.500	0.005
CID57391679	156.667	-	0.000	-
CID58783103	171.000	-	0.000	-
CID59218642	200.500	-	0.000	-
CID59229792	466.500	-	0.000	-
CID59744451	167.000	-	0.000	-
CID60147041	0.000	-	900.000	-
CID60147953	364.000	-	0.000	-
CID60147955	169.000	-	0.000	-
CID60148305	394.500	-	0.000	-
CID60148441	282.500	-	0.000	-
CID60150360	322.308	-	0.000	-
CID60677956	0.000	-	0.000	-
CID63321115	0.000	-	0.000	-
CID66553073	242.200	0.159	154.000	0.037
CID66553115	231.143	-	0.000	-
CID66553121	222.000	-	0.000	-
CID66577011	213.800	-	0.000	-
CID66577045	131.000	-	0.000	-

CID68776950	0.000	-	0.000	-
CID70679302	188.941	0.371	161.000	0.120
CID70680240	217.795	0.337	243.000	0.147
CID70680574	214.262	-	0.000	-
CID70683012	190.909	-	0.000	-
CID70683023	228.273	0.374	182.000	0.147
CID70687253	207.250	-	0.000	-
CID70687308	206.200	-	0.000	-
CID70689314	209.846	-	0.000	-
CID70689355	204.231	-	0.000	-
CID70693549	188.846	-	0.000	-
CID70695615	200.484	-	0.000	-
CID70695618	209.222	-	0.000	-
CID70702308	28.667	-	0.000	-
CID70789251	204.000	-	0.000	-
CID71296049	216.093	-	0.000	-
CID71296083	376.000	-	0.000	-
CID71297189	193.667	0.430	252.000	<0.001
CID71300601	190.538	-	0.000	-
CID71300725	175.000	-	0.000	-
CID71301280	196.500	-	0.000	-
CID71301286	205.000	-	0.000	-
CID71308162	288.182	0.091	447.000	0.052
CID71311919	274.031	0.348	186.667	0.326
CID71316700	210.714	-	0.000	-
CID71463576	188.815	0.327	218.750	0.034
CID71464519	225.941	0.211	218.500	0.251
