# Supplementary material for: A computational method for the identification of candidate drugs for non-small cell lung cancer
Source: PLoS One. 2017 Aug 18;12(8):e0183411. doi: 10.1371/journal.pone.0183411 (PMC5562320; doi:10.1371/journal.pone.0183411)
Supplement: S4 Table — (PDF) [file pone.0183411.s004.pdf]

S4 Table. 1007 candidate compounds filtered by the association test and permutation test

PubChem ID	Rating score on NSCLC-related chemicals	P-value on NSCLC-related chemicals	Rating score on NSCLC-related genes	P-value on NSCLC-related genes
CID6	306.358	0.310	385.500	0.199
CID15	253.353	0.258	759.500	0.036
CID137	291.034	0.173	298.500	0.163
CID138	249.202	0.256	190.333	0.159
CID143	350.698	0.377	265.667	0.156
CID174	290.464	0.144	218.750	0.263
CID180	296.502	0.134	246.773	0.174
CID190	333.474	0.358	340.800	0.185
CID196	244.926	0.220	199.000	0.062
CID206	310.689	0.022	248.000	0.193
CID237	267.277	0.252	730.200	0.036
CID241	296.193	0.093	348.500	0.040
CID271	424.655	0.460	627.338	0.023
CID311	376.889	0.434	561.125	0.110
CID338	285.161	0.336	583.375	0.113
CID402	334.274	0.220	401.154	0.196
CID444	311.539	0.340	743.667	0.121
CID597	327.565	0.228	249.471	0.242
CID602	305.881	0.033	316.800	0.240
CID611	325.504	0.013	363.750	0.206
CID612	307.221	0.080	304.000	0.238
CID702	334.806	0.012	388.081	0.103
CID712	333.645	0.190	299.076	0.041
CID743	277.753	0.373	180.000	0.266

CID764	327.154	0.316	351.034	0.131
CID778	333.167	0.292	599.250	0.150
CID787	287.667	0.436	248.000	0.102
CID790	325.212	0.452	511.000	0.090
CID836	267.240	0.360	161.000	0.277
CID857	311.879	0.100	222.500	0.274
CID876	326.655	0.060	233.621	0.146
CID887	315.095	0.103	269.953	0.271
CID892	327.088	0.365	523.875	0.115
CID996	308.018	0.204	305.833	0.236
CID1032	320.422	0.123	681.222	0.253
CID1048	258.319	0.193	520.500	0.054
CID1049	281.820	0.128	363.833	0.061
CID1060	561.437	0.444	668.000	0.258
CID1099	322.612	0.398	259.750	0.209
CID1112	469.578	0.114	187.000	0.201
CID1130	347.226	0.261	497.500	0.041
CID1135	333.043	0.187	245.667	0.254
CID1140	300.429	0.096	640.250	0.040
CID1153	359.118	0.292	252.000	<0.001
CID1175	317.330	0.434	606.000	0.075
CID1188	310.168	0.359	397.833	0.132
CID1401	300.697	0.188	551.400	0.100
CID1464	269.787	0.388	253.692	0.112
CID1493	248.102	0.377	169.500	0.191
CID1674	289.172	0.180	324.923	0.242
CID1697	173.000	0.247	461.000	0.006
CID1775	308.954	0.256	396.556	0.210
CID1923	265.765	0.190	150.000	0.240
CID1983	306.034	0.252	279.276	<0.001

CID2007	272.853	0.248	165.000	0.143
CID2016	229.175	0.343	245.000	0.055
CID2022	275.344	0.230	223.667	0.248
CID2044	249.500	0.140	475.500	0.034
CID2051	235.368	0.210	573.250	0.086
CID2088	291.522	0.303	504.000	0.113
CID2092	260.340	0.349	319.500	0.131
CID2094	272.293	0.264	486.000	0.133
CID2141	278.414	0.274	466.400	0.055
CID2148	278.875	0.341	489.000	0.093
CID2156	307.105	0.318	245.400	0.257
CID2160	341.995	0.224	784.500	0.203
CID2179	299.783	0.231	605.750	0.060
CID2187	285.204	0.314	239.500	0.122
CID2202	262.568	0.438	800.000	0.012
CID2244	318.857	0.090	417.700	0.168
CID2265	302.520	0.260	427.000	0.099
CID2336	308.447	0.165	296.267	<0.001
CID2349	308.180	0.088	254.200	0.136
CID2375	310.922	0.283	448.625	0.151
CID2380	301.225	0.445	181.000	0.269
CID2459	212.591	0.375	168.500	0.173
CID2475	235.902	0.339	161.000	0.292
CID2478	342.694	0.253	496.500	0.071
CID2519	296.244	0.190	501.000	0.145
CID2554	325.163	0.203	499.000	0.166
CID2577	351.615	0.130	303.000	0.021
CID2578	321.917	0.249	409.267	0.103
CID2662	291.797	0.310	535.407	0.263
CID2708	321.676	0.258	414.333	0.099

CID2719	299.629	0.261	447.778	0.211
CID2733	257.504	0.338	585.000	0.082
CID2746	364.000	0.148	188.333	0.103
CID2756	305.100	0.278	780.750	0.148
CID2764	305.654	0.350	408.667	0.113
CID2767	318.515	0.220	466.587	0.278
CID2770	284.048	0.225	800.000	<0.001
CID2776	281.421	0.149	658.250	0.037
CID2794	321.857	0.212	908.000	<0.001
CID2907	328.268	0.168	315.323	0.227
CID2955	278.562	0.311	426.750	0.138
CID3016	321.593	0.101	781.333	0.128
CID3032	304.273	0.271	458.889	0.276
CID3071	240.754	0.227	479.000	0.071
CID3082	267.809	0.275	152.000	0.197
CID3108	286.069	0.329	792.250	0.083
CID3121	287.467	0.145	296.875	0.142
CID3152	245.526	0.369	702.333	0.067
CID3213	261.917	0.360	654.500	0.097
CID3283	287.186	0.063	248.750	0.220
CID3300	224.286	0.244	175.000	0.035
CID3305	266.726	0.433	159.000	0.230
CID3307	208.333	0.336	168.000	0.146
CID3308	278.273	0.297	750.000	0.062
CID3347	268.266	0.336	334.250	0.067
CID3348	275.471	0.363	778.333	0.064
CID3365	273.880	0.379	509.000	0.054
CID3366	330.140	0.237	186.000	0.127
CID3385	327.543	0.291	541.762	0.049
CID3394	285.667	0.317	728.333	0.044

CID3397	293.664	0.289	475.000	0.151
CID3414	298.589	0.360	239.000	0.085
CID3440	290.283	0.214	388.667	0.266
CID3446	305.147	0.225	750.000	0.060
CID3454	268.158	0.319	202.000	0.241
CID3467	310.133	0.138	322.850	0.103
CID3468	268.416	0.408	233.273	0.275
CID3503	256.080	0.462	453.000	0.080
CID3589	283.922	0.242	498.500	0.057
CID3624	291.666	0.276	238.784	0.187
CID3652	318.766	0.251	184.000	0.210
CID3657	320.241	0.212	393.778	0.108
CID3672	273.017	0.152	403.667	0.272
CID3676	326.569	0.204	547.600	0.172
CID3690	350.052	0.317	373.250	0.131
CID3715	316.876	0.218	581.037	0.123
CID3767	285.699	0.316	467.250	0.073
CID3883	262.923	0.326	366.667	0.132
CID3899	349.884	0.212	565.000	0.067
CID3902	338.446	0.246	370.667	0.112
CID3922	301.303	0.223	641.000	0.057
CID3950	339.815	0.310	334.000	0.035
CID3954	255.653	0.380	906.000	0.016
CID3958	312.766	0.257	168.000	0.202
CID4075	263.033	0.272	503.500	0.169
CID4095	273.608	0.289	595.500	0.107
CID4156	313.143	0.251	296.000	0.188
CID4169	283.422	0.277	548.667	0.041
CID4173	310.074	0.364	247.750	0.139
CID4189	291.594	0.313	852.000	0.045

CID4211	329.481	0.209	166.000	0.163
CID4212	343.892	0.182	472.429	0.189
CID4261	355.283	0.208	541.625	0.147
CID4485	317.994	0.189	482.222	0.264
CID4495	276.931	0.270	321.000	0.189
CID4553	332.195	0.289	530.286	0.189
CID4578	293.581	0.289	180.000	0.114
CID4594	265.615	0.260	355.500	0.196
CID4595	289.781	0.462	491.000	0.134
CID4604	352.840	0.162	207.000	0.069
CID4609	297.015	0.268	585.727	0.081
CID4673	321.653	0.307	409.333	0.137
CID4679	262.324	0.317	852.000	0.037
CID4705	261.303	0.164	563.875	0.100
CID4707	265.136	0.183	708.000	0.031
CID4708	263.652	0.133	663.000	0.031
CID4740	253.064	0.311	552.000	0.174
CID4763	306.258	0.168	582.091	0.137
CID4766	272.679	0.377	224.706	0.169
CID4784	298.201	0.338	228.622	0.263
CID4873	325.646	0.038	259.829	0.249
CID4909	306.177	0.330	244.000	0.194
CID4911	264.389	0.318	510.500	0.229
CID4913	333.205	0.274	206.000	0.220
CID4915	363.500	0.236	247.333	0.068
CID4939	287.644	0.362	272.564	0.144
CID4946	343.336	0.072	472.625	0.252
CID4971	347.889	0.326	437.000	0.115
CID4973	463.655	0.253	809.500	0.287
CID4993	314.976	0.411	636.800	0.074

CID4996	378.167	0.140	163.000	0.138
CID5035	320.887	0.332	410.048	0.193
CID5070	245.032	0.370	598.000	0.145
CID5090	285.919	0.293	300.600	0.162
CID5213	238.459	0.268	826.111	0.038
CID5215	313.325	0.289	811.000	<0.001
CID5234	340.201	<0.001	259.243	0.183
CID5235	255.372	0.360	321.650	0.076
CID5245	306.562	0.291	527.000	0.061
CID5311	324.202	0.198	458.407	0.117
CID5333	276.211	0.312	376.000	0.133
CID5342	269.862	0.227	831.000	0.038
CID5344	283.276	0.314	161.000	0.289
CID5345	295.828	0.295	533.500	0.060
CID5381	348.818	0.145	680.000	0.084
CID5394	277.094	0.393	318.684	0.226
CID5426	299.323	0.303	464.053	0.274
CID5453	319.816	0.218	352.750	0.046
CID5472	255.990	0.386	819.000	0.085
CID5546	250.221	0.408	279.000	0.178
CID5578	291.426	0.330	313.000	0.098
CID5590	309.873	0.149	263.065	0.091
CID5641	278.162	0.250	467.250	0.064
CID5744	200.916	0.333	171.500	0.177
CID5746	313.781	0.169	439.167	0.149
CID5755	289.768	0.130	327.800	0.245
CID5757	333.304	0.096	532.537	<0.001
CID5760	219.881	0.247	169.500	0.273
CID5768	301.043	0.224	660.333	0.071
CID5770	284.394	0.176	418.667	0.213

CID5790	280.625	0.179	204.000	0.210
CID5793	359.467	0.001	317.788	0.050
CID5798	294.149	0.166	909.000	<0.001
CID5819	311.962	0.438	468.333	0.211
CID5825	309.393	0.131	164.000	0.277
CID5833	304.884	0.332	473.600	0.230
CID5834	205.029	0.407	800.000	<0.001
CID5865	300.091	0.154	299.143	0.221
CID5897	281.979	0.230	537.000	0.037
CID5904	303.229	0.299	409.000	0.162
CID5905	224.300	0.278	151.000	0.282
CID5920	318.995	0.402	441.417	0.249
CID5954	326.898	0.165	275.200	0.057
CID5955	258.239	0.336	444.400	0.005
CID5959	320.743	0.191	295.600	0.122
CID5978	303.750	0.216	819.000	<0.001
CID5984	300.022	0.072	286.600	0.253
CID5994	320.423	0.063	529.400	0.011
CID6006	336.745	0.321	196.222	0.253
CID6013	314.375	0.096	357.484	0.121
CID6035	278.650	0.249	234.864	0.271
CID6047	344.549	0.321	374.800	0.272
CID6049	337.030	0.006	274.413	0.151
CID6058	299.953	0.237	341.000	0.144
CID6076	324.099	0.030	487.591	0.257
CID6104	277.393	0.244	180.000	0.029
CID6115	282.312	0.123	534.000	0.028
CID6163	315.111	0.097	150.000	0.061
CID6197	305.042	0.177	441.861	0.219
CID6199	279.414	0.330	205.000	0.216

CID6212	296.961	0.119	256.848	0.133
CID6249	321.416	0.130	244.667	0.259
CID6251	294.536	0.338	397.500	0.167
CID6252	320.799	0.178	257.111	0.169
CID6256	258.393	0.305	522.667	0.048
CID6279	299.076	0.143	442.600	0.087
CID6325	293.313	0.231	288.750	0.085
CID6338	274.964	0.278	886.667	0.006
CID6421	294.067	0.229	255.000	0.203
CID6436	322.987	0.213	407.000	0.027
CID6503	349.595	0.020	300.659	0.119
CID6508	276.395	0.264	176.000	0.180
CID6579	286.215	0.299	268.300	0.191
CID6675	333.714	0.356	313.667	0.228
CID6741	276.498	0.206	618.400	0.045
CID6742	164.429	0.345	170.429	0.086
CID6802	308.678	0.332	397.125	0.103
CID7099	243.250	0.363	181.000	0.046
CID7187	257.913	0.110	909.000	0.011
CID7847	278.169	0.188	550.000	0.264
CID7950	224.158	0.303	288.000	<0.001
CID8378	286.779	0.176	295.565	0.175
CID8549	275.004	0.440	434.571	0.136
CID8778	305.049	0.141	248.000	0.193
CID8977	773.765	0.347	873.000	0.137
CID8987	270.324	0.242	540.000	0.074
CID9033	273.761	0.236	296.500	0.056
CID9048	398.241	0.214	259.000	0.085
CID9062	256.322	0.297	520.500	0.111
CID9215	237.239	0.390	203.500	0.047

CID9250	293.565	0.233	458.000	0.046
CID9260	286.730	0.189	247.857	0.127
CID9444	292.630	0.385	400.391	0.284
CID9679	282.741	0.339	197.000	0.250
CID9782	263.130	0.262	380.000	0.120
CID9864	285.337	0.254	822.000	0.008
CID10340	301.277	0.099	272.429	0.134
CID10413	290.507	0.091	180.000	0.261
CID10430	266.242	0.125	161.000	0.172
CID10457	261.125	0.166	169.000	0.100
CID10465	313.863	0.023	551.000	<0.001
CID10635	313.634	0.122	419.875	0.183
CID10786	271.450	0.104	179.667	0.240
CID10964	283.525	0.224	290.000	0.178
CID11103	309.920	0.225	224.333	0.029
CID11178	84.600	0.320	187.000	0.003
CID11254	329.250	0.205	493.600	0.076
CID11266	286.151	0.152	196.000	0.089
CID12137	224.485	0.308	178.750	0.152
CID12560	299.599	0.195	533.000	0.047
CID12660	265.855	0.285	192.667	0.146
CID12699	279.068	0.199	313.167	0.091
CID12733	284.765	0.138	381.000	0.123
CID12736	200.875	0.299	186.000	0.038
CID12967	256.846	0.297	286.400	0.076
CID13116	281.200	0.240	409.000	0.028
CID13342	296.786	0.311	437.000	0.161
CID13588	264.302	0.200	264.857	0.093
CID13698	294.172	0.313	220.476	0.161
CID13711	334.768	0.235	262.313	0.234

CID13945	386.333	0.275	924.000	0.032
CID14227	238.568	0.450	230.000	0.141
CID14457	253.589	0.093	179.000	0.131
CID14797	289.761	0.112	225.333	0.192
CID14888	266.058	0.210	419.790	0.005
CID14932	208.300	0.389	237.000	0.032
CID14941	245.274	0.370	231.000	0.097
CID15032	306.902	0.194	209.000	0.181
CID16834	245.911	0.260	592.667	0.050
CID16850	279.125	0.326	229.000	0.279
CID17513	287.600	0.236	199.000	0.146
CID18343	323.970	0.213	605.800	0.238
CID18407	72.125	0.390	221.000	0.028
CID19001	273.818	0.348	184.071	0.187
CID19261	185.853	0.229	150.000	0.028
CID20279	290.897	0.212	614.667	0.115
CID20469	372.286	0.174	516.667	0.114
CID21157	271.133	0.289	506.067	0.130
CID21672	182.588	0.385	190.000	0.011
CID21704	241.347	0.275	203.000	0.087
CID22318	294.118	0.258	683.500	0.093
CID22469	304.596	0.237	811.000	<0.001
CID22986	205.000	0.108	156.000	<0.001
CID23267	265.500	0.185	178.500	0.203
CID23424	264.409	0.173	180.000	0.019
CID23830	332.044	0.297	314.451	0.186
CID23939	302.862	0.321	316.667	0.238
CID23963	278.286	0.297	508.000	0.051
CID23968	270.636	0.384	204.727	0.243
CID23991	320.708	0.388	527.000	0.096

CID24083	300.596	0.170	262.000	0.172
CID24261	290.960	0.097	373.615	0.170
CID24360	288.281	0.342	446.250	0.168
CID24441	302.204	0.253	233.550	0.222
CID24450	281.876	0.256	306.600	0.051
CID24529	312.844	0.455	320.375	0.124
CID24538	304.736	0.153	254.667	0.180
CID24584	315.423	0.055	239.741	0.121
CID24632	287.889	0.229	295.000	0.099
CID24748	239.429	0.107	199.000	0.136
CID24759	254.264	0.229	361.625	0.187
CID24763	278.561	0.244	326.000	0.050
CID24769	283.376	0.318	188.500	0.233
CID24775	311.700	0.246	245.680	0.148
CID24854	320.559	0.066	236.792	0.194
CID25419	264.000	0.311	298.800	0.049
CID25473	244.302	0.243	700.000	<0.001
CID26105	310.606	0.382	167.000	0.026
CID26879	275.403	0.434	196.800	0.272
CID27287	343.281	0.154	180.000	0.229
CID27337	248.294	0.266	413.000	0.018
CID28486	307.187	0.186	572.375	0.096
CID29029	358.000	0.254	434.000	0.017
CID29327	280.048	0.297	483.267	0.151
CID30323	311.747	0.158	391.273	0.266
CID30623	282.760	0.267	150.000	0.109
CID30751	225.277	0.293	800.000	0.013
CID31083	289.389	0.119	183.000	0.158
CID31254	253.118	0.132	700.000	<0.001
CID31307	309.679	0.235	512.000	0.087

CID31401	283.623	0.254	657.400	0.159
CID31402	323.372	0.196	724.000	0.014
CID31703	317.730	0.104	569.902	0.136
CID32874	275.593	0.185	181.000	<0.001
CID33113	409.573	0.437	729.364	0.025
CID33557	280.844	0.340	240.000	0.229
CID33576	288.586	0.230	244.500	0.203
CID33613	331.319	0.261	525.500	0.033
CID33641	355.857	0.087	700.000	<0.001
CID33776	285.886	0.257	491.750	0.019
CID34192	208.037	0.411	192.000	0.018
CID34457	327.727	0.127	237.000	0.005
CID35370	267.172	0.278	439.800	0.170
CID36294	317.234	0.311	221.333	0.149
CID36462	323.811	0.169	554.395	0.171
CID36797	219.188	0.403	190.000	0.057
CID37542	258.397	0.448	379.500	0.183
CID37768	337.519	0.280	230.000	0.122
CID38003	264.667	0.198	181.500	0.197
CID38347	322.628	0.238	295.600	0.069
CID38777	366.850	0.379	282.000	0.032
CID38852	278.991	0.217	253.500	0.130
CID39186	291.861	0.322	882.500	0.034
CID39214	330.649	0.273	150.000	0.054
CID39562	276.310	0.214	706.500	0.020
CID39981	351.800	0.180	161.000	0.090
CID40772	234.000	0.239	204.000	0.224
CID40839	365.093	0.207	186.667	0.071
CID41867	342.417	0.219	428.357	0.121
CID42616	298.763	0.168	970.000	<0.001

CID42890	352.155	0.199	296.000	0.094
CID43805	453.353	0.198	800.000	0.024
CID44093	319.786	0.231	491.167	0.176
CID47326	262.620	0.235	182.000	0.077
CID47938	209.077	0.189	268.000	0.006
CID49561	220.688	0.431	173.000	0.040
CID50515	242.167	0.239	469.000	<0.001
CID50599	308.685	0.233	194.000	0.113
CID50981	255.118	0.303	209.000	0.043
CID53232	265.680	0.351	661.435	0.192
CID54454	285.955	0.303	620.219	0.172
CID54687	295.845	0.309	806.250	0.145
CID54886	232.261	0.443	179.000	0.133
CID55245	314.255	0.269	424.409	0.281
CID55283	297.946	0.336	871.667	0.009
CID55466	290.937	0.377	239.000	0.044
CID57469	317.326	0.265	700.000	0.107
CID60198	264.094	0.242	376.750	0.064
CID60606	283.597	0.419	617.500	0.092
CID60613	294.205	0.381	194.000	0.077
CID60699	303.375	0.343	731.222	0.065
CID60749	320.739	0.228	499.838	0.126
CID60779	306.804	0.317	291.867	0.069
CID60795	318.933	0.170	344.000	0.246
CID60808	268.429	0.204	800.000	<0.001
CID60825	330.228	0.315	408.333	0.128
CID60837	309.574	0.310	439.694	0.244
CID60852	307.756	0.246	262.000	0.121
CID60934	300.701	0.306	836.500	0.026
CID60953	312.059	0.302	370.500	0.265

CID60955	225.632	0.272	230.000	0.025
CID61565	309.000	0.081	200.500	0.015
CID61635	229.955	0.332	299.750	0.063
CID61671	311.780	0.332	259.634	0.235
CID62210	231.867	0.193	150.000	0.111
CID64142	298.541	0.291	699.200	0.146
CID64730	250.123	0.083	192.000	0.146
CID64968	480.231	0.128	904.500	0.050
CID65005	239.206	0.282	187.000	0.024
CID65041	293.094	0.167	190.500	0.259
CID65063	483.457	0.375	274.000	0.221
CID65064	284.911	0.224	550.579	0.162
CID65091	384.511	0.407	921.000	0.019
CID65106	266.353	0.431	179.000	0.029
CID65110	309.773	0.428	347.333	0.159
CID65191	230.931	0.409	173.000	0.072
CID65217	278.847	0.312	334.200	0.017
CID65237	295.806	0.233	162.000	0.037
CID65253	279.917	0.461	182.000	0.143
CID65275	299.222	0.306	242.909	0.109
CID65407	336.140	0.221	207.000	0.259
CID65628	322.027	0.251	536.200	0.049
CID65948	259.542	0.141	800.000	<0.001
CID65958	230.623	0.430	383.333	0.045
CID66868	305.831	0.305	360.000	0.117
CID67431	283.667	0.187	161.000	0.260
CID67491	287.417	0.213	196.000	0.012
CID68152	283.320	0.255	238.318	0.197
CID68329	196.000	0.374	258.000	0.024
CID68740	321.326	0.329	392.167	0.076

CID68770	234.154	0.263	937.000	<0.001
CID69435	236.926	0.325	900.000	<0.001
CID71068	264.869	0.397	184.625	0.227
CID71184	259.852	0.389	264.000	0.029
CID71398	148.500	0.176	183.000	0.124
CID71563	277.077	0.231	900.000	<0.001
CID71616	249.340	0.391	166.000	0.263
CID71741	312.227	0.249	180.000	<0.001
CID72271	278.607	0.227	452.810	0.211
CID72402	281.895	0.401	160.500	0.090
CID72435	252.333	0.266	345.667	0.028
CID72571	239.667	0.221	158.000	0.017
CID73009	222.143	0.252	196.000	0.029
CID73212	275.718	0.249	343.913	0.016
CID74989	287.160	0.438	227.000	0.193
CID75142	271.649	0.321	307.957	0.171
CID80170	258.471	0.342	158.000	0.154
CID82146	277.288	0.227	591.615	0.129
CID83970	279.647	0.221	181.000	0.025
CID84029	290.380	0.262	279.000	0.144
CID84691	252.125	0.320	336.000	0.050
CID84759	290.444	0.247	182.000	<0.001
CID86222	218.400	0.247	181.000	0.016
CID89105	385.576	0.181	930.000	0.010
CID89594	313.525	0.316	496.472	0.200
CID91482	190.208	0.349	159.000	0.265
CID92242	299.286	0.282	170.333	0.041
CID92727	290.460	0.307	307.833	0.109
CID92787	324.221	0.229	414.000	0.075
CID93004	301.000	0.096	545.571	0.058

CID93577	253.958	0.147	191.000	0.058
CID93860	272.500	0.104	151.000	0.170
CID94312	249.000	0.411	164.500	0.280
CID100153	352.467	0.191	181.000	0.019
CID100154	253.474	0.353	161.000	0.072
CID100427	324.750	0.216	165.000	0.022
CID102288	419.667	0.039	150.000	0.153
CID104727	275.967	0.248	419.800	0.100
CID104746	299.600	0.175	181.000	0.071
CID104747	382.900	0.208	438.000	0.262
CID104799	302.933	0.186	900.000	<0.001
CID104807	294.708	0.233	346.750	0.012
CID104810	269.351	0.372	268.000	0.180
CID104842	278.028	0.438	501.333	0.212
CID104849	298.571	0.364	150.000	0.288
CID105035	303.802	0.311	189.000	0.067
CID105111	274.283	0.314	226.000	0.073
CID105145	255.278	0.431	700.000	0.011
CID107744	351.947	0.242	900.000	0.031
CID107865	311.625	0.131	164.000	0.011
CID107901	262.545	0.256	217.750	0.041
CID107935	267.276	0.197	420.538	0.087
CID108007	295.565	0.181	549.571	0.041
CID108150	233.792	0.331	533.000	0.080
CID108169	298.794	0.332	274.350	0.106
CID110634	240.625	0.305	153.000	0.193
CID110635	253.542	0.438	817.000	0.016
CID111123	244.219	0.230	207.429	0.031
CID111332	240.167	0.172	188.000	0.054
CID115215	226.750	0.176	181.000	<0.001

CID119031	333.152	0.212	676.000	0.174
CID119182	291.121	0.153	496.000	0.036
CID119196	327.750	0.152	150.000	0.095
CID119373	265.963	0.331	997.000	<0.001
CID119607	287.714	0.197	173.000	0.223
CID121591	285.643	0.238	177.500	0.111
CID122877	221.333	0.176	175.000	0.018
CID123606	238.063	0.173	800.000	0.025
CID123619	282.795	0.179	800.000	0.029
CID123865	272.776	0.146	272.417	0.071
CID124087	315.321	0.128	800.000	0.074
CID124331	470.667	0.006	724.000	<0.001
CID126690	276.000	0.194	181.000	0.019
CID128872	205.000	0.014	572.000	0.006
CID130966	237.714	0.146	377.600	0.031
CID132999	245.222	0.191	250.000	0.087
CID133246	353.095	0.258	161.000	0.163
CID136539	214.535	0.365	168.333	0.063
CID141643	380.098	0.219	232.250	0.176
CID145068	333.582	0.100	511.265	0.043
CID145729	425.953	0.379	923.000	0.005
CID148121	293.320	0.466	166.000	0.066
CID148123	317.241	0.280	504.206	0.223
CID148177	286.189	0.219	463.917	0.104
CID148191	230.818	0.211	212.000	0.047
CID148195	281.785	0.247	639.538	0.097
CID148201	289.391	0.195	968.000	<0.001
CID149096	380.610	0.185	493.500	0.067
CID150311	273.245	0.305	487.500	0.145
CID151193	294.016	0.276	485.000	0.104

CID151199	268.901	0.398	204.857	0.203
CID153751	346.268	0.160	293.000	0.138
CID153997	294.263	0.124	864.000	<0.001
CID156391	295.317	0.262	337.667	0.180
CID156413	331.293	0.155	781.000	0.054
CID156418	268.891	0.331	169.000	0.205
CID158786	272.750	0.100	370.000	<0.001
CID159324	302.207	0.185	331.300	0.077
CID159594	242.500	0.228	167.000	0.065
CID159832	274.809	0.122	311.625	0.116
CID159854	374.462	0.100	826.000	<0.001
CID160355	262.320	0.307	634.923	0.195
CID160913	420.188	0.460	336.000	0.041
CID161113	232.300	0.315	180.333	0.101
CID163659	230.582	0.311	562.000	0.121
CID165904	248.429	0.207	205.000	0.028
CID166617	183.833	0.338	170.500	0.181
CID172197	329.206	0.068	585.500	0.122
CID176166	256.868	0.231	706.556	0.075
CID176873	273.917	0.184	170.500	0.105
CID179337	355.138	0.153	444.000	0.025
CID187790	281.840	0.345	210.200	0.278
CID191247	173.000	0.101	212.000	0.025
CID194173	234.080	0.122	161.000	0.052
CID208898	269.259	0.242	150.000	0.147
CID208908	307.412	0.303	504.750	0.119
CID213040	299.300	0.110	282.000	0.022
CID216237	228.160	0.404	429.000	0.061
CID216326	340.961	0.152	567.000	0.128
CID216453	462.923	0.127	172.333	0.060

CID216468	257.167	0.157	344.333	0.081
CID219023	242.600	0.284	170.000	0.019
CID219100	238.000	0.134	170.000	0.065
CID220401	253.109	0.339	187.000	0.171
CID222786	296.228	0.130	707.000	0.046
CID241902	248.089	0.401	827.000	<0.001
CID252682	352.778	0.114	613.000	0.014
CID259331	281.959	0.360	166.000	0.130
CID275196	334.167	0.183	431.500	0.040
CID301389	279.565	0.296	180.000	0.034
CID312145	310.020	0.305	517.944	0.169
CID324081	260.650	0.255	665.500	0.006
CID327404	381.571	0.329	506.000	0.010
CID344265	278.743	0.265	190.333	0.240
CID358641	355.552	0.243	209.500	0.139
CID358880	242.500	0.056	159.000	0.009
CID371509	180.000	0.328	250.000	0.012
CID387447	293.390	0.223	556.976	0.051
CID392622	295.656	0.345	476.438	0.243
CID394347	950.000	0.012	979.000	<0.001
CID400010	254.316	0.250	583.800	0.110
CID400769	245.250	0.237	748.143	0.102
CID403923	346.324	0.189	604.000	0.012
CID410253	229.529	0.223	201.000	0.012
CID423209	205.000	0.049	719.000	<0.001
CID425430	420.941	0.331	800.000	0.010
CID439285	320.659	0.304	491.800	0.088
CID439501	295.364	0.197	544.111	0.144
CID439530	301.783	0.196	237.231	0.291
CID439655	330.659	0.349	900.000	<0.001

CID439693	291.111	0.234	363.500	0.085
CID441276	285.687	0.309	540.560	0.171
CID442070	289.247	0.256	367.318	0.217
CID442514	287.967	0.261	282.065	0.108
CID442530	347.089	0.249	338.833	0.079
CID442972	318.452	0.249	372.571	0.157
CID442977	233.000	0.110	174.500	0.006
CID443090	298.719	0.192	205.000	0.131
CID443154	293.567	0.242	199.000	0.135
CID443314	293.157	0.122	245.814	0.128
CID444503	180.714	0.270	159.000	0.172
CID444593	237.688	0.380	183.000	0.184
CID444795	314.512	0.200	549.150	<0.001
CID445008	197.714	0.283	184.000	0.033
CID445154	295.613	0.395	534.825	0.059
CID445226	293.783	0.312	165.000	0.124
CID445533	236.357	0.334	163.000	0.100
CID445643	273.168	0.418	661.250	0.113
CID446129	260.500	0.264	300.000	0.020
CID446313	304.889	0.149	336.000	0.025
CID446378	305.279	0.288	643.455	0.064
CID446838	291.657	0.238	276.750	0.140
CID447043	308.638	0.247	170.000	0.259
CID447316	288.952	0.248	528.333	0.070
CID447612	229.700	0.137	160.000	0.069
CID447700	386.769	0.136	486.500	0.064
CID447865	204.500	0.468	163.000	0.021
CID448013	216.870	0.378	161.000	0.022
CID448545	331.760	0.142	334.250	0.033
CID448657	186.938	0.337	196.000	0.115

CID448839	262.297	0.455	182.000	0.230
CID448991	279.696	0.276	843.000	0.032
CID449051	300.625	0.151	173.200	0.207
CID449171	314.605	0.235	654.000	0.071
CID449193	284.741	0.232	810.000	0.055
CID449223	414.667	0.040	205.000	0.059
CID449459	293.626	0.301	537.630	0.084
CID449540	252.708	0.155	161.000	0.130
CID449546	233.053	0.306	340.000	0.022
CID451668	311.253	0.305	467.773	0.097
CID451931	288.375	0.060	233.000	0.015
CID452548	331.041	0.215	463.333	0.052
CID456201	287.824	0.321	537.000	0.145
CID457954	306.421	0.308	683.667	0.067
CID460612	356.776	0.136	265.000	0.138
CID466151	291.500	0.166	548.250	0.089
CID493570	337.044	0.357	231.000	0.232
CID518605	258.373	0.231	811.125	0.029
CID520535	317.240	0.265	388.517	0.198
CID521017	279.156	0.188	387.000	0.048
CID638072	334.842	0.194	207.000	0.232
CID657237	299.183	0.172	529.941	0.107
CID667490	346.162	0.248	478.143	0.155
CID720071	278.155	0.263	194.667	0.159
CID1048845	293.763	0.264	203.000	0.156
CID1474853	211.059	0.310	714.500	0.082
CID1548886	286.584	0.297	440.519	0.222
CID1548999	276.690	0.182	187.000	0.057
CID1893730	301.313	0.261	538.000	0.009
CID2723601	306.632	0.316	321.500	0.129

CID2724126	220.648	0.322	168.500	0.095
CID2724189	221.063	0.334	207.000	0.016
CID2724385	297.159	0.206	498.400	0.104
CID2724387	253.120	0.419	800.000	<0.001
CID2733525	308.399	0.321	579.093	0.066
CID2782689	257.667	0.429	638.000	0.029
CID2795457	210.091	0.254	166.000	0.053
CID2807595	217.833	0.325	330.000	0.020
CID2812173	266.578	0.413	253.333	0.192
CID2987927	247.867	0.231	181.000	0.201
CID3001028	330.225	0.238	368.417	0.181
CID3001055	263.044	0.270	814.000	0.021
CID3005572	290.563	0.288	594.000	0.094
CID3006531	287.603	0.193	471.810	0.194
CID3025986	244.636	0.239	493.000	0.142
CID3031661	265.850	0.209	208.000	0.007
CID3032581	297.288	0.181	243.737	0.286
CID3034010	235.721	0.309	747.000	0.079
CID3037617	282.857	0.170	407.000	0.026
CID3038522	291.742	0.221	317.000	0.123
CID3052775	279.061	0.286	900.000	0.048
CID3062316	311.517	0.296	521.029	0.076
CID3070537	215.364	0.417	243.500	0.090
CID3082777	214.909	0.240	263.000	0.036
CID3084046	279.033	0.130	234.000	0.223
CID3733518	301.800	0.267	773.000	<0.001
CID4998669	190.421	0.322	719.000	0.016
CID5001396	275.994	0.319	500.750	0.172
CID5222465	269.660	0.142	583.636	0.098
CID5280343	316.459	0.072	370.477	<0.001

CID5280360	344.820	0.093	749.150	0.292
CID5280453	296.095	0.371	435.429	0.002
CID5280483	297.688	0.392	528.000	0.086
CID5280961	303.970	0.328	508.264	0.010
CID5281004	324.427	0.243	410.667	0.232
CID5281040	357.857	0.272	374.000	0.202
CID5281051	253.649	0.321	248.000	0.096
CID5281078	297.960	0.359	390.667	0.163
CID5281321	244.235	0.280	612.333	0.043
CID5281614	235.907	0.202	462.200	0.163
CID5281767	281.456	0.333	631.550	0.132
CID5281828	244.200	0.423	171.333	0.096
CID5281877	271.576	0.152	293.000	0.247
CID5281888	307.143	0.160	807.000	0.048
CID5281955	264.110	0.250	529.333	0.010
CID5282375	317.250	0.230	744.500	0.071
CID5282379	269.903	0.308	364.714	0.016
CID5282381	273.056	0.296	707.000	0.050
CID5282412	270.324	0.266	239.000	0.072
CID5282451	255.974	0.171	151.000	0.284
CID5284344	235.738	0.317	800.000	<0.001
CID5284371	335.224	0.178	245.000	0.238
CID5284380	254.348	0.308	159.000	0.046
CID5284513	308.053	0.244	307.143	0.068
CID5284558	220.208	0.143	800.000	0.008
CID5284566	271.982	0.466	159.000	0.164
CID5284616	293.316	0.307	552.956	0.208
CID5287969	243.558	0.300	598.095	0.235
CID5288382	283.500	0.342	556.313	0.150
CID5288628	196.780	0.209	900.000	<0.001

CID5288783	343.176	0.241	800.000	0.040
CID5288826	348.449	0.091	704.154	0.161
CID5289247	239.367	0.312	331.400	0.086
CID5289419	213.882	0.231	820.667	0.044
CID5311051	264.536	0.219	150.000	<0.001
CID5311181	263.790	0.368	678.000	0.184
CID5311263	265.007	0.270	393.133	0.137
CID5311497	281.524	0.231	381.000	0.006
CID5311498	299.507	0.310	171.000	0.169
CID5323510	406.304	0.181	475.000	0.009
CID5327044	259.000	0.203	161.000	0.205
CID5328779	283.261	0.214	550.167	0.209
CID5329006	210.333	0.129	444.500	0.012
CID5329032	284.778	0.076	313.250	0.034
CID5329098	264.357	0.379	450.900	0.239
CID5330175	330.000	0.057	317.500	0.024
CID5352019	301.727	0.275	250.800	0.016
CID5353562	371.085	0.237	386.143	0.103
CID5353586	310.906	0.055	352.778	0.184
CID5353980	298.393	0.232	228.500	0.145
CID5359264	278.792	0.347	237.643	0.238
CID5359282	136.000	0.436	188.000	<0.001
CID5359476	274.347	0.276	472.250	0.194
CID5360515	272.992	0.357	800.000	0.035
CID5360621	289.889	0.276	199.500	0.146
CID5362420	292.846	0.221	327.000	0.026
CID5362440	287.255	0.326	784.000	0.080
CID5362564	220.283	0.440	206.111	0.110
CID5381226	303.328	0.233	389.125	0.188
CID5386092	275.138	0.230	167.000	0.158

CID5458428	284.571	0.311	559.429	0.067
CID5460555	235.169	0.327	176.000	0.093
CID5462328	293.743	0.242	365.000	0.052
CID5469318	239.778	0.133	441.111	0.044
CID5472495	290.684	0.170	627.636	0.127
CID5474206	253.826	0.351	514.000	<0.001
CID5476374	245.237	0.343	249.667	0.175
CID5477614	271.259	0.163	199.000	0.010
CID5479530	394.889	0.217	199.000	0.198
CID5480431	245.330	0.276	812.000	0.015
CID5481350	317.400	0.231	700.000	<0.001
CID5484731	286.000	0.252	161.000	0.149
CID5487525	278.938	0.096	344.750	0.022
CID5494407	215.875	0.385	214.000	0.064
CID5494424	216.850	0.229	180.000	0.115
CID5702553	278.748	0.292	211.619	0.174
CID6091659	275.095	0.072	332.833	0.006
CID6167828	301.064	0.283	211.500	0.169
CID6323490	302.832	0.270	222.167	0.212
CID6324671	319.435	0.224	259.667	0.235
CID6419957	303.813	0.117	249.880	0.127
CID6420135	199.429	0.253	157.000	0.045
CID6432013	228.671	0.395	163.000	0.119
CID6433557	296.887	0.344	392.500	0.265
CID6435110	285.315	0.431	368.750	0.085
CID6438891	190.000	0.396	163.000	0.028
CID6439072	235.714	0.251	399.000	<0.001
CID6440175	266.241	0.256	491.600	0.132
CID6442177	295.770	0.325	290.063	0.177
CID6444692	343.333	0.071	817.500	0.006

CID6445533	325.163	0.223	500.667	0.194
CID6445540	248.571	0.324	205.667	0.016
CID6445562	322.081	0.153	605.833	0.112
CID6450551	292.765	0.266	352.286	0.197
CID6450813	226.474	0.347	564.000	0.014
CID6456015	254.679	0.245	323.500	0.110
CID6505803	292.837	0.275	576.889	0.098
CID6540268	230.660	0.274	224.333	0.216
CID6540295	234.803	0.384	190.250	0.046
CID6603857	239.833	0.204	616.667	0.018
CID6604200	329.448	0.255	454.000	0.034
CID6713928	213.184	0.234	233.667	0.040
CID6850715	281.100	0.284	220.739	0.221
CID6850726	234.071	0.172	271.167	0.014
CID6850753	247.951	0.164	227.333	0.291
CID6914628	188.667	0.200	521.000	0.042
CID6917781	209.200	0.357	800.000	0.019
CID6918220	248.080	0.294	181.000	0.029
CID6918250	250.667	0.227	170.000	0.074
CID6918289	272.739	0.333	325.154	0.097
CID6918296	259.465	0.210	150.000	0.110
CID6918365	246.510	0.442	162.000	0.162
CID6918403	305.217	0.127	677.000	0.034
CID6918412	260.583	0.230	425.000	0.017
CID6918453	271.946	0.277	499.500	0.032
CID6918454	284.618	0.230	313.643	0.120
CID6918456	277.938	0.306	380.500	0.080
CID6918508	229.333	0.174	725.500	0.005
CID6918537	262.895	0.272	196.000	0.081
CID6918558	228.091	0.432	800.000	0.017

CID6918638	349.538	0.158	411.800	0.131
CID6918837	330.721	0.198	606.000	0.162
CID9547169	216.412	0.135	202.500	0.039
CID9547917	213.647	0.365	159.000	0.064
CID9549284	283.872	0.256	300.600	0.103
CID9549299	283.364	0.320	338.143	0.085
CID9562060	280.510	0.234	330.143	0.164
CID9568512	258.474	0.383	205.333	0.095
CID9796068	261.750	0.185	180.000	0.051
CID9800306	244.700	0.205	800.000	<0.001
CID9804302	351.800	0.157	425.000	0.067
CID9809714	247.000	0.311	778.000	0.182
CID9825149	273.368	0.195	700.000	0.016
CID9826528	307.400	0.228	443.583	0.050
CID9832447	254.500	0.155	575.000	0.031
CID9843206	269.286	0.256	436.000	0.049
CID9849735	372.050	0.136	386.100	0.047
CID9854073	326.000	0.210	823.000	<0.001
CID9863342	354.300	0.141	432.625	0.043
CID9865515	315.500	0.142	530.500	0.082
CID9868037	222.556	0.179	700.000	0.015
CID9868524	233.353	0.185	372.500	0.086
CID9874912	214.143	0.236	753.667	0.013
CID9881652	262.857	0.090	595.250	0.048
CID9913881	305.600	0.097	734.000	0.016
CID9914412	229.900	0.297	500.500	0.101
CID9915743	292.485	0.233	478.583	0.172
CID9926791	228.111	0.343	820.000	0.028
CID9933475	287.426	0.289	575.400	0.195
CID9977819	277.640	0.177	709.000	0.226

CID10027278	229.731	0.234	665.571	0.061
CID10096043	265.810	0.318	221.000	0.062
CID10113978	304.526	0.274	546.545	0.190
CID10126189	201.067	0.421	504.000	0.014
CID10127622	310.550	0.265	396.714	0.037
CID10138259	203.000	0.336	573.333	0.232
CID10172943	237.600	0.222	687.500	0.047
CID10218498	261.722	0.272	186.500	0.152
CID10302451	243.000	0.234	249.000	0.114
CID10317566	210.000	0.232	237.000	<0.001
CID10319891	232.500	0.095	334.000	0.009
CID10322450	313.895	0.108	326.000	0.049
CID10384072	221.750	0.237	658.000	0.112
CID10437018	250.700	0.365	895.500	0.006
CID10458325	277.238	0.180	190.000	0.011
CID10460379	310.722	0.256	424.000	0.171
CID10461815	301.609	0.152	496.000	0.143
CID10953556	155.286	0.203	211.000	0.022
CID11154925	224.533	0.261	466.000	0.023
CID11167602	277.786	0.219	425.000	0.064
CID11196273	262.221	0.351	240.900	0.158
CID11210478	412.200	0.061	611.600	0.059
CID11228183	254.059	0.307	439.125	0.118
CID11234052	311.167	0.196	417.000	0.103
CID11282283	254.889	0.174	461.000	0.011
CID11349170	262.571	0.168	823.000	<0.001
CID11364421	253.043	0.290	369.667	0.099
CID11404337	252.290	0.203	643.000	0.009
CID11414799	235.667	0.160	156.000	<0.001
CID11485656	251.391	0.291	487.000	0.224

CID11494412	324.556	0.149	413.333	0.011
CID11511120	302.176	0.131	576.000	0.025
CID11520894	240.188	0.270	170.500	0.026
CID11534420	256.063	0.288	327.000	0.086
CID11578515	296.871	0.193	849.600	0.008
CID11599950	247.875	0.105	159.000	<0.001
CID11646823	249.313	0.244	166.000	0.137
CID11647372	311.556	0.120	690.000	0.049
CID11655119	216.250	0.170	314.000	0.022
CID11667893	285.290	0.213	200.333	0.151
CID11671467	215.353	0.345	482.333	0.119
CID11679764	355.429	0.331	170.000	0.223
CID11707110	321.200	0.157	414.111	0.042
CID11713159	218.333	0.273	594.333	0.253
CID11714998	203.900	0.364	172.000	0.102
CID11717001	252.923	0.203	735.000	0.011
CID11822705	242.417	0.251	161.000	0.067
CID11953947	221.000	0.324	192.000	<0.001
CID11966249	263.750	0.110	177.000	0.266
CID11966311	320.036	<0.001	265.311	0.012
CID11970251	289.203	0.353	309.250	0.043
CID11977753	288.714	0.374	448.625	0.181
CID11987672	252.755	0.361	232.250	0.244
CID13783824	201.600	0.290	159.000	0.094
CID13922196	234.667	0.312	181.000	0.068
CID14476155	235.889	0.379	157.000	0.123
CID14942883	269.000	0.143	258.000	0.019
CID15950351	297.667	0.042	613.000	<0.001
CID16129975	224.773	0.211	342.429	0.025
CID16130049	206.333	0.229	853.000	<0.001

CID16134956	290.895	0.217	199.000	0.241
CID16217590	263.250	0.213	192.000	0.270
CID16725726	229.462	0.267	756.000	0.103
CID16736529	252.286	0.299	160.500	0.042
CID16759369	498.667	0.026	441.000	0.018
CID16760281	228.157	0.344	266.500	0.150
CID16760691	270.000	0.052	814.000	<0.001
CID17754438	248.000	0.144	915.000	<0.001
CID17755052	363.125	0.101	598.583	0.061
CID18991124	201.045	0.439	168.000	0.100
CID20042692	900.000	0.008	808.739	<0.001
CID20620240	286.917	0.119	602.500	0.041
CID22024915	176.000	0.118	455.750	0.012
CID23624255	211.077	0.258	199.000	0.018
CID23674191	252.558	0.308	179.500	0.227
CID23690938	276.195	0.397	180.200	0.263
CID23724531	239.900	0.213	172.000	0.039
CID23725625	293.548	0.262	198.000	0.175
CID24180719	283.133	0.171	365.375	0.094
CID24756910	250.667	0.155	892.000	0.009
CID24764437	227.167	0.350	180.000	0.030
CID24764449	226.200	0.134	533.500	0.040
CID24771867	223.500	0.315	581.167	0.123
CID24776445	238.474	0.268	486.000	0.041
CID24779724	291.917	0.210	982.000	<0.001
CID24783227	267.621	0.184	253.000	0.023
CID24785538	234.222	0.210	556.667	0.037
CID24794418	341.167	0.061	822.000	0.011
CID24838940	251.357	0.184	170.000	0.207
CID24840378	330.697	0.167	916.000	<0.001

CID24892733	272.600	0.197	161.000	0.093
CID24901704	303.000	0.144	800.000	<0.001
CID24901722	307.933	0.155	260.000	0.041
CID24941245	256.640	0.184	182.000	0.045
CID24956525	274.593	0.274	345.667	0.020
CID24964624	264.560	0.311	589.800	0.053
CID24978514	308.379	0.112	241.175	0.162
CID24978538	249.742	0.271	750.000	0.022
CID25027363	321.333	0.122	246.500	0.080
CID25033539	503.500	0.058	835.000	<0.001
CID25088416	223.688	0.217	163.000	0.026
CID25102847	313.846	0.238	424.667	0.005
CID25126797	255.273	0.227	162.000	0.122
CID25167777	352.250	0.208	540.000	0.004
CID25171647	481.750	0.058	164.000	<0.001
CID25182616	233.125	0.193	417.778	0.171
CID26275995	282.581	0.392	461.800	0.161
CID27885548	312.346	0.200	445.519	0.135
CID28777137	267.619	0.164	318.000	0.141
CID42611257	340.147	0.227	339.611	0.099
CID42642645	323.783	0.104	698.222	0.291
CID44134894	305.049	0.148	328.208	0.067
CID44137945	274.133	0.150	823.000	0.010
CID44182395	409.333	0.074	209.000	0.030
CID44187953	273.167	0.164	754.500	0.015
CID44228987	430.429	0.093	234.333	0.054
CID44263835	305.342	0.150	247.887	0.097
CID44268108	300.456	0.157	457.118	0.136
CID44284481	300.876	0.213	296.353	0.261
CID44472890	206.745	0.318	154.000	0.249

CID44483210	217.055	0.234	170.200	0.094
CID44593851	224.590	0.257	270.000	0.016
CID44604932	194.471	0.344	185.000	0.070
CID44607530	401.067	0.090	690.333	0.019
CID45100498	265.600	0.172	184.000	0.096
CID45142457	230.000	0.036	206.000	<0.001
CID45270897	282.280	0.213	507.000	0.055
CID45382213	198.000	0.233	159.000	0.023
CID45480163	227.500	0.328	234.111	0.122
CID46216795	238.200	0.278	262.000	0.034
CID46885626	541.000	0.022	704.500	0.045
CID46911863	305.667	0.203	182.000	0.027
CID49769060	252.600	0.301	205.500	0.182
CID49769423	169.000	0.131	190.000	0.029
CID49837887	213.000	0.127	900.000	<0.001
CID49867926	257.600	0.368	207.000	0.008
CID51346199	217.000	0.264	220.000	0.195
CID53346510	218.391	0.462	241.333	0.193
CID53352191	189.867	0.426	193.667	0.123
CID53384665	222.974	0.269	152.000	0.134
CID53427792	261.474	0.443	199.250	0.157
CID53477714	306.088	0.165	473.586	0.161
CID53629505	317.439	0.212	461.000	0.223
CID53630776	200.500	0.203	180.000	<0.001
CID53630877	251.029	0.265	215.000	0.032
CID54609526	282.538	0.184	154.000	0.175
CID54684141	326.214	0.202	523.600	0.072
CID54710406	252.583	0.084	700.000	<0.001
CID54734719	269.979	0.312	235.833	0.058
CID56603655	255.479	0.367	397.000	<0.001

CID56603668	265.857	0.083	329.250	0.006
CID56649450	484.400	0.120	199.000	<0.001
CID56684138	248.069	0.117	159.000	0.185
CID56841764	320.250	0.122	162.000	0.008
CID56841800	219.692	0.276	167.000	0.023
CID56841999	311.154	0.092	288.000	<0.001
CID56842042	333.292	0.168	262.000	0.008
CID56842082	331.000	0.088	186.000	0.011
CID56842117	327.128	0.275	271.500	0.045
CID56842118	190.286	0.430	182.000	0.004
CID56842121	328.571	0.277	310.000	0.054
CID56842157	418.652	0.105	205.000	0.022
CID56843240	246.625	0.220	171.000	0.041
CID56952026	238.667	0.102	183.000	<0.001
CID57390074	286.333	0.085	237.500	0.005
CID66553073	242.200	0.159	154.000	0.037
CID70679302	188.941	0.371	161.000	0.120
CID70680240	217.795	0.337	243.000	0.147
CID70683023	228.273	0.374	182.000	0.147
CID71297189	193.667	0.430	252.000	<0.001
CID71308162	288.182	0.091	447.000	0.052
CID71463576	188.815	0.327	218.750	0.034
CID71464519	225.941	0.211	218.500	0.251
