# Supplementary material for: A computational method for the identification of candidate drugs for non-small cell lung cancer
Source: PLoS One. 2017 Aug 18;12(8):e0183411. doi: 10.1371/journal.pone.0183411 (PMC5562320; doi:10.1371/journal.pone.0183411)
Supplement: S5 Table — (DOCX) [file pone.0183411.s005.docx]

**S5 Table.** Clustering results by the EM algorithm on 1007 candidate compounds and ten approved NSCLC drugs

| **PubChem ID** | **Category** |
| --- | --- |
| CID60843^#^ | cluster3 |
| CID57166^#^ | cluster3 |
| CID4033^#^ | cluster3 |
| CID38904^#^ | cluster3 |
| CID36314^#^ | cluster3 |
| CID176870^#^ | cluster3 |
| CID126941^#^ | cluster3 |
| CID123631^#^ | cluster3 |
| CID11626560^#^ | cluster3 |
| CID10184653^#^ | cluster3 |
| CID6 | cluster2 |
| CID15 | cluster3 |
| CID137 | cluster0 |
| CID138 | cluster0 |
| CID143 | cluster2 |
| CID174 | cluster0 |
| CID180 | cluster0 |
| CID190 | cluster2 |
| CID196 | cluster0 |
| CID206 | cluster0 |
| CID237 | cluster2 |
| CID241 | cluster2 |
| CID271 | cluster3 |
| CID311 | cluster1 |
| CID338 | cluster2 |
| CID402 | cluster2 |
| CID444 | cluster2 |
| CID597 | cluster0 |
| CID602 | cluster2 |
| CID611 | cluster2 |
| CID612 | cluster2 |
| CID702 | cluster3 |
| CID712 | cluster2 |
| CID743 | cluster0 |
| CID764 | cluster2 |
| CID778 | cluster2 |
| CID787 | cluster0 |
| CID790 | cluster2 |
| CID836 | cluster0 |
| CID857 | cluster0 |
| CID876 | cluster0 |
| CID887 | cluster0 |
| CID892 | cluster2 |
| CID996 | cluster2 |
| CID1032 | cluster3 |
| CID1048 | cluster2 |
| CID1049 | cluster2 |
| CID1060 | cluster3 |
| CID1099 | cluster0 |
| CID1112 | cluster3 |
| CID1130 | cluster2 |
| CID1135 | cluster0 |
| CID1140 | cluster2 |
| CID1153 | cluster3 |
| CID1175 | cluster2 |
| CID1188 | cluster2 |
| CID1401 | cluster3 |
| CID1464 | cluster0 |
| CID1493 | cluster0 |
| CID1674 | cluster2 |
| CID1697 | cluster1 |
| CID1775 | cluster2 |
| CID1923 | cluster2 |
| CID1983 | cluster2 |
| CID2007 | cluster0 |
| CID2016 | cluster0 |
| CID2022 | cluster0 |
| CID2044 | cluster2 |
| CID2051 | cluster1 |
| CID2088 | cluster2 |
| CID2092 | cluster0 |
| CID2094 | cluster2 |
| CID2141 | cluster2 |
| CID2148 | cluster2 |
| CID2156 | cluster2 |
| CID2160 | cluster2 |
| CID2179 | cluster2 |
| CID2187 | cluster0 |
| CID2202 | cluster2 |
| CID2244 | cluster2 |
| CID2265 | cluster2 |
| CID2336 | cluster2 |
| CID2349 | cluster0 |
| CID2375 | cluster2 |
| CID2380 | cluster2 |
| CID2459 | cluster0 |
| CID2475 | cluster0 |
| CID2478 | cluster2 |
| CID2519 | cluster2 |
| CID2554 | cluster2 |
| CID2577 | cluster1 |
| CID2578 | cluster2 |
| CID2662 | cluster2 |
| CID2708 | cluster2 |
| CID2719 | cluster2 |
| CID2733 | cluster2 |
| CID2746 | cluster0 |
| CID2756 | cluster2 |
| CID2764 | cluster2 |
| CID2767 | cluster2 |
| CID2770 | cluster2 |
| CID2776 | cluster3 |
| CID2794 | cluster3 |
| CID2907 | cluster2 |
| CID2955 | cluster2 |
| CID3016 | cluster2 |
| CID3032 | cluster2 |
| CID3071 | cluster2 |
| CID3082 | cluster0 |
| CID3108 | cluster2 |
| CID3121 | cluster3 |
| CID3152 | cluster2 |
| CID3213 | cluster2 |
| CID3283 | cluster0 |
| CID3300 | cluster0 |
| CID3305 | cluster0 |
| CID3307 | cluster0 |
| CID3308 | cluster2 |
| CID3347 | cluster2 |
| CID3348 | cluster2 |
| CID3365 | cluster2 |
| CID3366 | cluster2 |
| CID3385 | cluster2 |
| CID3394 | cluster2 |
| CID3397 | cluster2 |
| CID3414 | cluster0 |
| CID3440 | cluster2 |
| CID3446 | cluster2 |
| CID3454 | cluster2 |
| CID3467 | cluster2 |
| CID3468 | cluster0 |
| CID3503 | cluster2 |
| CID3589 | cluster2 |
| CID3624 | cluster0 |
| CID3652 | cluster2 |
| CID3657 | cluster2 |
| CID3672 | cluster2 |
| CID3676 | cluster2 |
| CID3690 | cluster2 |
| CID3715 | cluster2 |
| CID3767 | cluster2 |
| CID3883 | cluster2 |
| CID3899 | cluster2 |
| CID3902 | cluster2 |
| CID3922 | cluster3 |
| CID3950 | cluster2 |
| CID3954 | cluster3 |
| CID3958 | cluster0 |
| CID4075 | cluster2 |
| CID4095 | cluster2 |
| CID4156 | cluster2 |
| CID4169 | cluster2 |
| CID4173 | cluster0 |
| CID4189 | cluster3 |
| CID4211 | cluster0 |
| CID4212 | cluster2 |
| CID4261 | cluster2 |
| CID4485 | cluster2 |
| CID4495 | cluster2 |
| CID4553 | cluster2 |
| CID4578 | cluster0 |
| CID4594 | cluster2 |
| CID4595 | cluster2 |
| CID4604 | cluster0 |
| CID4609 | cluster2 |
| CID4673 | cluster2 |
| CID4679 | cluster3 |
| CID4705 | cluster2 |
| CID4707 | cluster3 |
| CID4708 | cluster3 |
| CID4740 | cluster2 |
| CID4763 | cluster1 |
| CID4766 | cluster0 |
| CID4784 | cluster0 |
| CID4873 | cluster0 |
| CID4909 | cluster0 |
| CID4911 | cluster2 |
| CID4913 | cluster0 |
| CID4915 | cluster2 |
| CID4939 | cluster0 |
| CID4946 | cluster2 |
| CID4971 | cluster3 |
| CID4973 | cluster3 |
| CID4993 | cluster2 |
| CID4996 | cluster0 |
| CID5035 | cluster2 |
| CID5070 | cluster2 |
| CID5090 | cluster2 |
| CID5213 | cluster2 |
| CID5215 | cluster2 |
| CID5234 | cluster0 |
| CID5235 | cluster2 |
| CID5245 | cluster2 |
| CID5311 | cluster2 |
| CID5333 | cluster2 |
| CID5342 | cluster2 |
| CID5344 | cluster0 |
| CID5345 | cluster1 |
| CID5381 | cluster3 |
| CID5394 | cluster2 |
| CID5426 | cluster2 |
| CID5453 | cluster2 |
| CID5472 | cluster2 |
| CID5546 | cluster0 |
| CID5578 | cluster2 |
| CID5590 | cluster0 |
| CID5641 | cluster2 |
| CID5744 | cluster0 |
| CID5746 | cluster2 |
| CID5755 | cluster2 |
| CID5757 | cluster2 |
| CID5760 | cluster0 |
| CID5768 | cluster2 |
| CID5770 | cluster2 |
| CID5790 | cluster0 |
| CID5793 | cluster2 |
| CID5798 | cluster1 |
| CID5819 | cluster2 |
| CID5825 | cluster0 |
| CID5833 | cluster2 |
| CID5834 | cluster3 |
| CID5865 | cluster2 |
| CID5897 | cluster2 |
| CID5904 | cluster2 |
| CID5905 | cluster0 |
| CID5920 | cluster2 |
| CID5954 | cluster2 |
| CID5955 | cluster2 |
| CID5959 | cluster0 |
| CID5978 | cluster2 |
| CID5984 | cluster0 |
| CID5994 | cluster2 |
| CID6006 | cluster0 |
| CID6013 | cluster2 |
| CID6035 | cluster0 |
| CID6047 | cluster2 |
| CID6049 | cluster0 |
| CID6058 | cluster2 |
| CID6076 | cluster2 |
| CID6104 | cluster0 |
| CID6115 | cluster2 |
| CID6163 | cluster0 |
| CID6197 | cluster2 |
| CID6199 | cluster0 |
| CID6212 | cluster0 |
| CID6249 | cluster0 |
| CID6251 | cluster2 |
| CID6252 | cluster2 |
| CID6256 | cluster3 |
| CID6279 | cluster2 |
| CID6325 | cluster0 |
| CID6338 | cluster2 |
| CID6421 | cluster0 |
| CID6436 | cluster2 |
| CID6503 | cluster0 |
| CID6508 | cluster0 |
| CID6579 | cluster0 |
| CID6675 | cluster0 |
| CID6741 | cluster2 |
| CID6742 | cluster3 |
| CID6802 | cluster2 |
| CID7099 | cluster0 |
| CID7187 | cluster3 |
| CID7847 | cluster2 |
| CID7950 | cluster0 |
| CID8378 | cluster2 |
| CID8549 | cluster2 |
| CID8778 | cluster0 |
| CID8977 | cluster3 |
| CID8987 | cluster2 |
| CID9033 | cluster0 |
| CID9048 | cluster0 |
| CID9062 | cluster2 |
| CID9215 | cluster0 |
| CID9250 | cluster2 |
| CID9260 | cluster0 |
| CID9444 | cluster2 |
| CID9679 | cluster0 |
| CID9782 | cluster2 |
| CID9864 | cluster2 |
| CID10340 | cluster0 |
| CID10413 | cluster0 |
| CID10430 | cluster0 |
| CID10457 | cluster0 |
| CID10465 | cluster3 |
| CID10635 | cluster3 |
| CID10786 | cluster0 |
| CID10964 | cluster0 |
| CID11103 | cluster3 |
| CID11178 | cluster0 |
| CID11254 | cluster2 |
| CID11266 | cluster0 |
| CID12137 | cluster0 |
| CID12560 | cluster2 |
| CID12660 | cluster0 |
| CID12699 | cluster2 |
| CID12733 | cluster3 |
| CID12736 | cluster0 |
| CID12967 | cluster2 |
| CID13116 | cluster0 |
| CID13342 | cluster2 |
| CID13588 | cluster0 |
| CID13698 | cluster0 |
| CID13711 | cluster0 |
| CID13945 | cluster3 |
| CID14227 | cluster0 |
| CID14457 | cluster0 |
| CID14797 | cluster0 |
| CID14888 | cluster2 |
| CID14932 | cluster0 |
| CID14941 | cluster0 |
| CID15032 | cluster2 |
| CID16834 | cluster2 |
| CID16850 | cluster0 |
| CID17513 | cluster0 |
| CID18343 | cluster3 |
| CID18407 | cluster3 |
| CID19001 | cluster0 |
| CID19261 | cluster0 |
| CID20279 | cluster2 |
| CID20469 | cluster3 |
| CID21157 | cluster2 |
| CID21672 | cluster0 |
| CID21704 | cluster0 |
| CID22318 | cluster2 |
| CID22469 | cluster2 |
| CID22986 | cluster3 |
| CID23267 | cluster0 |
| CID23424 | cluster0 |
| CID23830 | cluster2 |
| CID23939 | cluster2 |
| CID23963 | cluster2 |
| CID23968 | cluster0 |
| CID23991 | cluster1 |
| CID24083 | cluster0 |
| CID24261 | cluster2 |
| CID24360 | cluster2 |
| CID24441 | cluster0 |
| CID24450 | cluster0 |
| CID24529 | cluster2 |
| CID24538 | cluster0 |
| CID24584 | cluster0 |
| CID24632 | cluster0 |
| CID24748 | cluster0 |
| CID24759 | cluster2 |
| CID24763 | cluster2 |
| CID24769 | cluster0 |
| CID24775 | cluster0 |
| CID24854 | cluster0 |
| CID25419 | cluster0 |
| CID25473 | cluster2 |
| CID26105 | cluster0 |
| CID26879 | cluster0 |
| CID27287 | cluster0 |
| CID27337 | cluster0 |
| CID28486 | cluster2 |
| CID29029 | cluster2 |
| CID29327 | cluster2 |
| CID30323 | cluster2 |
| CID30623 | cluster2 |
| CID30751 | cluster3 |
| CID31083 | cluster0 |
| CID31254 | cluster3 |
| CID31307 | cluster2 |
| CID31401 | cluster2 |
| CID31402 | cluster3 |
| CID31703 | cluster2 |
| CID32874 | cluster0 |
| CID33113 | cluster3 |
| CID33557 | cluster0 |
| CID33576 | cluster0 |
| CID33613 | cluster2 |
| CID33641 | cluster3 |
| CID33776 | cluster2 |
| CID34192 | cluster0 |
| CID34457 | cluster0 |
| CID35370 | cluster2 |
| CID36294 | cluster0 |
| CID36462 | cluster2 |
| CID36797 | cluster0 |
| CID37542 | cluster2 |
| CID37768 | cluster0 |
| CID38003 | cluster0 |
| CID38347 | cluster2 |
| CID38777 | cluster1 |
| CID38852 | cluster2 |
| CID39186 | cluster2 |
| CID39214 | cluster2 |
| CID39562 | cluster2 |
| CID39981 | cluster3 |
| CID40772 | cluster0 |
| CID40839 | cluster0 |
| CID41867 | cluster2 |
| CID42616 | cluster2 |
| CID42890 | cluster2 |
| CID43805 | cluster3 |
| CID44093 | cluster2 |
| CID47326 | cluster0 |
| CID47938 | cluster0 |
| CID49561 | cluster0 |
| CID50515 | cluster1 |
| CID50599 | cluster0 |
| CID50981 | cluster0 |
| CID53232 | cluster2 |
| CID54454 | cluster2 |
| CID54687 | cluster2 |
| CID54886 | cluster0 |
| CID55245 | cluster2 |
| CID55283 | cluster2 |
| CID55466 | cluster0 |
| CID57469 | cluster3 |
| CID60198 | cluster0 |
| CID60606 | cluster2 |
| CID60613 | cluster0 |
| CID60699 | cluster2 |
| CID60749 | cluster2 |
| CID60779 | cluster2 |
| CID60795 | cluster2 |
| CID60808 | cluster2 |
| CID60825 | cluster2 |
| CID60837 | cluster2 |
| CID60852 | cluster0 |
| CID60934 | cluster2 |
| CID60953 | cluster2 |
| CID60955 | cluster0 |
| CID61565 | cluster0 |
| CID61635 | cluster3 |
| CID61671 | cluster0 |
| CID62210 | cluster0 |
| CID64142 | cluster2 |
| CID64730 | cluster0 |
| CID64968 | cluster3 |
| CID65005 | cluster0 |
| CID65041 | cluster0 |
| CID65063 | cluster3 |
| CID65064 | cluster2 |
| CID65091 | cluster3 |
| CID65106 | cluster2 |
| CID65110 | cluster3 |
| CID65191 | cluster0 |
| CID65217 | cluster2 |
| CID65237 | cluster0 |
| CID65253 | cluster0 |
| CID65275 | cluster0 |
| CID65407 | cluster0 |
| CID65628 | cluster2 |
| CID65948 | cluster2 |
| CID65958 | cluster2 |
| CID66868 | cluster2 |
| CID67431 | cluster0 |
| CID67491 | cluster0 |
| CID68152 | cluster0 |
| CID68329 | cluster0 |
| CID68740 | cluster2 |
| CID68770 | cluster3 |
| CID69435 | cluster1 |
| CID71068 | cluster0 |
| CID71184 | cluster0 |
| CID71398 | cluster0 |
| CID71563 | cluster1 |
| CID71616 | cluster0 |
| CID71741 | cluster0 |
| CID72271 | cluster0 |
| CID72402 | cluster0 |
| CID72435 | cluster3 |
| CID72571 | cluster0 |
| CID73009 | cluster0 |
| CID73212 | cluster2 |
| CID74989 | cluster0 |
| CID75142 | cluster0 |
| CID80170 | cluster0 |
| CID82146 | cluster2 |
| CID83970 | cluster0 |
| CID84029 | cluster2 |
| CID84691 | cluster2 |
| CID84759 | cluster0 |
| CID86222 | cluster0 |
| CID89105 | cluster3 |
| CID89594 | cluster2 |
| CID91482 | cluster0 |
| CID92242 | cluster0 |
| CID92727 | cluster2 |
| CID92787 | cluster0 |
| CID93004 | cluster3 |
| CID93577 | cluster0 |
| CID93860 | cluster0 |
| CID94312 | cluster0 |
| CID100153 | cluster0 |
| CID100154 | cluster0 |
| CID100427 | cluster0 |
| CID102288 | cluster3 |
| CID104727 | cluster2 |
| CID104746 | cluster0 |
| CID104747 | cluster2 |
| CID104799 | cluster2 |
| CID104807 | cluster2 |
| CID104810 | cluster0 |
| CID104842 | cluster2 |
| CID104849 | cluster2 |
| CID105035 | cluster2 |
| CID105111 | cluster0 |
| CID105145 | cluster2 |
| CID107744 | cluster3 |
| CID107865 | cluster0 |
| CID107901 | cluster0 |
| CID107935 | cluster2 |
| CID108007 | cluster3 |
| CID108150 | cluster2 |
| CID108169 | cluster2 |
| CID110634 | cluster0 |
| CID110635 | cluster2 |
| CID111123 | cluster0 |
| CID111332 | cluster0 |
| CID115215 | cluster0 |
| CID119031 | cluster3 |
| CID119182 | cluster2 |
| CID119196 | cluster3 |
| CID119373 | cluster3 |
| CID119607 | cluster2 |
| CID121591 | cluster1 |
| CID122877 | cluster0 |
| CID123606 | cluster3 |
| CID123619 | cluster2 |
| CID123865 | cluster2 |
| CID124087 | cluster2 |
| CID124331 | cluster3 |
| CID126690 | cluster0 |
| CID128872 | cluster1 |
| CID130966 | cluster0 |
| CID132999 | cluster0 |
| CID133246 | cluster0 |
| CID136539 | cluster0 |
| CID141643 | cluster0 |
| CID145068 | cluster2 |
| CID145729 | cluster3 |
| CID148121 | cluster0 |
| CID148123 | cluster2 |
| CID148177 | cluster2 |
| CID148191 | cluster0 |
| CID148195 | cluster2 |
| CID148201 | cluster3 |
| CID149096 | cluster2 |
| CID150311 | cluster2 |
| CID151193 | cluster2 |
| CID151199 | cluster0 |
| CID153751 | cluster2 |
| CID153997 | cluster1 |
| CID156391 | cluster2 |
| CID156413 | cluster3 |
| CID156418 | cluster0 |
| CID158786 | cluster2 |
| CID159324 | cluster2 |
| CID159594 | cluster0 |
| CID159832 | cluster2 |
| CID159854 | cluster3 |
| CID160355 | cluster2 |
| CID160913 | cluster3 |
| CID161113 | cluster0 |
| CID163659 | cluster2 |
| CID165904 | cluster0 |
| CID166617 | cluster0 |
| CID172197 | cluster2 |
| CID176166 | cluster2 |
| CID176873 | cluster0 |
| CID179337 | cluster2 |
| CID187790 | cluster0 |
| CID191247 | cluster0 |
| CID194173 | cluster0 |
| CID208898 | cluster2 |
| CID208908 | cluster3 |
| CID213040 | cluster0 |
| CID216237 | cluster1 |
| CID216326 | cluster2 |
| CID216453 | cluster3 |
| CID216468 | cluster2 |
| CID219023 | cluster0 |
| CID219100 | cluster0 |
| CID220401 | cluster0 |
| CID222786 | cluster2 |
| CID241902 | cluster2 |
| CID252682 | cluster2 |
| CID259331 | cluster0 |
| CID275196 | cluster3 |
| CID301389 | cluster0 |
| CID312145 | cluster2 |
| CID324081 | cluster1 |
| CID327404 | cluster1 |
| CID344265 | cluster0 |
| CID358641 | cluster0 |
| CID358880 | cluster0 |
| CID371509 | cluster1 |
| CID387447 | cluster2 |
| CID392622 | cluster2 |
| CID394347 | cluster3 |
| CID400010 | cluster2 |
| CID400769 | cluster2 |
| CID403923 | cluster2 |
| CID410253 | cluster0 |
| CID423209 | cluster2 |
| CID425430 | cluster1 |
| CID439285 | cluster2 |
| CID439501 | cluster2 |
| CID439530 | cluster0 |
| CID439655 | cluster1 |
| CID439693 | cluster2 |
| CID441276 | cluster2 |
| CID442070 | cluster2 |
| CID442514 | cluster0 |
| CID442530 | cluster2 |
| CID442972 | cluster2 |
| CID442977 | cluster0 |
| CID443090 | cluster0 |
| CID443154 | cluster0 |
| CID443314 | cluster0 |
| CID444503 | cluster0 |
| CID444593 | cluster0 |
| CID444795 | cluster2 |
| CID445008 | cluster0 |
| CID445154 | cluster2 |
| CID445226 | cluster0 |
| CID445533 | cluster0 |
| CID445643 | cluster2 |
| CID446129 | cluster0 |
| CID446313 | cluster2 |
| CID446378 | cluster3 |
| CID446838 | cluster0 |
| CID447043 | cluster0 |
| CID447316 | cluster2 |
| CID447612 | cluster0 |
| CID447700 | cluster1 |
| CID447865 | cluster0 |
| CID448013 | cluster0 |
| CID448545 | cluster0 |
| CID448657 | cluster0 |
| CID448839 | cluster0 |
| CID448991 | cluster1 |
| CID449051 | cluster0 |
| CID449171 | cluster2 |
| CID449193 | cluster2 |
| CID449223 | cluster1 |
| CID449459 | cluster2 |
| CID449540 | cluster0 |
| CID449546 | cluster0 |
| CID451668 | cluster2 |
| CID451931 | cluster0 |
| CID452548 | cluster2 |
| CID456201 | cluster2 |
| CID457954 | cluster2 |
| CID460612 | cluster2 |
| CID466151 | cluster1 |
| CID493570 | cluster0 |
| CID518605 | cluster2 |
| CID520535 | cluster2 |
| CID521017 | cluster2 |
| CID638072 | cluster0 |
| CID657237 | cluster2 |
| CID667490 | cluster2 |
| CID720071 | cluster0 |
| CID1048845 | cluster2 |
| CID1474853 | cluster1 |
| CID1548886 | cluster2 |
| CID1548999 | cluster0 |
| CID1893730 | cluster2 |
| CID2723601 | cluster2 |
| CID2724126 | cluster0 |
| CID2724189 | cluster0 |
| CID2724385 | cluster2 |
| CID2724387 | cluster2 |
| CID2733525 | cluster2 |
| CID2782689 | cluster1 |
| CID2795457 | cluster0 |
| CID2807595 | cluster0 |
| CID2812173 | cluster0 |
| CID2987927 | cluster0 |
| CID3001028 | cluster2 |
| CID3001055 | cluster2 |
| CID3005572 | cluster2 |
| CID3006531 | cluster2 |
| CID3025986 | cluster1 |
| CID3031661 | cluster0 |
| CID3032581 | cluster0 |
| CID3034010 | cluster2 |
| CID3037617 | cluster2 |
| CID3038522 | cluster1 |
| CID3052775 | cluster3 |
| CID3062316 | cluster2 |
| CID3070537 | cluster0 |
| CID3082777 | cluster0 |
| CID3084046 | cluster0 |
| CID3733518 | cluster2 |
| CID4998669 | cluster3 |
| CID5001396 | cluster1 |
| CID5222465 | cluster3 |
| CID5280343 | cluster2 |
| CID5280360 | cluster2 |
| CID5280453 | cluster2 |
| CID5280483 | cluster2 |
| CID5280961 | cluster2 |
| CID5281004 | cluster2 |
| CID5281040 | cluster1 |
| CID5281051 | cluster2 |
| CID5281078 | cluster2 |
| CID5281321 | cluster3 |
| CID5281614 | cluster2 |
| CID5281767 | cluster2 |
| CID5281828 | cluster0 |
| CID5281877 | cluster0 |
| CID5281888 | cluster2 |
| CID5281955 | cluster2 |
| CID5282375 | cluster2 |
| CID5282379 | cluster2 |
| CID5282381 | cluster2 |
| CID5282412 | cluster0 |
| CID5282451 | cluster0 |
| CID5284344 | cluster2 |
| CID5284371 | cluster0 |
| CID5284380 | cluster0 |
| CID5284513 | cluster2 |
| CID5284558 | cluster2 |
| CID5284566 | cluster0 |
| CID5284616 | cluster2 |
| CID5287969 | cluster2 |
| CID5288382 | cluster2 |
| CID5288628 | cluster1 |
| CID5288783 | cluster3 |
| CID5288826 | cluster2 |
| CID5289247 | cluster2 |
| CID5289419 | cluster1 |
| CID5311051 | cluster0 |
| CID5311181 | cluster2 |
| CID5311263 | cluster2 |
| CID5311497 | cluster2 |
| CID5311498 | cluster0 |
| CID5323510 | cluster3 |
| CID5327044 | cluster0 |
| CID5328779 | cluster2 |
| CID5329006 | cluster1 |
| CID5329032 | cluster1 |
| CID5329098 | cluster2 |
| CID5330175 | cluster1 |
| CID5352019 | cluster0 |
| CID5353562 | cluster2 |
| CID5353586 | cluster2 |
| CID5353980 | cluster2 |
| CID5359264 | cluster0 |
| CID5359282 | cluster0 |
| CID5359476 | cluster2 |
| CID5360515 | cluster2 |
| CID5360621 | cluster0 |
| CID5362420 | cluster2 |
| CID5362440 | cluster2 |
| CID5362564 | cluster0 |
| CID5381226 | cluster2 |
| CID5386092 | cluster0 |
| CID5458428 | cluster2 |
| CID5460555 | cluster0 |
| CID5462328 | cluster2 |
| CID5469318 | cluster2 |
| CID5472495 | cluster2 |
| CID5474206 | cluster2 |
| CID5476374 | cluster0 |
| CID5477614 | cluster0 |
| CID5479530 | cluster0 |
| CID5480431 | cluster2 |
| CID5481350 | cluster2 |
| CID5484731 | cluster0 |
| CID5487525 | cluster0 |
| CID5494407 | cluster0 |
| CID5494424 | cluster0 |
| CID5702553 | cluster0 |
| CID6091659 | cluster2 |
| CID6167828 | cluster0 |
| CID6323490 | cluster0 |
| CID6324671 | cluster2 |
| CID6419957 | cluster0 |
| CID6420135 | cluster0 |
| CID6432013 | cluster0 |
| CID6433557 | cluster2 |
| CID6435110 | cluster0 |
| CID6438891 | cluster0 |
| CID6439072 | cluster1 |
| CID6440175 | cluster2 |
| CID6442177 | cluster2 |
| CID6444692 | cluster2 |
| CID6445533 | cluster2 |
| CID6445540 | cluster0 |
| CID6445562 | cluster3 |
| CID6450551 | cluster2 |
| CID6450813 | cluster1 |
| CID6456015 | cluster0 |
| CID6505803 | cluster2 |
| CID6540268 | cluster0 |
| CID6540295 | cluster0 |
| CID6603857 | cluster1 |
| CID6604200 | cluster2 |
| CID6713928 | cluster0 |
| CID6850715 | cluster0 |
| CID6850726 | cluster0 |
| CID6850753 | cluster0 |
| CID6914628 | cluster2 |
| CID6917781 | cluster2 |
| CID6918220 | cluster0 |
| CID6918250 | cluster0 |
| CID6918289 | cluster2 |
| CID6918296 | cluster2 |
| CID6918365 | cluster0 |
| CID6918403 | cluster2 |
| CID6918412 | cluster3 |
| CID6918453 | cluster2 |
| CID6918454 | cluster2 |
| CID6918456 | cluster2 |
| CID6918508 | cluster1 |
| CID6918537 | cluster0 |
| CID6918558 | cluster3 |
| CID6918638 | cluster2 |
| CID6918837 | cluster2 |
| CID9547169 | cluster0 |
| CID9547917 | cluster0 |
| CID9549284 | cluster0 |
| CID9549299 | cluster2 |
| CID9562060 | cluster2 |
| CID9568512 | cluster0 |
| CID9796068 | cluster0 |
| CID9800306 | cluster2 |
| CID9804302 | cluster3 |
| CID9809714 | cluster1 |
| CID9825149 | cluster3 |
| CID9826528 | cluster2 |
| CID9832447 | cluster2 |
| CID9843206 | cluster1 |
| CID9849735 | cluster1 |
| CID9854073 | cluster3 |
| CID9863342 | cluster3 |
| CID9865515 | cluster2 |
| CID9868037 | cluster1 |
| CID9868524 | cluster1 |
| CID9874912 | cluster1 |
| CID9881652 | cluster3 |
| CID9913881 | cluster1 |
| CID9914412 | cluster1 |
| CID9915743 | cluster1 |
| CID9926791 | cluster2 |
| CID9933475 | cluster1 |
| CID9977819 | cluster1 |
| CID10027278 | cluster2 |
| CID10096043 | cluster0 |
| CID10113978 | cluster1 |
| CID10126189 | cluster2 |
| CID10127622 | cluster2 |
| CID10138259 | cluster1 |
| CID10172943 | cluster1 |
| CID10218498 | cluster0 |
| CID10302451 | cluster0 |
| CID10317566 | cluster0 |
| CID10319891 | cluster0 |
| CID10322450 | cluster0 |
| CID10384072 | cluster1 |
| CID10437018 | cluster1 |
| CID10458325 | cluster0 |
| CID10460379 | cluster2 |
| CID10461815 | cluster1 |
| CID10953556 | cluster3 |
| CID11154925 | cluster1 |
| CID11167602 | cluster1 |
| CID11196273 | cluster0 |
| CID11210478 | cluster3 |
| CID11228183 | cluster2 |
| CID11234052 | cluster1 |
| CID11282283 | cluster2 |
| CID11349170 | cluster3 |
| CID11364421 | cluster1 |
| CID11404337 | cluster1 |
| CID11414799 | cluster0 |
| CID11485656 | cluster1 |
| CID11494412 | cluster2 |
| CID11511120 | cluster3 |
| CID11520894 | cluster0 |
| CID11534420 | cluster0 |
| CID11578515 | cluster2 |
| CID11599950 | cluster0 |
| CID11646823 | cluster0 |
| CID11647372 | cluster1 |
| CID11655119 | cluster0 |
| CID11667893 | cluster0 |
| CID11671467 | cluster1 |
| CID11679764 | cluster0 |
| CID11707110 | cluster2 |
| CID11713159 | cluster1 |
| CID11714998 | cluster0 |
| CID11717001 | cluster1 |
| CID11822705 | cluster0 |
| CID11953947 | cluster0 |
| CID11966249 | cluster0 |
| CID11966311 | cluster0 |
| CID11970251 | cluster0 |
| CID11977753 | cluster2 |
| CID11987672 | cluster0 |
| CID13783824 | cluster0 |
| CID13922196 | cluster0 |
| CID14476155 | cluster0 |
| CID14942883 | cluster0 |
| CID15950351 | cluster1 |
| CID16129975 | cluster0 |
| CID16130049 | cluster2 |
| CID16134956 | cluster0 |
| CID16217590 | cluster0 |
| CID16725726 | cluster1 |
| CID16736529 | cluster0 |
| CID16759369 | cluster1 |
| CID16760281 | cluster0 |
| CID16760691 | cluster2 |
| CID17754438 | cluster1 |
| CID17755052 | cluster3 |
| CID18991124 | cluster0 |
| CID20042692 | cluster3 |
| CID20620240 | cluster1 |
| CID22024915 | cluster1 |
| CID23624255 | cluster0 |
| CID23674191 | cluster0 |
| CID23690938 | cluster0 |
| CID23724531 | cluster0 |
| CID23725625 | cluster0 |
| CID24180719 | cluster2 |
| CID24756910 | cluster1 |
| CID24764437 | cluster0 |
| CID24764449 | cluster1 |
| CID24771867 | cluster1 |
| CID24776445 | cluster2 |
| CID24779724 | cluster3 |
| CID24783227 | cluster0 |
| CID24785538 | cluster1 |
| CID24794418 | cluster1 |
| CID24838940 | cluster0 |
| CID24840378 | cluster3 |
| CID24892733 | cluster0 |
| CID24901704 | cluster1 |
| CID24901722 | cluster0 |
| CID24941245 | cluster0 |
| CID24956525 | cluster2 |
| CID24964624 | cluster1 |
| CID24978514 | cluster0 |
| CID24978538 | cluster3 |
| CID25027363 | cluster0 |
| CID25033539 | cluster3 |
| CID25088416 | cluster0 |
| CID25102847 | cluster2 |
| CID25126797 | cluster0 |
| CID25167777 | cluster1 |
| CID25171647 | cluster0 |
| CID25182616 | cluster1 |
| CID26275995 | cluster2 |
| CID27885548 | cluster2 |
| CID28777137 | cluster0 |
| CID42611257 | cluster2 |
| CID42642645 | cluster1 |
| CID44134894 | cluster2 |
| CID44137945 | cluster1 |
| CID44182395 | cluster3 |
| CID44187953 | cluster1 |
| CID44228987 | cluster3 |
| CID44263835 | cluster0 |
| CID44268108 | cluster2 |
| CID44284481 | cluster0 |
| CID44472890 | cluster0 |
| CID44483210 | cluster0 |
| CID44593851 | cluster0 |
| CID44604932 | cluster0 |
| CID44607530 | cluster3 |
| CID45100498 | cluster0 |
| CID45142457 | cluster0 |
| CID45270897 | cluster2 |
| CID45382213 | cluster0 |
| CID45480163 | cluster0 |
| CID46216795 | cluster0 |
| CID46885626 | cluster1 |
| CID46911863 | cluster0 |
| CID49769060 | cluster0 |
| CID49769423 | cluster0 |
| CID49837887 | cluster1 |
| CID49867926 | cluster0 |
| CID51346199 | cluster0 |
| CID53346510 | cluster0 |
| CID53352191 | cluster0 |
| CID53384665 | cluster0 |
| CID53427792 | cluster0 |
| CID53477714 | cluster2 |
| CID53629505 | cluster2 |
| CID53630776 | cluster0 |
| CID53630877 | cluster0 |
| CID54609526 | cluster0 |
| CID54684141 | cluster3 |
| CID54710406 | cluster3 |
| CID54734719 | cluster0 |
| CID56603655 | cluster2 |
| CID56603668 | cluster2 |
| CID56649450 | cluster0 |
| CID56684138 | cluster0 |
| CID56841764 | cluster0 |
| CID56841800 | cluster0 |
| CID56841999 | cluster0 |
| CID56842042 | cluster0 |
| CID56842082 | cluster0 |
| CID56842117 | cluster2 |
| CID56842118 | cluster0 |
| CID56842121 | cluster2 |
| CID56842157 | cluster0 |
| CID56843240 | cluster0 |
| CID56952026 | cluster0 |
| CID57390074 | cluster0 |
| CID66553073 | cluster0 |
| CID70679302 | cluster0 |
| CID70680240 | cluster0 |
| CID70683023 | cluster0 |
| CID71297189 | cluster0 |
| CID71308162 | cluster2 |
| CID71463576 | cluster0 |
| CID71464519 | cluster0 |

#: Anti-NSCLC drug
