# Supplementary material for: A computational method for the identification of candidate drugs for non-small cell lung cancer
Source: PLoS One. 2017 Aug 18;12(8):e0183411. doi: 10.1371/journal.pone.0183411 (PMC5562320; doi:10.1371/journal.pone.0183411)
Supplement: S6 Table — (DOCX) [file pone.0183411.s006.docx]

**S6 Table.** Clustering results by the EM algorithm on 98 candidate compounds and ten approved NSCLC drugs

| **PubChem ID** | **Category** |
| --- | --- |
| CID60843^#^ | cluster3 |
| CID57166^#^ | cluster3 |
| CID4033^#^ | cluster3 |
| CID38904^#^ | cluster3 |
| CID36314^#^ | cluster3 |
| CID176870^#^ | cluster3 |
| CID126941^#^ | cluster3 |
| CID123631^#^ | cluster3 |
| CID11626560^#^ | cluster3 |
| CID10184653^#^ | cluster3 |
| CID15 | cluster0 |
| CID271 | cluster4 |
| CID702 | cluster4 |
| CID1032 | cluster0 |
| CID1060 | cluster0 |
| CID1112 | cluster0 |
| CID1153 | cluster4 |
| CID1401 | cluster1 |
| CID2776 | cluster4 |
| CID2794 | cluster1 |
| CID3121 | cluster1 |
| CID3922 | cluster1 |
| CID3954 | cluster1 |
| CID4189 | cluster1 |
| CID4679 | cluster1 |
| CID4707 | cluster1 |
| CID4708 | cluster4 |
| CID4971 | cluster3 |
| CID4973 | cluster1 |
| CID5381 | cluster1 |
| CID5834 | cluster4 |
| CID6256 | cluster1 |
| CID6742 | cluster0 |
| CID7187 | cluster1 |
| CID8977 | cluster2 |
| CID10465 | cluster0 |
| CID10635 | cluster0 |
| CID11103 | cluster3 |
| CID12733 | cluster1 |
| CID13945 | cluster1 |
| CID18343 | cluster1 |
| CID18407 | cluster0 |
| CID20469 | cluster1 |
| CID22986 | cluster0 |
| CID30751 | cluster1 |
| CID31254 | cluster1 |
| CID31402 | cluster1 |
| CID33113 | cluster2 |
| CID33641 | cluster1 |
| CID39981 | cluster4 |
| CID43805 | cluster1 |
| CID57469 | cluster1 |
| CID61635 | cluster4 |
| CID64968 | cluster1 |
| CID65063 | cluster4 |
| CID65091 | cluster1 |
| CID65110 | cluster4 |
| CID68770 | cluster1 |
| CID72435 | cluster4 |
| CID89105 | cluster1 |
| CID93004 | cluster1 |
| CID102288 | cluster0 |
| CID107744 | cluster1 |
| CID108007 | cluster0 |
| CID119031 | cluster1 |
| CID119196 | cluster4 |
| CID119373 | cluster2 |
| CID123606 | cluster1 |
| CID124331 | cluster2 |
| CID145729 | cluster1 |
| CID148201 | cluster1 |
| CID156413 | cluster3 |
| CID159854 | cluster1 |
| CID160913 | cluster4 |
| CID208908 | cluster3 |
| CID216453 | cluster4 |
| CID275196 | cluster1 |
| CID394347 | cluster2 |
| CID446378 | cluster0 |
| CID3052775 | cluster1 |
| CID4998669 | cluster0 |
| CID5222465 | cluster1 |
| CID5281321 | cluster4 |
| CID5288783 | cluster4 |
| CID5323510 | cluster4 |
| CID6445562 | cluster3 |
| CID6918412 | cluster1 |
| CID6918558 | cluster1 |
| CID9804302 | cluster4 |
| CID9825149 | cluster1 |
| CID9854073 | cluster1 |
| CID9863342 | cluster4 |
| CID9881652 | cluster1 |
| CID10953556 | cluster0 |
| CID11210478 | cluster1 |
| CID11349170 | cluster4 |
| CID11511120 | cluster3 |
| CID17755052 | cluster4 |
| CID20042692 | cluster2 |
| CID24779724 | cluster2 |
| CID24840378 | cluster1 |
| CID24978538 | cluster1 |
| CID25033539 | cluster2 |
| CID44182395 | cluster4 |
| CID44228987 | cluster4 |
| CID44607530 | cluster2 |
| CID54684141 | cluster4 |
| CID54710406 | cluster4 |

#: Anti-NSCLC drug
